# Microwave-assisted synthesis of (aminomethylene)bisphosphine oxides and (aminomethylene)bisphosphonates by a three-component condensation

**DOI:** 10.3762/bjoc.12.146

**Published:** 2016-07-19

**Authors:** Erika Bálint, Ádám Tajti, Anna Dzielak, Gerhard Hägele, György Keglevich

**Affiliations:** 1 MTA-BME Research Group for Organic Chemical Technology, 1521 Budapest, Hungary; 2 Department of Organic Chemistry and Technology, Budapest University of Technology and Economics, 1521 Budapest, Hungary; 3 Institute of Inorganic Chemistry, Heinrich-Heine-University Düsseldorf, 40225 Düsseldorf, Germany

**Keywords:** (aminomethylene)bisphosphine oxides, (aminomethylene)bisphosphonates, microwave, three-component condensation

## Abstract

A practical method was elaborated for the synthesis of (aminomethylene)bisphosphine oxides comprising the catalyst- and solvent-free microwave-assisted three-component condensation of primary amines, triethyl orthoformate and two equivalents of diphenylphosphine oxide. The method is also suitable for the preparation of (aminomethylene)bisphosphonates using (MeO)_2_P(O)H/(MeO)_3_CH or (EtO)_2_P(O)H/(EtO)_3_CH reactant pairs and even secondary amines. Several intermediates referring to the reaction mechanism together with a few by-products could also be identified.

## Introduction

Substituted (hydroxymethylene)bisphosphonic acid derivatives form an important group of drugs used in the treatment of osteoporosis and related bone diseases [1–3]. In the last decades, at least three generations of dronic acid derivatives appeared [4].

(Aminomethylene)bisphosphonic acid derivatives are analogous species, that also have potential bioactivity in bone diseases, besides they display antibacterial, antiparasitic, anticancer and herbicidal activities [5].

(Aminomethylene)bisphosphonates may be prepared in different ways [5]. One of the most convenient and widespread methods is the three-component condensation involving an amine, an orthoformate and a dialkyl phosphite. Usually, primary or secondary amines were reacted with an equivalent, or a small excess of triethyl orthoformate and 2–7 equivalents of diethyl phosphite [6–21]. In most cases, the corresponding acids were the target molecules that were obtained by hydrolysis of the esters [15–21]. The use of crown ethers with an NH unit, or thienopyrimidine amines as starting materials was also reported [22–23]. The catalyst- and solvent-free methods required long reaction times and/or a high temperature [6–14,21–23]. Ionic liquids and a few catalysts were also tried out [24–27], and the synthesis was also described under microwave (MW) irradiation [28–32]. However, most of the MW-assisted syntheses were performed in kitchen ovens [28–30], hence these results cannot be reproduced.

**Scheme 1:**
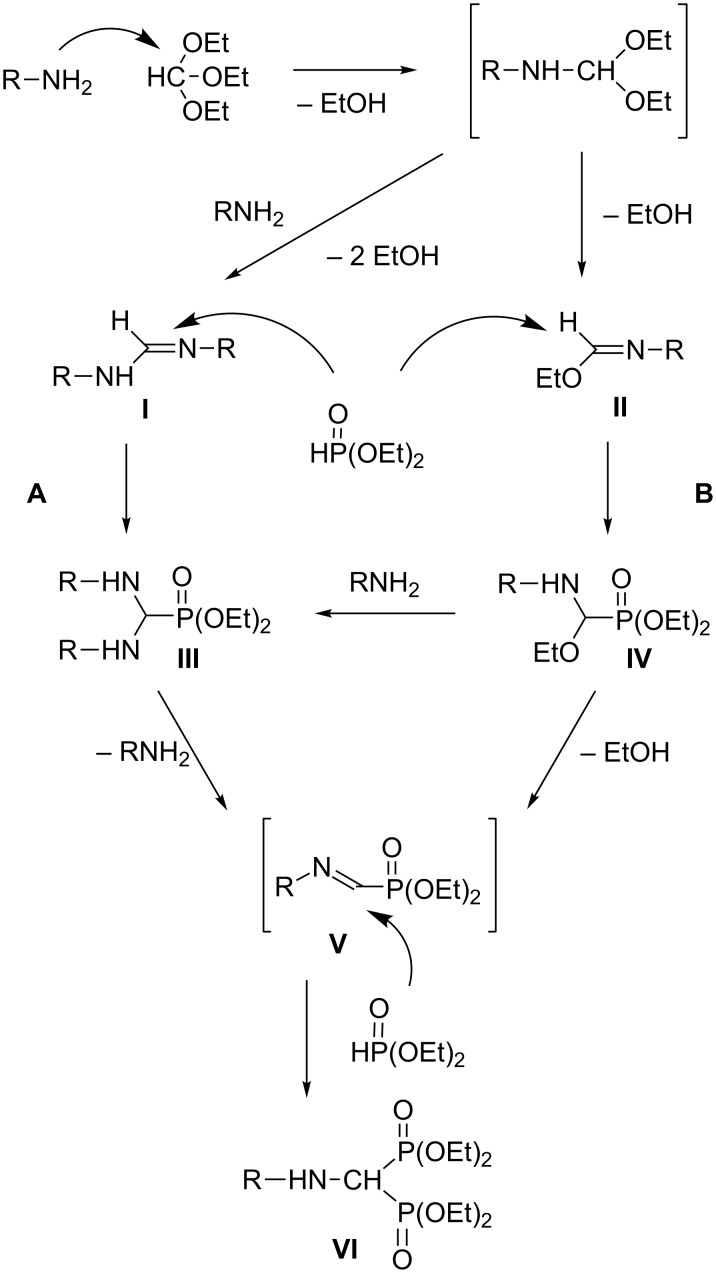
Proposed routes for the three-component condensation [7].

The mechanism of the three-component condensation has been investigated by the research group of Krutikov and Kafarski [6–7]. A detailed proposal is shown in Scheme 1 [7]. The first step of the condensation is the reaction of the amine with the orthoformate, in which imine-type intermediates **I** or **II** may be formed. The next step is the nucleophilic addition of diethyl phosphite to the C=N bond of the imines resulting in phosphonates **III** or **IV**, respectively. Then, the elimination of an amine or ethanol and the addition of another unit of diethyl phosphite may lead to (aminomethylene)bisphosphonates (**VI**). If the amine is in predominance over the phosphite in the reaction, the pathway **A** is more likely, but if the phosphite is used in excess, the pathway **B** comes to the fore.

**Scheme 2:**
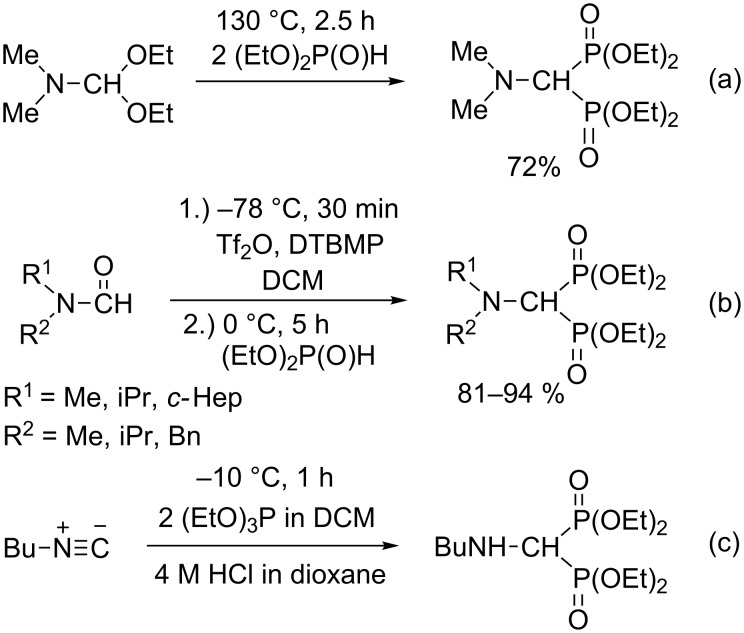
Synthetic methods for (aminomethylene)bisphosphonates I.

**Scheme 3:**
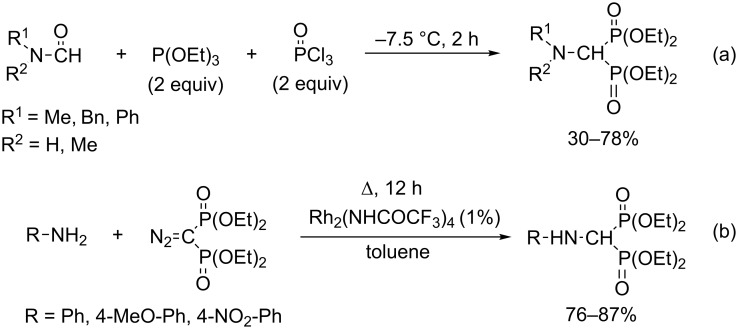
Synthetic methods for (aminomethylene)bisphosphonates II.

**Scheme 4:**
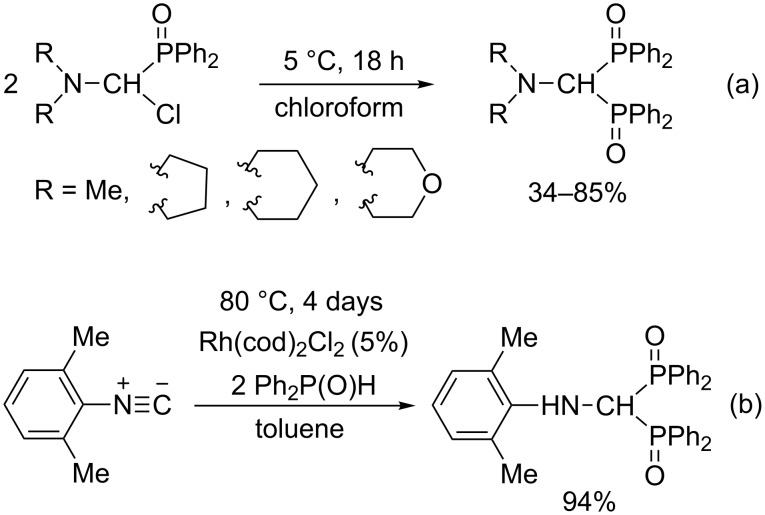
Synthetic methods for (aminomethylene)bisphosphine oxides.

There are other possibilities to synthesize (aminomethylene)bisphosphonates, such as by the reaction of dimethylformamide diethyl acetal with diethyl phosphite (Scheme 2a) [33], by the condensation of formamides and diethyl phosphite using 2,6-di-*tert*-butyl-4-methylpyridine (DTBMP) as the base, and trifluoromethanesulfonic anhydride (Tf_2_O) as the catalyst (Scheme 2b) [34], or by the reaction of isonitriles with triethyl phosphite (Scheme 2c) [35–36]. (Aminomethylene)bisphosphonates can also be obtained starting from amides, triethyl phosphite and phosphorus oxychloride (Scheme 3a) [37], or in the reaction of amines with diazophosphonate in the presence of a rhodium catalyst (Scheme 3b) [38].

(Aminomethylene)bisphosphine oxides are analogous to (aminomethylene)bisphosphonates, but they are much less studied. Only a few publications were found, which focus on their synthesis [33,39–42], however, a three-component condensation has not been described. They can also be prepared starting from dimethylformamide dimethyl acetal, as in the synthesis of (aminomethylene)bisphosphonates, but in the latter case a secondary phosphine oxide is the P-reagent [33,39]. In addition, (aminomethylene)bisphosphine oxides can be synthesized by the reaction of two molecules of (dialkylamino)(diphenylphosphinoyl)chloromethane (Scheme 4a) [40–41], or by the addition of diphenylphosphine oxide to an isonitrile (Scheme 4b) [36,42].

In this paper, we wish to report the results of our investigations on the synthetic protocol utilizing the three-component condensations of primary or secondary amines, orthoformates and >P(O)H species, such as dialkyl phosphites or diphenylphosphine oxide, and we aimed at the preparation of new derivatives.

## Results and Discussion

### Synthesis of alkylamino- and (phenylaminomethylene)bisphosphine oxides

**Scheme 5:**
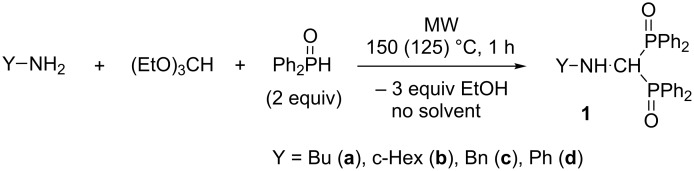
Synthesis of alkylamino- and (phenylaminomethylene)bisphosphine oxides.

In the first series of experiments, the condensation of primary amines, such as butyl-, cyclohexyl- and benzylamine or aniline with triethyl orthoformate, and 2 equivalents of diphenylphosphine oxide at 150 °C for 1 h under MW conditions was studied (Scheme 5). To avoid the formation of by-products, benzylamine was reacted at a lower temperature of 125 °C (Table 1, entry 3). The reactions were carried out without any catalyst and solvent. After column chromatography, the new amino-methylenebisphosphine oxides **1a–d** were obtained in yields of 72–82% (Table 1, entries 1–4).

**Table 1:** Synthesis of alkylamino- and (phenylaminomethylene)bisphosphine oxides **1a–d**.

Entry	Y	*T* (°C)	Yield (%)^a^

1	Bu	150	82 (**1a**)
2	c-Hex	150	79 (**1b**)
3	Bn	125^b^	72 (**1c**)
4	Ph	150	80 (**1d**)

^a^Isolated yield. ^b^At 150 °C by-products were formed.

**Scheme 6:**
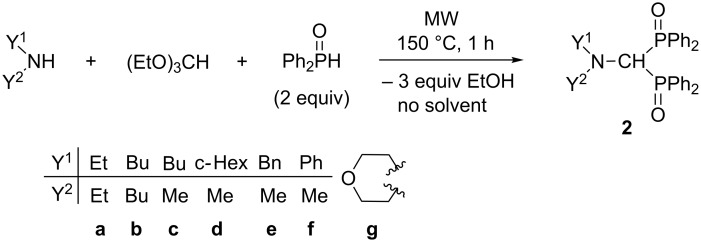
Synthesis of (dialkylaminomethylene)bisphosphine oxides.

The condensation of simple secondary amines (diethyl-, dibutyl-, *N*-butylmethyl-, *N*-cyclohexylmethyl-, *N*-benzylmethylamine, *N*-methylaniline and morpholine) was also investigated with triethyl orthoformate, and 2 equivalents of diphenylphosphine oxide (Scheme 6, Table 2). The MW-assisted reactions were performed at 150 °C for 1 h under solvent- and catalyst-free conditions, and the (dialkylaminomethylene)bisphosphine oxides **2a–g** were obtained in yields of 60–85% after column chromatography (Table 2, entries 1–7). Except for compound **2g**, all (aminomethylene)bisphosphine oxides (**2a–f**) prepared are new compounds. According to the literature method [41], **2g** was synthesized by the reaction of two molecules of (diphenylphosphinoyl)morpholinochloromethane in the presence of chloroform at 5 °C for 18 h in a yield of 41% (Scheme 4a). Using the MW-assisted three-component condensation method, this compound (**2g**) can be synthesized without any catalyst and solvent in a short time (1 h), and in a yield of 85% (Table 2, entry 7).

**Table 2:** Synthesis of (dialkylaminomethylene)bisphosphine oxides **2a–g**.

Entry	Y^1^	Y^2^	Yield (%)^a^

1	Et	Et	82 (**2a**)
2	Bu	Bu	73 (**2b**)
3	Bu	Me	69 (**2c**)
4	c-Hex	Me	66 (**2d**)
5	Bn	Me	64 (**2e**)
6	Ph	Me	60 (**2f**)
7	-(CH_2_)_2_-O-(CH_2_)_2_-		85 (**2g**)

^a^Isolated yield.

### Synthesis of alkylamino- and (phenylaminomethylene)bisphosphonates

In the next stage, our method was extended to the synthesis of alkyl- and (phenylaminomethylene)bisphosphonates by reacting butyl- and cyclohexylamine or aniline, and triethyl orthoformate with diethyl phosphite under MW irradiation in the absence of catalyst and solvent (Table 3). First, the condensation of butylamine, triethyl orthoformate with 2 equivalents of diethyl phosphite was studied at 125 °C. After a 2 h’s reaction time, the conversion was 91%, and beside the expected (aminomethylene)bisphosphonate **3a** formed in 81%, the *N*-ethylated by-product **4a** was formed in 19% (Table 3, entry 1). Increasing the temperature to 150 °C, the reaction was completed after 30 min, but the proportion of the main product **3a** was somewhat lower (78%), and another by-product **5a** also appeared in 7% (Table 3, entry 2). The target compound **3a** could be obtained in a yield of 61%. Using 3.5 equivalents of diethyl phosphite at 125 °C for 1 h, the conversion was only 75%, but the expected product **3a** was formed exclusively (Table 3, entry 3). After a longer reaction time of 1.5 h, by-product **4a** also appeared in 22% (Table 3, entry 4). In the reaction with cyclohexylamine, the same tendency was observed (Table 3, entries 5–8), and the corresponding (cyclohexylaminomethylene)bisphosphonate **3b** was obtained in a yield of 68% after column chromatography (Table 3, entry 6). Finally, the three-component condensation of aniline, triethyl orthoformate and diethyl phosphite was studied (Table 3, entries 9–11). Applying 2 equivalents of phosphite, the reaction was not complete, neither at 125 °C, nor at 150 °C (Table 3, entries 9 and 10). Two types of imine intermediates (**6a** and **6b**) could be observed in the reaction mixture beside the expected product **3c**. These intermediates refer to the mechanism of the condensation (see compounds **I** and **V** in Scheme 1, pathway A). Previously, iminephosphonate **6b** was only an assumed intermediate [7], but now we could prove it by ^31^P NMR and HRMS (Table 4). Increasing the amount of diethyl phosphite to 3 equivalents, the reaction was complete at 125 °C after 1 h, and only **3c** was formed with a yield of 82% (Table 3, entry 11). In the cases discussed, no ethylated or formylated by-products (**4** and **5**, respectively) were formed. (Aminomethylene)bisphosphonates **3b** and **3c** were synthesized earlier in unoptimized experiments to provide compounds **3b** and **3c** in yields of 36% [9] and 53% [8], respectively. The former compound **3b** was characterized only by ^1^H NMR [9]. Compound **3c** was also synthesized under MW irradiation in a yield of 75% [31]. It can be seen, that the refined MW-assisted method elaborated by us may give the (aminomethylene)bisphosphonates **3b** and **3c** in yields of 68% and 82%, respectively.

**Table 3:** The reactions of primary amines with triethyl orthoformate and diethyl phosphite.

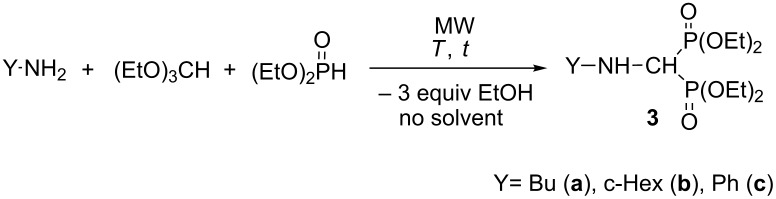									

Entry	Y	DEP (equiv)	*T* (°C)	*t* (h)	Conversion (%)^a^	Product composition (%)^a^			Yield of **3** (%)^c^
						**3**	by-products^b^		

							**4**	**5**	

1	Bu	2	125	2	91	81	19	0	**–**
2	Bu	2	150	0.5	100	78	15	7	61 (**3a**)
3	Bu	3.5	125	1	75	100	0	0	–
4	Bu	3.5	125	1.5	90	78	22	0	–
5	c-Hex	2	125	2	76	83	17	0	–
6	c-Hex	2	150	0.5	100	88	10	2	68 (**3b**)
7	c-Hex	3.5	125	1	63	100	0	0	–
8	c-Hex	3.5	125	1.5	83	86	14	0	–
9	Ph	2	125	2	68	56^d^	0	0	36 (**3c**)
10	Ph	2	150	1	90	70^d^	0	0	52 (**3c**)
11	Ph	3	125	1	100	100	0	0	82 (**3c**)

^a^On the basis of GC (entries 1–8) or on the basis of HPLC (entries 9–11). ^b^The by-products identified:
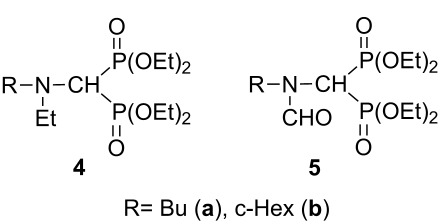
^c^Isolated yield. ^d^The following intermediates were also formed based on LC–MS:
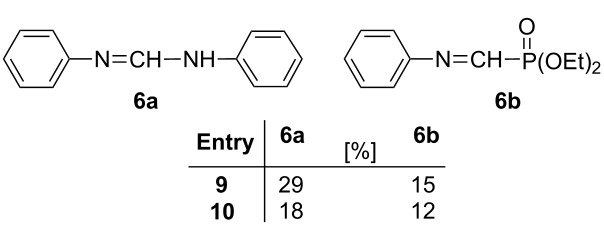

**Table 4:** Spectral characterization of *N*-ethyl- (**4**) and *N*-formyl- (**5**) (aminomethylene)bisphosphonates and imine-type intermediates **6a** and **6b**.

Compounds	δ_P_ in CDCl_3_	δ_P_ [lit.]	[M + H]^+^_found_	[M + H]^+^_requires_

**4a**	19.98	–	388.2020	388.2012
**4b**	20.63	–	414.2162	414.2169
**5a**	16.08 and 16.16 (*E* and *Z* isomers)	15.69 and 15.98^a^ (*E* and *Z* isomers) [43]	388.1659	388.1649
**5b**	16.00 and 16.06 (*E* and *Z* isomers)	–	414.1797	414.1805
**6a**	–	–	197.1075	197.1073
**6b**	17.67	–	242.0936	242.0941

^a^In CCl_4_.

**Scheme 7:**
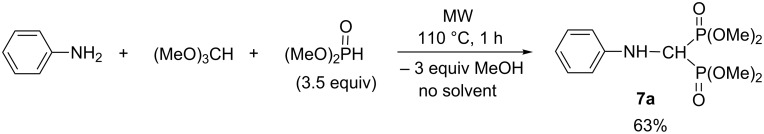
Synthesis of tetramethyl (phenylaminomethylene)bisphosphonate.

Next, the condensation of aniline with trimethyl orthoformate and dimethyl phosphite was also performed (Scheme 7). In this case, the reaction was complete after a 1 h heating at 110 °C using 3.5 equivalents of dimethyl phosphite. After column chromatography, the corresponding product **7a** was isolated in a yield of 63%. At higher temperatures, decomposition was observed.

**Scheme 8:**
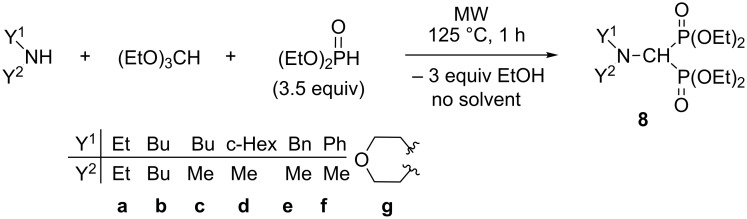
Synthesis of (dialkylaminomethylene)bisphosphonates.

In the next stage, the MW-assisted reaction of secondary amines was studied with triethyl orthoformate and diethyl phosphite (Scheme 8). The condensations were carried out applying 3.5 equivalents of diethyl phosphite at 125 °C for 1 h in the absence of a catalyst and a solvent. In case of *N*-methylaniline, 4.5 equivalents of the P-reagent was necessary to attain complete conversion (Table 5, entry 6). The corresponding (dialkylaminomethylene)bisphosphonates (**8a–g**) were obtained in yields of 65–86% after purification by chromatography (Table 5, entries 1–7).

**Table 5:** Synthesis of (dialkylaminomethylene)bisphosphonates **8a–g**.

Entry	Y^1^	Y^2^	Yield (%)^a^

1	Et	Et	86 (**8a**)
2	Bu	Bu	68 (**8b**)
3	Bu	Me	79 (**8c**)
4	c-Hex	Me	72 (**8d**)
5	Bn	Me	70 (**8e**)^b^
6^c^	Ph	Me	65 (**8f**)
7	-(CH_2_)_2_-O-(CH_2_)_2_-		81 (**8g**)^d^

^a^Isolated yield. ^b^It was synthesized in a yield of 61% [10]. ^c^4.5 equivalents of diethyl phosphonate was used.^d^It was synthesized in a yield of 46% [4].

**Figure 1:**
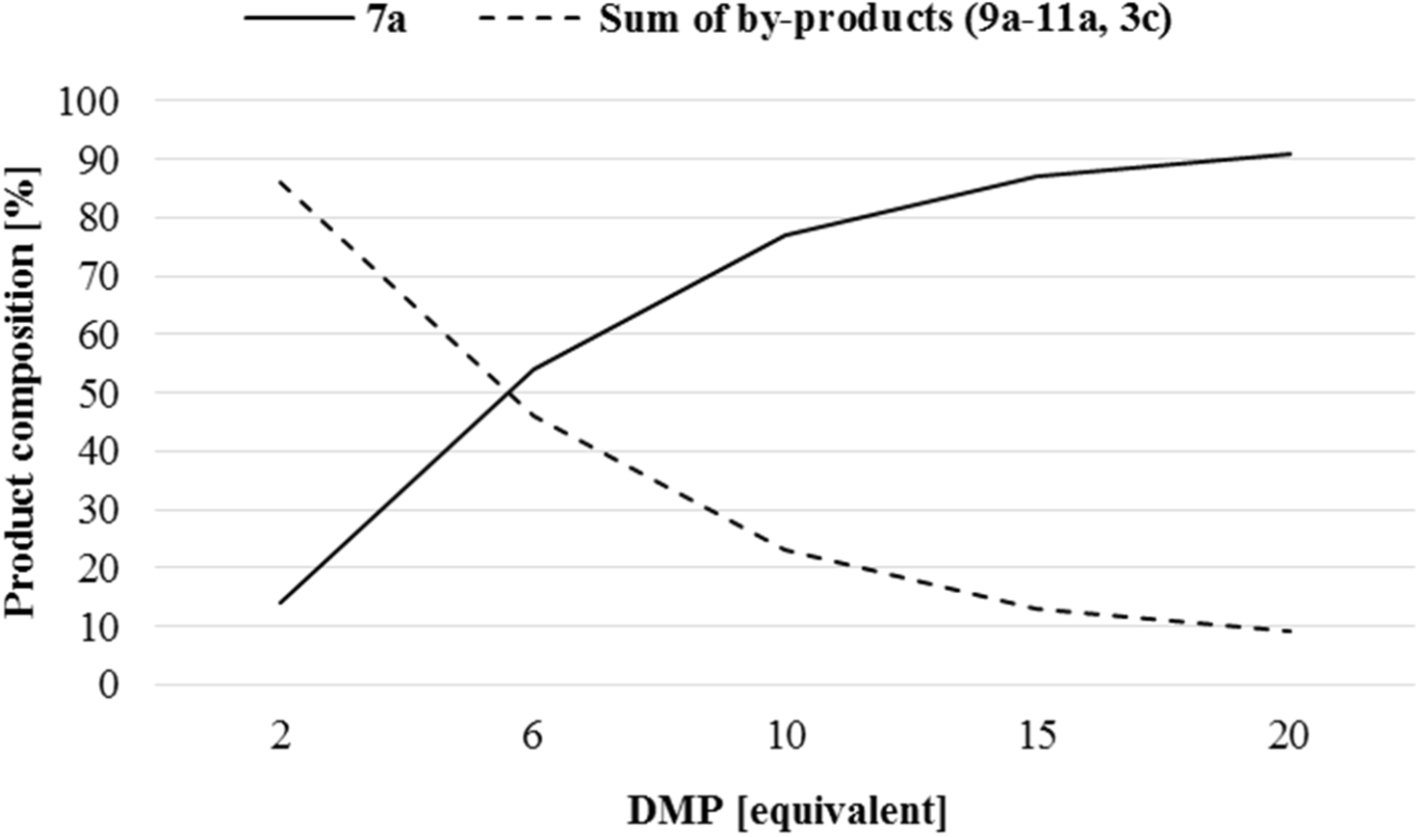
Effect of the quantity of dimethyl phosphite (DMP) on the product composition (from Table 6, entries 1–5.)

**Figure 2:**
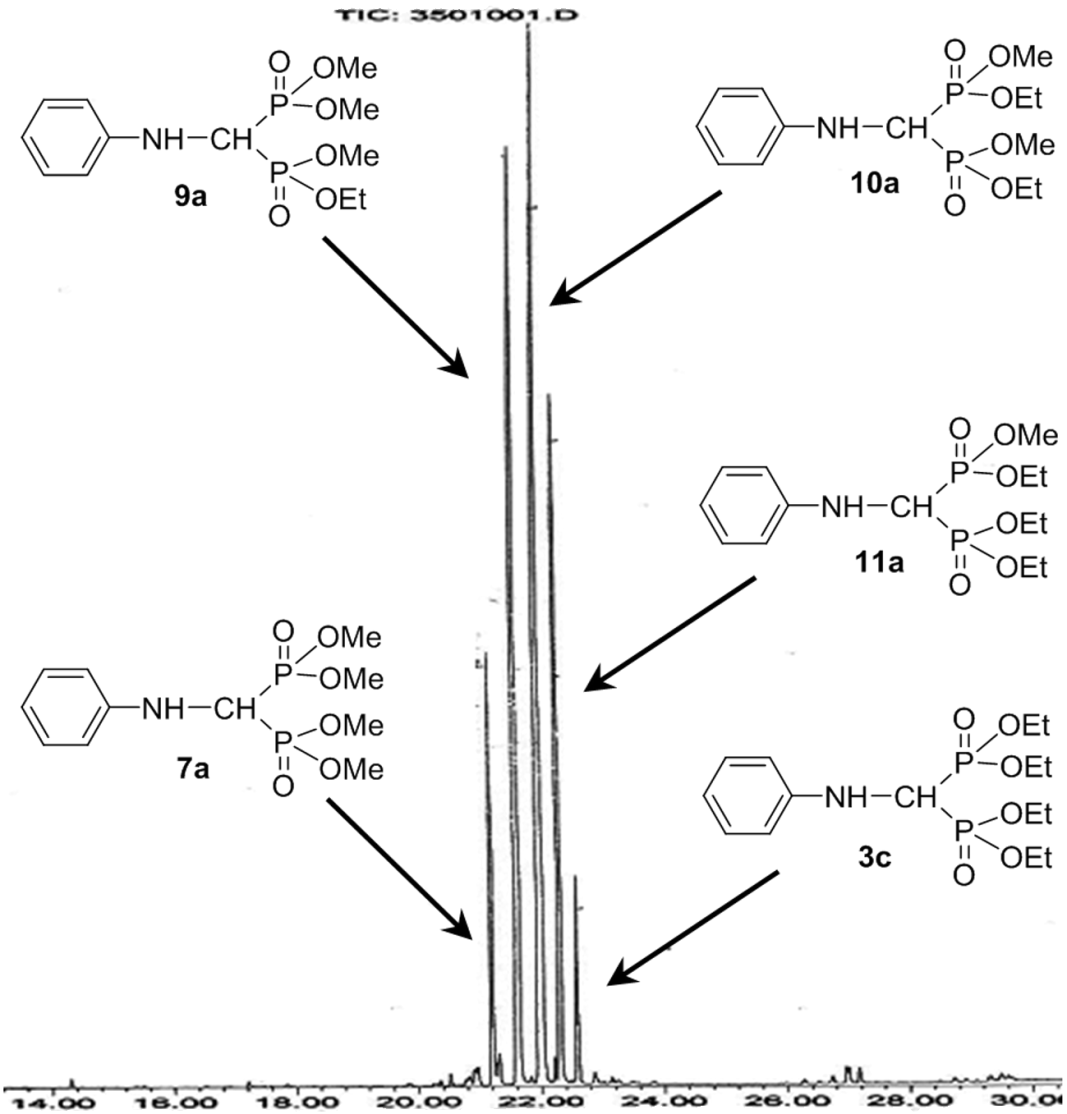
GC–MS chromatogram for the condensation of aniline, triethyl orthoformate and 2 equivalents of dimethyl phosphite (from the exp. marked by Table 6, entry 1).

**Figure 3:**
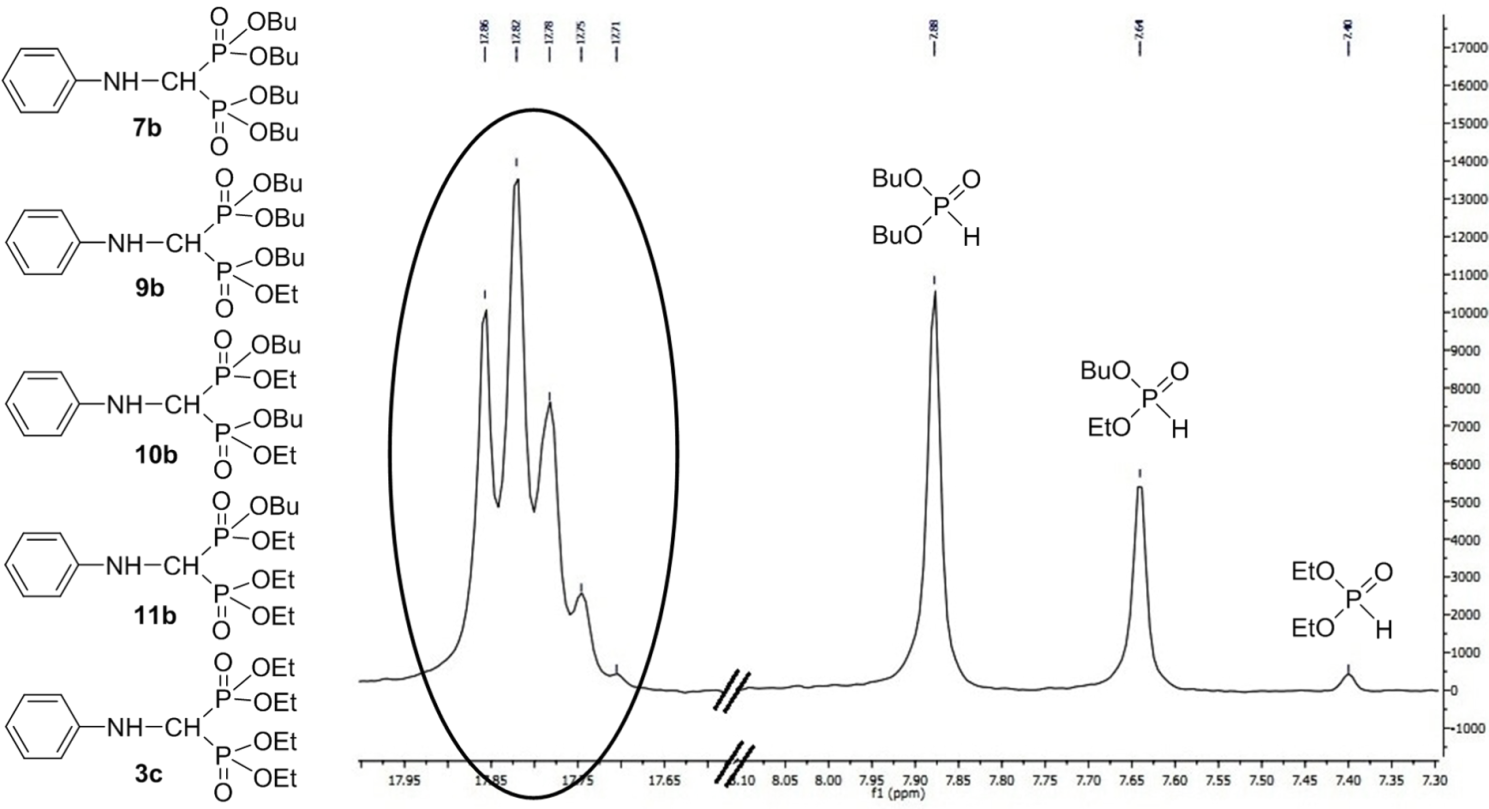
^31^P NMR spectrum for the condensation of aniline, triethyl orthoformate and 2 equivalents of dibutyl phosphite (from the exp. marked by Table 6, entry 6).

Finally, the three-component condensation of aniline, triethyl orthoformate and dimethyl or dibutyl phosphite was studied (Table 6, Figure 1). Using dimethyl phosphite, the reactions were performed at 110 °C for 1 h, but in case of dibutyl phosphite, the conditions applied were the same as those in the condensations with diethyl phosphite (125–150 °C, 1 h). Using 2 equivalents of dialkyl phosphite, more or less transesterified (aminomethylene)bisphosphonates (**9–11** and **3c**) were also formed beside the expected (phenylaminomethylene)bisphosphonates **7a** or **7b** (Table 6,entries 1 and 6, 7). The transesterified by-products (**9-11** and **3c**) were indentified by GC–MS (Figure 2) or LC–MS, and were proved by HRMS (Table 7). The composition of the reaction mixture for the experiment marked by Table 6, entry 6 was analyzed by ^31^P NMR (see Figure 3). It was observed that increasing the quantity of dialkyl phosphite, the proportion of the by-products was decreased, and the condensations became more selective for the desired product (**7a** or **7b**) (Table 6, Figure 1). In the reaction with dimethyl phosphite, the best result was achieved using 20 equivalents of the P-reagent, but in case of dibutyl phosphite, a 15-fold excess was sufficient (Table 6, entries 5 and 10).

**Table 6:** Condensation of aniline, triethyl orthoformate and dimethyl or dibutyl phosphite.

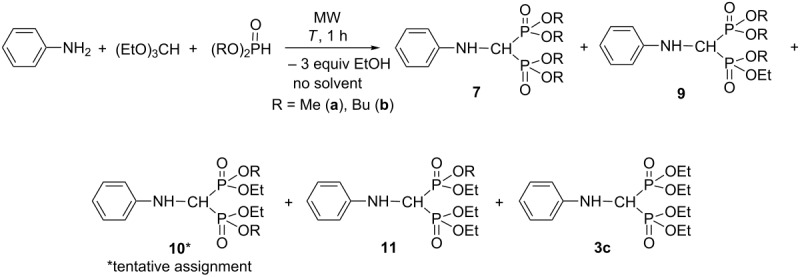								

Entry	R	Dialkyl phosphite (equiv)	*T* (°C)	Product composition (%)^a^				
				**7**	**9**	**10**	**11**	**3c**

1	Me	2	110	14	36	36	11	3
2	Me	6	110	54	37	9	0	0
3	Me	10	110	77	18	5	0	0
4	Me	15	110	87	13	0	0	0
5	Me	20^b^	110	**91**	9	0	0	0
6	Bu	2	150	19	23	29	26	3
7	Bu	2	125	54	33	10	3	0
8	Bu	6	125	82	16	2	0	0
9	Bu	10	125	93	7	0	0	0
10	Bu	15^b^	125	**95**	5	0	0	0

^a^On the basis of GC (entries 1–5) or on the basis of HPLC (entries 6–9). ^b^The product composition has not changed using a larger excess of dialkyl phosphite.

**Table 7:** Mass spectral characterization of (aminomethylene)bisphosphonates.

	R = Me (b)		R = Bu (c)	
	[M + H]^+^_found_	[M + H]^+^_requires_	[M + H]^+^_found_	[M + H]^+^_requires_

**7**	324.0760	324.0760	492.2648	492.2638
**9**	338.0921	338.0917	464.2329	464.2325
**10**	352.1072	352.1073	436.2011	436.2012
**11**	366.1231	366.1230	408.1711	408.1699
**3c**	380.1382	380.1386	380.1382	380.1386

## Conclusion

In summary, we have developed a facile, solvent- and catalyst-free MW-assisted method for the synthesis of (aminomethylene)bisphosphine oxides (AMBPOs) and (aminomethylene)bisphosphonates by the condensation of a primary or secondary amine, an orthoformate, and diphenylphosphine oxide or a dialkyl phosphite. This method is a novel approach for the preparation of AMBPOs and an optimized process for the synthesis of (aminomethylene)bisphosphonates. Twenty-two derivatives were isolated and characterized, except two, all of them are new compounds. Furthermore, a few intermediates supporting the mechanism of the condensation, and several by-products were also identified.

## Supporting Information

Experimental procedures, characterization data, details of the NMR structural determination of all products and copies of ^31^P, ^1^H, and ^13^C NMR spectra for all compounds synthesized are presented in Supporting Information File 1.

File 1 Experimental, NMR spectra.

## References

[1] Breuer E, Fischer J, Ganelli C R (2006). The Development of Bisphosphonates as Drugs. Analogue-based Drug Discovery.

[2] Russell R G G (2007). Pediatrics.

[3] Russell R G G (2011). Bone.

[4] Hudson H R, Wardle N J, Bligh S W A, Greiner I, Grün A, Keglevich G (2012). Mini-Rev Med Chem.

[5] Romanenko V D, Kukhar V P (2012). ARKIVOC.

[6] Krutikov V I, Erkin A V, Pautov P A, Zolotukhina M M (2003). Russ J Gen Chem.

[7] Dąbrowska E, Burzyńska A, Mucha A, Matczak-Jon E, Sawka-Dobrowolska W, Berlicki Ł, Kafarski P (2009). J Organomet Chem.

[8] Tauro M, Laghezza A, Loiodice F, Agamennone M, Campestre C, Tortorella P (2013). Bioorg Med Chem.

[9] Takeuchi M, Sakamoto S, Yoshida M, Abe T, Isomura Y (1993). Chem Pharm Bull.

[10] Ekimoto H (2010). Metal Complex Compound, Cancer Therapeutic Composition Comprising the Metal Complex Compound as Active Ingredient, and Intermediate for Production of the Metal Complex Compound. Eur. Pat..

[11] Rose Y S, Ciblat S, Kang T, Far A R, Dietrich E, Lafontaine Y, Reddy R (2011). Phosphonated Rifamycins and Uses Thereof or the Prevention and Treatment of Bone and Joint Infections. U.S. Patent.

[12] Zhang Q M, Serpe M J (2014). Macromolecules.

[13] Kantoci D, Denike J K, Wechter W J (1996). Synth Commun.

[14] Kubíček V, Rudovský J, Kotek J, Hermann P, Vander Elst L, Muller R N, Kolar Z I, Wolterbeek H T, Peters J A, Lukeš I (2005). J Am Chem Soc.

[15] Forlani G, Occhipinti A, Berlicki Ł, Dziedzioła G, Wieczorek A, Kafarski P (2008). J Agric Food Chem.

[16] Martin M B, Grimley J S, Lewis J C, Heath H T, Bailey B N, Kendrick H, Yardley V, Caldera A, Lira R, Urbina J A (2001). J Med Chem.

[17] Widler L, Jaeggi K A, Glatt M, Müller K, Bachmann R, Bisping M, Born A-R, Cortesi R, Guiglia G, Jeker H (2002). J Med Chem.

[18] Forlani G, Berlicki Ł, Duò M, Dziędzioła G, Giberti S, Bertazzini M, Kafarski P (2013). J Agric Food Chem.

[19] Kotsikorou E, Song Y, Chan J M W, Faelens S, Tovian Z, Broderick E, Bakalara N, Docampo R, Oldfield E (2005). J Med Chem.

[20] Parniak M, Mellors J W, Oldfield E, Tovian Z, Chan J M W (2005). Composition and Methods for Use of Antiviral Drugs in the Treatment of Retroviral Diseases Resistant to Nucleoside Reverse Transcriptase Inhibitors. U.S. Patent Appl..

[21] Chmielewska E, Mazur Z, Kempińska K, Wietrzyk J, Piątek A, Kuryszko J J, Kiełbowicz Z, Kafarski P (2015). Phosphorus, Sulfur Silicon Relat Elem.

[22] Fallouh F, Bernier D, Virieux D, Cristau H J, Pirat J L (2006). Phosphorus, Sulfur Silicon Relat Elem.

[23] Leung C-Y, Langille A M, Mancuso J, Tsantrizos Y S (2013). Bioorg Med Chem.

[24] Reddy M V, Kalla R M N, Dong L S, Jeong Y T (2015). Catal Commun.

[25] Kunda U M R, Balam S K, Nemallapudi B R, Chereddy S S, Nayak S K, Cirandur S R (2012). Chem Pharm Bull.

[26] Reddy M V N, Kim J, Jeong Y T (2012). J Fluorine Chem.

[27] Prasad S S, Jayaprakash S H, Syamasundar C, Sreelakshmi P, Bhuvaneswar C, Bhaskar B V, Rajendra W, Nayak S K, Reddy C S (2015). Phosphorus, Sulfur Silicon Relat Elem.

[28] Minaeva L I, Patrikeeva L S, Kabachnik M M, Beletskaya I P, Orlinson B S, Novakov I A (2011). Heteroat Chem.

[29] Minaeva L I, Kabachnik M M, Ponomarev G V, Morozova J V, Beletskaya I P (2010). Synthesis.

[30] Reddy G C S, Reddy M V N, Reddy N B, Reddy C S (2010). Phosphorus, Sulfur Silicon Relat Elem.

[31] Kaboudin B, Alipour S (2009). Tetrahedron Lett.

[32] Lacbay C M, Mancuso J, Lin Y-S, Bennett N, Götte M, Tsantrizos Y S (2014). J Med Chem.

[33] Prishchenko A A, Livantsov M V, Novikova O P, Livantsova L I, Erschov I S, Petrosyan V S (2015). Heteroat Chem.

[34] Wang A-E, Chang Z, Sun W-T, Huang P-Q (2015). Org Lett.

[35] Goldeman W, Kluczyński A, Soroka M (2012). Tetrahedron Lett.

[36] Pudovik A N, Nikitina V I, Zimin M G, Vostretsova N L (1975). J Gen Chem USSR.

[37] Olive G, Jacques A (2003). Phosphorus, Sulfur Silicon Relat Elem.

[38] Lecerclé D, Gabillet S, Gomis J-M, Taran F (2008). Tetrahedron Lett.

[39] Gross H, Costisella B (1969). J Prakt Chem.

[40] Morgalyuk V P, Strelkova T V, Nifant'ev E E (2012). Bull Chem Soc Jpn.

[41] Morgalyuk V P, Strelkova T V, Nifant'ev E E (2012). Russ Chem Bull.

[42] Hirai T, Han L-B (2006). J Am Chem Soc.

[43] Costisella B, Gross H (1979). J Prakt Chem.

