# Supplementary material for: Microwave-assisted synthesis of (aminomethylene)bisphosphine oxides and (aminomethylene)bisphosphonates by a three-component condensation
Source: Beilstein J Org Chem. 2016 Jul 19;12:1493–502. doi: 10.3762/bjoc.12.146 (PMC4979732; doi:10.3762/bjoc.12.146)
Supplement: File 1 — Experimental, NMR spectra. [file Beilstein_J_Org_Chem-12-1493-s001.pdf]

Supporting Information

for

Microwave-assisted synthesis of (aminomethylene)bisphosphine oxides and (aminomethylene)bisphosphonates by a three-component condensation

Erika Bálint*[1], Ádám Tajti[2], Anna Dzielak[2], Gerhard Hägele[3] and György Keglevich*[2]

Address: [1]MTA-BME Research Group for Organic Chemical Technology, 1521 Budapest, Hungary, [2]Department of Organic Chemistry and Technology, Budapest University of Technology and Economics, 1521 Budapest, Hungary and [3]Institute of Inorganic Chemistry, Heinrich-Heine-University Düsseldorf, 40225 Düsseldorf, Germany

Email: Erika Bálint* - ebalint@mail.bme.hu; György Keglevich* - gkeglevich@mail.bme.hu

*Corresponding author

Table of contents

General information	S2
General procedure for the synthesis of (aminomethylene)bisphosphine oxides	S2–S6
General procedure for the synthesis of (aminomethylene)bisphosphonates	S7–S8
General procedure for the synthesis of (dialkylaminomethylene)bisphosphonates	S8–S11
^{31}P NMR, ^{1}H NMR and ^{13}C NMR spectra	S12–S45
References	S46

General information

The ^{31}P, ^{13}C, ^{1}H NMR spectra were taken in $CDCl_3$ solution on a Bruker AV-300 or DRX-500 spectrometer operating at 121.5, 75.5 and 300 or 202.4, 125.7 and 500 MHz, respectively. Chemical shifts are reported downfield relative to 85% H_3PO_4 or TMS. The ^{13}C NMR chemical shifts were assigned on the basis of analogies by comparable model compounds [S1]. The coupling constants *J* are given in Hz. NMR spectra were evaluated using the computer programs WINNMR and TOPSPIN from Bruker. Non-equivalence effects were observed in ^{1}H and $^{13}C\{^{1}H\}$ NMR spectra. Corresponding pairs of resonances were marked with (I) and (II), respectively. Mass spectrometric measurements were performed using a Q-TOF Premier mass spectrometer in positive electrospray mode and a Shimadzu LCMS-ITTOF mass spectrometer. The reactions were carried out in a 300 W CEM Discover focused microwave reactor equipped with a pressure controller applying 15–100 W under isothermal conditions.

General procedure for the synthesis of (aminomethylene)bisphosphine oxides

A mixture of 0.50 mmol primary amine (*n*-butylamine: 0.05 ml, cyclohexylamine: 0.06 ml, benzylamine: 0.055 ml, aniline: 0.05 ml) or 0.50 mmol secondary amine (diethylamine: 0.05 ml, dibutylamine: 0.08 ml, *N*-butyl-*N*-methylamine: 0.06 ml, *N*-cyclohexyl-*N*-methylamine: 0.065 ml, *N*-benzyl-*N*-methylamine: 0.065 ml, *N*-methylaniline: 0.05 ml, morpholine: 0.04 ml), 0.50 mmol (0.08 ml) of triethyl orthoformate, and 1.0 mmol (0.20 g) of diphenylphosphine oxide was heated at 125 or 150 °C under N_2 in a closed vial in a CEM Discover Microwave reactor equipped with a pressure controller applying 50–100 W for 1 h. The crude product so obtained was purified on silica gel using dichloromethane/methanol (99:1) as the eluent. After evaporation of the solvent, the products (**1a**–**d**, **2a**–**g**) were obtained as crystals. The following products were thus prepared:

(Butylaminomethylene)bis(diphenylphosphine oxide) (1a)

Yield: 82% (0.20 g) of compound **1a** as white crystals; Mp: 154-155 °C; ^{31}P NMR ($CDCl_3$) δ 26.82; ^{1}H NMR ($CDCl_3$) δ 0.675 (t, $^{3}J_{HH}$ = 7.1, 3 H, CH_3), 0.94-1.22 (m, 4 H, C-CH_2CH_2-C), 2.146 (s, 1 H, N*H*), 2.22-2.34 (m, 2 H, CH_2N), 4.145 (t, $^{2}J_{PH}$ = -15.3 Hz, 1 H, C*H*P_2), 7.24-7.50 (m, 12 H, H_{para} and H_{ortho}, P-C_6H_5), 7.74-7.85 (m, 4 H, H_{meta}, (I), P-C_6H_5), 7.87-7.98 (m, 4 H, H_{meta}, (II), P-C_6H_5); ^{1}H NMR (C_6D_6) δ 0.628 (t, $^{3}J_{HH}$ = 7.2, 3 H, CH_3), 0.91-1.16 (m, 4H, C-CH_2CH_2-C), 2.296 (t, $^{3}J_{HH}$ = 6.7, 2 H, CH_2N), 2.575 (s, br, 1H, N*H*), 4.264 (t, $^{2}J_{PH}$ = -15.4 Hz, 1 H, C*H*P_2), 6.92-7.08 (m, 12 H, H_{para} and H_{ortho}, P-C_6H_5), 8.01-8.15 (m, 4 H, H_{meta},

(I), P-C_6H_5), 8.17-8.30 (m, 4 H, H_{meta}, (II), P-C_6H_5); ^{13}C NMR ($CDCl_3$) δ 13.76 (s, 1C, $CH_3CH_2CH_2$), 19.91 (s, 1C, $CH_3CH_2CH_2$), 32.10 (s, 1C, $CH_3CH_2CH_2$), 51.85 (t, $^3J_{PC}$ = 3.7, 1C, CH_2N), 61.75 (t, $^1J_{PC}$ = -65.9, 1C, CHP_2), 128.01 (t, $^2J_{PC}$ = 6.0, 4C, C_{ortho}, (I), P-C_6H_5), 128.24 (t, $^2J_{PC}$ = 6.4, 4C, C_{ortho}, (I), P-C_6H_5), 131.63 (m, br, 4C, C_{para}, P-C_6H_5), 131.63 (t, 4C, $^3J_{PC}$ = 4.5, C_{meta}, (I), P-C_6H_5), 131.84 (m, N = 101.1, 2C, C_{ipso}, (I), P-C_6H_5), 131.87 (t, 4C, $^3J_{PC}$ = 4.7, C_{meta}, (II), P-C_6H_5), 131.90 (m, N = 99.9, 2C, C_{ipso}, (II), P-C_6H_5); $[M+H]^+_{found}$ = 488.1890, $C_{29}H_{32}NO_2P_2$ requires 488.1903.

(Cyclohexylaminomethylene)bis(diphenylphosphine oxide) (1b)

Yield: 79% (0.20 g) of compound **1b** as white crystals; Mp: 171-172 °C; ^{31}P NMR ($CDCl_3$) δ 26.86; ^{1}H NMR ($CDCl_3$) δ 0.59-1.00 (m, 5 H, c-C_6H_{11}), 1.30-1.49 (m, 5 H, c-C_6H_{11}), 1.82-1.93 (m, 1 H, c-C_6H_{11}), 2.256 (s, 1 H, NH), 4.262 (t, $^2J_{PH}$ = -15.2 Hz, 1 H, C$H$$P_2$), 7.22-7.49 (m, 12 H, H_{para} and H_{ortho}, P-C_6H_5), 7.73-7.83 (m, 4 H, H_{meta}, (I), P-C_6H_5), 7.88-7.99 (m, 4 H, H_{meta}, (II), P-C_6H_5); ^{13}C NMR ($CDCl_3$) δ 24.73 (s, 2C, cHx), 25.76 (s, 1C, cHx), 32.82 (s, 2C, cHx), 57.63 (t, $^3J_{PC}$ = 3.7, 1C, C_1, cHx), 59.38 (t, $^1J_{PC}$ = -65.4, 1C, CHP_2), 127.91 (t, $^2J_{PC}$ = 5.9, 4C, C_{ortho}, (I), P-C_6H_5), 128.24 (t, $^2J_{PC}$ = 5.8, 4C, C_{ortho}, (II), P-C_6H_5), 131.60 (m, 4C, C_{para}, P-C_6H_5), 131.60 (t, $^3J_{PC}$ = 4.7, 4C, C_{meta}, (I), P-C_6H_5), 132.04 (t, $^3J_{PC}$ = 4.7, 4C, C_{meta}, (II), P-C_6H_5); $[M+H]^+_{found}$ = 514.2049, $C_{31}H_{34}NO_2P_2$ requires 514.2059.

(Benzylaminomethylene)bis(diphenylphosphine oxide) (1c)

Yield: 72% (0.19 g) of compound **1c** as white crystals; Mp: 167-168 °C; ^{31}P NMR ($CDCl_3$) δ 27.46; ^{1}H NMR ($CDCl_3$) δ 2.617 (dt, br, $^3J_{HH}$ = 7.5, $^3J_{HH}$ = 5.2, 1 H, NH), 3.457 (d, $^3J_{HH}$ = 5.2, 2 H, CH_2N), 4.290 (dt, $^3J_{HH}$ = 7.2, $^2J_{PH}$ = -14.9, 1 H, C$H$$P_2$), 6.89-6.79 (m, 2 H, H_{ortho}, C-C_6H_5), 7.08-7.20 (m, 3 H, H_{meta} and H_{para}, C-C_6H_5), 7.26-7.49 (m, 12 H, H_{ortho} and H_{para}, P-C_6H_5), 7.73-7.83 (m, 4 H, H_{meta}, (I), P-C_6H_5), 7.83-7.94 (m, 4 H, H_{meta}, (II), P-C_6H_5); ^{13}C NMR ($CDCl_3$) δ 55.37 (t, $^3J_{PC}$ = 4.4, 1C, CH_2N), 60.65 (t, $^1J_{PC}$ = -65.1, 1C, CHP_2), 127.29 (s, 1C, C_{para}, C-C_6H_5), 128.15 (t, $^2J_{PC}$ = 6.5, 4C, C_{ortho}, (I), P-C_6H_5), 128.25 (s, 2C, C_{meta}, C-C_6H_5), 128.34 (t, $^2J_{PC}$ = 6.5, 4C, C_{ortho}, (II), P-C_6H_5), 128.70 (s, 2C, C_{ortho}, C-C_6H_5), 131.64 (t, $^3J_{PC}$ = 4.7, 4C, C_{meta}, (I), P-C_6H_5), 131.70 (s, 4C, C_{para}, P-C_6H_5), 131.92 (t, $^3J_{PC}$ = 4.7, 4C, C_{meta}, (II), P-C_6H_5), 138.46 (s, 1C, C_{ipso}, C-C_6H_5); $[M+H]^+_{found}$ = 522.1763, $C_{32}H_{30}NO_2P_2$ requires 522.1746.

(Phenylaminomethylene)bis(diphenylphosphine oxide) (1d)

Yield: 80% (0.20 g) of compound **1d** as pale yellow crystals; Mp: 257-258 °C; ^{31}P NMR ($CDCl_3$) δ 28.01; ^{1}H NMR ($CDCl_3$) δ 4.656 (dt, br, $^{3}J_{HH}$ = 9.6 Hz, $^{3}J_{PH}$ = 4.5 Hz, 1 H, N*H*), 5.151 (dt, $^{3}J_{HH}$ = 9.9 Hz, $^{2}J_{PH}$ = -12.9 Hz, 1 H, C*H*P_2), 6.284 (d, $^{3}J_{HH}$ = 7.9, 2 H, H_{ortho}, N-C_6H_5), 6.560 (t, $^{3}J_{HH}$ = 7.3, 1 H, H_{para}, N-C_6H_5), 6.872 (t, $^{3}J_{HH}$ = 7.8, 2 H, H_{meta}, N-C_6H_5), 7.21-7.48 (m, 12 H, H_{para} and H_{ortho}, P-C_6H_5), 7.73-7.91 (m, 8 H, H_{meta}, P-C_6H_5); ^{13}C NMR ($CDCl_3$) δ 57.19 (t, $^{1}J_{PC}$ = -65.1, 1C, *C*HP_2), 114.12 (s, 2C, C_{ortho}, C-C_6H_5), 119.08 (s, 1C, C_{para}, C-C_6H_5), 128.27 (t, $^{2}J_{PC}$ = 6.0, 4C, C_{ortho}, (I), P-C_6H_5), 128.29 (t, $^{2}J_{PC}$ = 6.0, 4C, C_{ortho}, (II), P-C_6H_5), 128.88 (s, 2C, C_{meta}, C-C_6H_5), 131.62 (t, $^{3}J_{PC}$ = 4.9, 4C, C_{meta}, (I), P-C_6H_5), 131.86 (t, $^{3}J_{PC}$ = 4.8, 4C, C_{meta}, (II), P-C_6H_5), 131.95 (t, $^{4}J_{PC}$ = 1.7, 2C, C_{para}, (I), P-C_6H_5), 131.97 (t, $^{4}J_{PC}$ = 1.6, 2C, C_{para}, (II), P-C_6H_5), (difficult to locate: C_{ipso}, P-C_6H_5), 146.07 (t, $^{3}J_{PC}$ = 2.8, 1C, C_{ipso}, C-C_6H_5); $[M+H]^{+}_{found}$ = 508.1579, $C_{31}H_{28}NO_2P_2$ requires 508.1590.

(Diethylaminomethylene)bis(diphenylphosphine oxide) (2a)

Yield: 82% (0.20 g) of compound **2a** as white crystals; Mp: 214-215 °C; ^{31}P NMR ($CDCl_3$) δ 28.85; ^{1}H NMR ($CDCl_3$) δ 0.688 (t, $^{3}J_{HH}$ = 7.0 Hz, 6 H, C*H*$_3$CH_2N), 2.861 (q, $^{3}J_{HH}$ = 7.1 Hz, 4 H, CH_3C*H*$_2$N), 4.900 (t, $^{2}J_{PH}$ = -16.8 Hz, 1 H, C*H*P_2), 7.22-7.46 (m, 12 H, H_{para} and H_{ortho}, P-C_6H_5), 7.72-7.84 (m, 4 H, H_{meta}, (I), P-C_6H_5), 7.92-8.03 (m, 4H, H_{meta}, (II), P-C_6H_5); ^{13}C NMR ($CDCl_3$) δ 14.28 (s, 2C, *C*H_3CH_2), 48.09 (t, $^{3}J_{PC}$ = 3.2, 2C, CH_3*C*H_2), 68.09 (t, $^{1}J_{PC}$ = -61.1, 1C, *C*HP_2), 127.99 (t, $^{2}J_{PC}$ = 6.1, 4C, C_{ortho}, (I), P-C_6H_5), 128.16 (t, $^{2}J_{PC}$ = 6.1, 4C, C_{ortho}, (II), P-C_6H_5), 131.39 (br, 4C, C_{para}, P-C_6H_5), 131.74 (t, $^{3}J_{PC}$ = 4.8, 4C, C_{meta}, (I), P-C_6H_5), 131.87 (t, $^{3}J_{PC}$ = 4.9, 4C, C_{meta}, (II), P-C_6H_5), 132.85 (m, N = 93.0, 2C, C_{ipso}, (I), P-C_6H_5), 133.39 (m, N = 96.8, 2C, C_{ipso}, (I), P-C_6H_5); $[M+H]^{+}_{found}$ = 488.1886, $C_{29}H_{32}NO_2P_2$ requires 488.1903.

(Dibutylaminomethylene)bis(diphenylphosphine oxide) (2b)

Yield: 73% (0.20 g) of compound **2b** as white crystals; Mp: 133-134 °C; ^{31}P NMR ($CDCl_3$) δ 28.95; ^{1}H NMR ($CDCl_3$) δ 0.709 (t, $^{3}J_{HH}$ = 6.8 Hz, 6 H, C*H*$_3$), 0.87-1.10 (m, 8 H, C-C*H*$_2$C*H*$_2$-C), 2.780 (t, $^{3}J_{HH}$ = 7.2 Hz, 4 H, C*H*$_2$N), 4.866 (t, $^{2}J_{PH}$ = -16.4 Hz, 1 H, C*H*P_2), 7.22-7.45 (m, 12 H, H_{para} and H_{ortho}, P-C_6H_5), 7.73-7.83 (m, 4 H, H_{meta}, (I), P-C_6H_5), 7.90-8.00 (m, 4 H, H_{meta}, (II), P-C_6H_5); ^{13}C NMR ($CDCl_3$) δ 13.93 (s, 2C, *C*H_3CH_2CH_2), 20.21 (s, 2C, CH_3*C*H_2CH_2), 31.43 (s, 2C, CH_3CH_2*C*H_2), 54.94 (br, 2C, *C*H_2N), 68.82 (t, $^{1}J_{PC}$ = -61.1, 1C, *C*HP_2), 127.99 (t, $^{2}J_{PC}$ = 5.5, 4C, C_{ortho}, (I), P-C_6H_5), 128.13 (t, $^{2}J_{PC}$ = 5.6, 4C, C_{ortho}, (II), P-C_6H_5), 131. 39 (br, 4C, C_{para}, P-C_6H_5), 131.74 (t, $^{3}J_{PC}$ = 4.9, 4C, C_{meta}, (I), P-C_6H_5), 131.88

(t, $^3J_{PC}$ = 5.0, 4C, C_{meta}, (II), P-C_6H_5), 132.97 (m, *N* = 92.4, 2C, C_{ipso}, (I), P-C_6H_5), 133.53 (m, *N* = 96.8, 2C, C_{ipso}, (II), P-C_6H_5); $[M+H]^+_{found}$ = 544.2523, $C_{33}H_{40}NO_2P_2$ requires 544.2529.

(*N*-Butyl-*N*-methylaminomethylene)bis(diphenylphosphine oxide) (2c)

Yield: 69% (0.17 g) of compound **2c** as white crystals; Mp: 194-195 °C; ^{31}P NMR ($CDCl_3$) δ 28.52; ^{1}H NMR ($CDCl_3$) δ 0.705 (t, $^3J_{HH}$ = 7.1 Hz, 3 H, CH_3), 0.85-1.13 (m, 4 H, C-CH_2CH_2-C), 2.428 (s, br, 3 H, NCH_3), 2.801 (t, $^3J_{HH}$ = 7.4 Hz, 2 H, CH_2N), 4.725 (t, $^2J_{PH}$ = -17.2 Hz, 1 H, C$H$$P_2$), 7.22-7.52 (m, 12 H, H_{para} and H_{ortho}, P-C_6H_5), 7.73-7.85 (m, 4 H, H_{meta}, (I), P-C_6H_5), 7.94-8.50 (m, 4 H, H_{meta}, (II), P-C_6H_5); ^{13}C NMR ($CDCl_3$) δ 13.97 (s, 1C, CH_3), 20.02 (s, 1C, CH_3CH_2), 30.73 (s, 1C, $CH_3CH_2CH_2$), 41.21 (m, br, 1C, NCH_3), 57.80 (m, br, 1C, CH_2N), 70.72 (t, $^1J_{PC}$ = -61.7, 1C, CHP_2), 128.10 (t, $^2J_{PC}$ = 6.0, 4C, C_{ortho}, (I), P-C_6H_5), 128.26 (t, $^2J_{PC}$ = 6.0, 4C, C_{ortho}, (II), P-C_6H_5), 131.53 (m, br, 4C, C_{para}, P-C_6H_5), 131.88 (t, $^3J_{PC}$ = 4.9, 4C, C_{meta}, (I), P-C_6H_5), 131.94 (t, $^3J_{PC}$ = 4.9, 4C, C_{meta}, (II), P-C_6H_5), 133.29 (m, *N* = 94.3, 2C, C_{ipso}, (I), P-C_6H_5), 133.36 (m, *N* = 97.4, 2C, C_{ipso}, (II), P-C_6H_5); $[M+H]^+_{found}$ = 502.2048, $C_{30}H_{34}NO_2P_2$ requires 502.2059.

(*N*-Cyclohexyl-*N*-methylaminomethylene)bis(diphenylphosphine oxide) (2d)

Yield: 66% (0.17 g) of compound **2d** as white crystals; Mp: 194-195 °C; ^{31}P NMR ($CDCl_3$) δ 29.78; ^{1}H NMR ($CDCl_3$) δ 0.80-1.16 (m, 5 H, c-C_6H_{11}), 1.42-1.53 (m, 1 H, c-C_6H_{11}), 1.53-1.72 (m, 4 H, c-C_6H_{11}), 2.383 (s, 3 H, NCH_3), 2.648 (t, $^3J_{HH}$ = 10.6 Hz, 1 H, NCH), 4.942 (t, $^2J_{PH}$ = -17.5 Hz, 1 H, C$H$$P_2$), 7.23-7.45 (m, 12 H, H_{para} and H_{ortho}, P-C_6H_5), 7.74-7.86 (m, 4 H, H_{meta}, (I), P-C_6H_5), 7.94-8.05 (m, 4 H, H_{meta}, (II), P-C_6H_5); ^{13}C NMR ($CDCl_3$) δ 25.75 (s, 2C, cHx), 25.82 (s, 1C, cHx), 31.12 (s, 2C, cHx), 36.56 (t, br, $^3J_{PC}$ = 2.8, 1C, NCH_3), 64.37 (t, $^3J_{PC}$ = 2.8, 1C, C_1, cHx), 68.48 (t, $^1J_{PC}$ = -62.7, 1C, CHP_2), 128.04 (t, $^2J_{PC}$ = 5.9, 4C, C_{ortho}, (I), P-C_6H_5), 128.17 (t, $^2J_{PC}$ = 5.9, 4C, C_{ortho}, (II), P-C_6H_5), 131.42 (br, 4C, C_{para}, P-C_6H_5), 131.85 (t, $^3J_{PC}$ = 5.0, 4C, C_{meta}, (I), P-C_6H_5) 131.92 (t, $^3J_{PC}$ = 5.0, 4C, C_{meta}, (II), P-C_6H_5), 133.03 (m, *N* = 93.0, 2C, C_{ipso}, (I), P-C_6H_5), 133.31 (m, *N* = 97.4, 2C, C_{ipso}, (II), P-C_6H_5); $[M-H]^-_{found}$ = 526.2080, $C_{32}H_{34}NO_2P_2$ requires 526.2070.

(*N*-Benzyl-*N*-methylaminomethylene)bis(diphenylphosphine oxide) (2e)

Yield: 64% (0.17 g) of compound **2e** as white crystals; Mp: 208-209 °C; ^{31}P NMR ($CDCl_3$) δ 30.04; ^{1}H NMR ($CDCl_3$) δ 2.393 (s, 3 H, NCH_3), 4.156 (s, 2 H, CH_2), 4.864 (t, $^2J_{PH}$ = -16.9 Hz, 1 H, C$H$$P_2$), 6.81-6.89 (m, 2 H, H_{ortho}, C-C_6H_5), 7.09-7.21 (m, 3 H, H_{meta}, and H_{para}, C-C_6H_5), 7.24-7.46 (m, 12 H, H_{ortho} and H_{para}, P-C_6H_5), 7.68-7.80 (m, 4 H, H_{meta}, (I), P-C_6H_5),

7.87-7.98 (m, 4 H, H_{meta}, (II), P-C_6H_5); ^{13}C NMR ($CDCl_3$) δ 41.28 (s, 1C, NCH_3), 62.20 (m, br, 1C, CH_2N), 68.67 (t, $^1J_{PC}$ = -60.3, 1C, CHP$_2$), 126.99 (s, 1C, C_{para}, C-C_6H_5), 128.04 (s, 2C, C_{ortho}, C-C_6H_5), 128.22 (t, $^2J_{PC}$ = 4.3, 4C, C_{ortho}, (I), P-C_6H_5), 128.33 (t, $^2J_{PC}$ = 4.3, 4C, C_{ortho}, (II), P-C_6H_5), 129.04 (s, 2C, C_{meta}, C-C_6H_5), 131.56 (br, 4C, C_{para}, P-C_6H_5), 131.74 (t, $^3J_{PC}$ = 4.9, 4C, C_{meta}, (I), P-C_6H_5), 131.80 (t, $^3J_{PC}$ = 4.9, 4C, C_{meta}, (II), P-C_6H_5), 132.80 (m, N = 93.1, 2C, C_{ipso}, (I), P-C_6H_5), 133.35 (m, N = 98.0, 2C, C_{ipso}, (II), P-C_6H_5), 138.6 (s, 1C, C_{ipso}, C-C_6H_5); $[M+H]^+_{found}$ = 536.1908, $C_{33}H_{32}NO_2P_2$ requires 536.1903.

(*N*-Methylanilinomethylene)bis(diphenylphosphine oxide) (2f)

Yield: 60% (0.16 g) of compound **2f** as white crystals; Mp: 196-197 °C; ^{31}P NMR ($CDCl_3$) δ 28.69; ^{1}H NMR ($CDCl_3$) δ 2.752 (s, 3 H, NCH_3), 5.763 (t, $^2J_{PH}$ = -14.9 Hz, 1 H, CHP$_2$), 6.555 (d, $^3J_{HH}$ = 8.3, 2 H, H_{ortho}, N-C_6H_5), 6.706 (t, $^3J_{HH}$ = 7.2, 1 H, H_{para}, N-C_6H_5), 7.114 (t, $^3J_{HH}$ = 8.0, 2 H, H_{meta}, N-C_6H_5), 7.15-7.44 (m, 12 H, H_{para} and H_{ortho}, P-C_6H_5), 7.68-7.80 (m, 4 H, H_{meta}, (I), P-C_6H_5), 7.81-7.92 (m, 4 H, H_{meta}, (II), P-C_6H_5); ^{13}C NMR ($CDCl_3$) δ 36.94 (s, 1C, NCH_3), 64.94 (t, $^1J_{PC}$ = -63.0, 1C, CHP$_2$), 112.78 (s, 2C, C_{ortho}, C-C_6H_5), 118.01 (s, 1C, C_{para}, C-C_6H_5), 128.17 (t, $^3J_{PC}$ = 5.9, 4C, C_{ortho}, (I), P-C_6H_5), 128.40 (t, $^3J_{PC}$ = 5.9, 4C, C_{ortho}, (II), P-C_6H_5), 129.10 (s, 2C, C_{meta}, C-C_6H_5), 131.54 (t, $^3J_{PC}$ = 5.0, 4C, C_{meta}, (I), P-C_6H_5), 131.60 (t, $^3J_{PC}$ = 4.8, 4C, C_{meta}, (II), P-C_6H_5), 131.73 (t, $^4J_{PC}$ = 1.4, 2C, C_{para}, (I), P-C_6H_5), 131.88 (t, $^4J_{PC}$ = 1.2, 2C, C_{para}, (II), P-C_6H_5), 131.79 (m, N = 94.9, 2C, C_{ipso}, (I), P-C_6H_5), 131.99 (m, N = 100.2, 2C, C_{ipso}, (II), P-C_6H_5), 149.3 (t, $^3J_{PC}$ = 1.2, 1C, C_{ipso}, C-C_6H_5); $[M+H]^+_{found}$ = 522.1749, $C_{32}H_{30}NO_2P_2$ requires 522.1746.

(*N*-Morpholinomethylene)bis(diphenylphosphine oxide) (2g)

Yield: 85% (0.21 g) of compound **2g** as white crystals; Mp: 258-259 °C; Mp [S2]: 172-174 °C; ^{31}P NMR ($CDCl_3$) δ 28.13; δ[S2] ($CDCl_3$) 28.3; ^{1}H NMR ($CDCl_3$) δ 2.86-2.96 (m, br, 4 H, NCH_2), 3.16-3.24 (m, 4 H, OCH_2), 4.610 (t, $^2J_{PH}$ = -16.8 Hz, 1 H, CHP$_2$), 7.26-7.49 (m, 12 H, H_{para} and H_{ortho}, P-C_6H_5), 7.74-7.84 (m, 4 H, H_{meta}, (I), P-C_6H_5), 7.96-8.06 (m, 4 H, H_{meta}, (II), P-C_6H_5); ^{13}C NMR ($CDCl_3$) δ 52.91 (t, br, $^3J_{PC}$ = 3.9, 2C, OCH_2CH_2N), 67.32 (s, 2C, OCH_2CH_2N), 69.62 (t, $^1J_{PC}$ = -61.7, 1C, CHP$_2$), 128.23 (t, $^2J_{PC}$ = 6.0, 4C, C_{ortho}, (I), P-C_6H_5), 128.41 (t, $^2J_{PC}$ = 5.9, 4C, C_{ortho}, (II), P-C_6H_5), 131.55-131.80 (m, 12C, C_{meta} and C_{para}, P-C_6H_5), 132.79 (m, N = 95.2, 2C, C_{ipso}, (I), P-C_6H_5), 133.14 (m, N = 98.3, 2C, C_{ipso}, (II), P-C_6H_5); for the not fully correct NMR data reported see [S2]; $[M+H]^+_{found}$ = 502.1694, $C_{29}H_{30}NO_3P_2$ requires 502.1695.

General procedure for the synthesis of (aminomethylene)bisphosphonates

A mixture of 0.50 mmol amine (*n*-butylamine: 0.05 ml, cyclohexylamine: 0.06 ml, aniline: 0.05 ml), 0.50 mmol trialkyl orthoformate (trimethyl orthoformate: 0.05 ml, triethyl orthoformate: 0.08 ml), and dialkyl phosphite [1.00 mmol (dimethyl phosphite: 0.09 ml, diethyl phosphite: 0.13 ml, dibutyl phosphite 0.20 ml) or 1.50 mmol (diethyl phosphite: 0.19 ml) or 1.75 mmol (dimethyl phosphite: 0.16 ml, diethyl phosphite: 0.23 ml) or 3.00 mmol (dimethyl phosphite: 0.28 ml, dibutyl phosphite 0.59 ml) or 5.00 mmol (dimethyl phosphite: 0.46 ml, dibutyl phosphite 0.98 ml) or 7.50 mmol (dimethyl phosphite: 0.69 ml, dibutyl phosphite 1.46 ml) or 10.00 mmol (dimethyl phosphite: 0.92 ml)] was heated at 110–150 °C in a closed vial in a CEM Discover Microwave reactor equipped with a pressure controller applying 15–40 W for the appropriate time. The reaction mixture so obtained was analyzed by GC and HPLC. The crude products **3a–c** and **7a** were purified on silica gel with dichloromethane/methanol (99:1) as the eluent. The following products were thus prepared:

Tetraethyl (butylaminomethylene)bisphosphonate (3a)

Yield: 61% (0.11 g) of compound **3a** as colorless oil; ^{31}P NMR ($CDCl_3$) δ 19.87; ^{1}H NMR ($CDCl_3$) δ 0.905 (t, $^3J_{HH}$ = 7.2, 3 H, CH_3CH$_2$CH$_2$), 1.350 (t, $^3J_{HH}$ = 7.1, 12 H, OCH$_2$CH_3), 1.31-1.49 (m, 4 H, CH_2CH_2), 1.892 (s, br, 1 H, NH), 2.827 (t, $^3J_{HH}$ = 7.1, 2 H, CH_2N), 3.252 (t, $^2J_{PH}$ = - 21.7, 1 H, C*H*P$_2$), 4.15-4.27 (m, 8 H, OCH_2CH$_3$); ^{13}C NMR ($CDCl_3$) δ 13.92 (s, 1C, *C*H$_3$CH$_2$CH$_2$), 16.46 (t, $^3J_{PC}$ = 3.0, 2C, (I), OCH$_2$*C*H$_3$), 16.51 (t, $^3J_{PC}$ = 3.0, 2C, (II), OCH$_2$*C*H$_3$), 20.17 (s, 1C, CH$_3$*C*H$_2$CH$_2$), 32.18 (s, 1C, CH$_3$CH$_2$*C*H$_2$), 50.32 (t, $^3J_{PC}$ = 5.9, 1C, *C*H$_2$N), 54.43 (t, $^1J_{PC}$ = -145.5, 1C, *C*HP$_2$), 62.89 (t, $^2J_{PC}$ = 3.4, 2C, (I), O*C*H$_2$CH$_3$), 63.29 (t, $^2J_{PC}$ = 3.5, 2C, (II), O*C*H$_2$CH$_3$); $[M+H]^+_{found}$ = 360.1699, $C_{13}H_{32}NO_6P_2$ requires 360.1699.

Tetraethyl (cyclohexylaminomethylene)bisphosphonate (3b)

Yield: 68% (0.13 g) of compound **3b** as colorless oil; ^{31}P NMR ($CDCl_3$) δ 20.05; ^{1}H NMR ($CDCl_3$) δ 0.99-1.26 (m, 5 H, c-C_6H_{11}), 1.347 (t, $^3J_{HH}$ = 7.1, 12 H, OCH$_2$CH_3), 1.56-1.63 (m, 1 H, c-C_6H_{11}), 1.69-1.76 (m, 2 H, c-C_6H_{11}), 1.81-1.87 (m, 2 H, c-C_6H_{11}), 1.88-2.06 (m, br, 1 H, NH), 2.76 (tt, $^3J_{HH}$ = 10.3, $^3J_{HH}$ = 3.7, 1 H, CHN, c-C_6H_{11}), 3.428 (t, $^2J_{PH}$ = -22.0, 1 H, C*H*P$_2$), 4.14-4.28 (m, 8 H, OCH_2CH$_3$); δ[S3] ($CDCl_3$) 0.88–2.00 (m, 11 H, CH$_2$, NH), 1.32 (t, *J* = 7, 12 H, CH$_3$), 2.60-2.96 (m, 1 H, CH), 3.42 (t, *J* = 22, 1 H, CH), 4.00-4.42 (m, 8 H, CH$_2$); ^{13}C NMR ($CDCl_3$) δ 16.45 (t, $^3J_{PC}$ = 3.0, 2C, (I), OCH$_2$*C*H$_3$), 16.52 (t, $^3J_{PC}$ = 3.0, 2C, (II), OCH$_2$*C*H$_3$), 24.70 (s, 2C, cHx), 26.01 (s, 1C, cHx), 33.14 (s, 2C, cHx), 51.08 (t, $^1J_{PC}$ = -145.8,

1C, *C*HP$_2$), 56.25 (t, $^3J_{PC}$ = 5.7, 1C, C_1, cHx), 62.81 (t, $^2J_{PC}$ = 3.6, 2C, (I), O*C*H$_2$CH$_3$), 63.38 (t, $^2J_{PC}$ = 3.2, 2C, (II), O*C*H$_2$CH$_3$); $[M+H]^+_{found}$ = 386.1860, $C_{15}H_{34}NO_6P_2$ requires 386.1856.

Tetraethyl (phenylaminomethylene)bisphosphonate (3c)

Yield: 82% (0.16 g) of compound **3c** as pale yellow crystals; Mp: 90-91 °C; Mp [S4]: 74 °C; Mp [S5]: 89 °C; ^{31}P NMR (CDCl$_3$) δ 17.71; δ[S4] (CDCl$_3$) 17.3; ^{1}H NMR (CDCl$_3$) δ 1.245 (t, $^3J_{HH}$ = 6.8, 6 H, (I), C-C*H*$_3$), 1.290 (t, $^3J_{HH}$ = 6.8, 6 H, (II), C-C*H*$_3$), 4.05-4.30 (m, 10 H, N*H*, NC*H* and OC*H*$_2$), 6.697 (d, $^3J_{HH}$ = 8.0, 2 H, H_{ortho}, N-C$_6$H$_5$), 6.774 (t, $^3J_{HH}$ = 7.4, 1 H, H_{para}, N-C$_6$H$_5$), 7.185 (t, $^3J_{HH}$ = 7.8, 2 H, H_{meta}, N-C$_6$H$_5$); ^{1}H NMR (C$_6$D$_6$) δ 1.010 (t, $^3J_{HH}$ = 7.1, 6 H, (I), C-C*H*$_3$), 1.068 (t, $^3J_{HH}$ = 7.1, 6 H, (II), C-C*H*$_3$), 4.03-4.24 (m, 8 H, OC*H*$_2$), 4.347 (dt, $^3J_{HH}$ = 10.1, $^2J_{PH}$ = -21.7, 1 H, NC*H*), 4.55-4.64 (m, br, 1 H, N*H*), 6.519 (d, $^3J_{HH}$ = 8.0, 2 H, H_{ortho}, N-C$_6$H$_5$), 6.679 (t, $^3J_{HH}$ = 7.3, 1 H, H_{para}, N-C$_6$H$_5$), 7.037 (t, $^3J_{HH}$ = 7.9, 2 H, H_{meta}, N-C$_6$H$_5$); ^{13}C NMR (CDCl$_3$) δ 16.35 (t, $^3J_{PC}$ = 3.1, 2C, (I), OCH$_2$*C*H$_3$), 16.43 (t, $^3J_{PC}$ = 2.9, 2C, (II), OCH$_2$*C*H$_3$), 50.50 (t, $^1J_{PC}$ = -147.3, 1C, *C*HP$_2$), 63.33 (t, $^2J_{PC}$ = 3.4, 2C, (I), O*C*H$_2$CH$_3$), 63.76 (t, $^2J_{PC}$ = 3.5, 2C, (II), O*C*H$_2$CH$_3$), 113.82 (s, 2C, C_{ortho}, C-C$_6$H$_5$), 119.07 (s, 1C, C_{para}, C-C$_6$H$_5$), 129.27 (s, 2C, C_{meta}, C-C$_6$H$_5$), 146.24 (t, $^3J_{PC}$ = 4.2, 1C, C_{ipso}, C-C$_6$H$_5$); for the not fully correct NMR data reported see [S4]; $[M+H]^+_{found}$ = 380.1382, $C_{15}H_{28}NO_6P_2$ requires 380.1386.

Tetramethyl (phenylaminomethylene)bisphosphonate (7a)

Yield: 63% (0.10 g) of compound **7a** as pale yellow crystals; Mp: 168-169 °C; Mp [S5]: 170 °C; ^{31}P NMR (CDCl$_3$) δ 19.99; ^{1}H NMR (CDCl$_3$) δ 3.797 (t, $^3J_{PH}$ = 10.7, 6 H, (I), OC*H*$_3$), 3.816 (t, $^3J_{PH}$ = 10.7, 6 H, (II), OC*H*$_3$), 4.156 (m, br, 1 H, N*H*), 4.253 (dt, $^3J_{HH}$ = 8.9, $^2J_{PH}$ = -21.8, 1 H, C*H*P$_2$), 6.704 (d, $^3J_{HH}$ = 7.9, 2 H, H_{ortho}, N-C$_6$H$_5$), 6.804 (t, $^3J_{HH}$ = 7.4, 1 H, H_{para}, N-C$_6$H$_5$), 7.209 (t, $^3J_{HH}$ = 7.9, 2 H, H_{meta}, N-C$_6$H$_5$); ^{13}C NMR (CDCl$_3$) δ 50.04 (t, $^1J_{PC}$ = -148.0, 1C, *C*HP$_2$), 53.99 (t, $^2J_{PC}$ = 3.3, 2C, (I), POCH$_3$), 54.48 (t, $^2J_{PC}$ = 3.1, 2C, (II), POCH$_3$), 113.84 (s, 2C, H_{ortho}, C-C$_6$H$_5$), 119.53 (s, 1C, H_{para}, C-C$_6$H$_5$), 129.62 (s, 2C, H_{meta}, C-C$_6$H$_5$), 145.95 (t, $^3J_{PC}$ = 4.1, 1C, H_{ipso}, C-C$_6$H$_5$); $[M+H]^+_{found}$ = 324.0760, $C_{11}H_{20}NO_6P_2$ requires 324.0760.

General procedure for the synthesis of (dialkylaminomethylene)bisphosphonates

A mixture of 0.50 mmol amine (diethylamine: 0.05 ml, dibutylamine: 0.08 ml, *N*-butyl-*N*-methylamine: 0.06 ml, *N*-cyclohexyl-*N*-methylamine: 0.065 ml, *N*-benzyl-*N*-methylamine: 0.065 ml, *N*-methylaniline: 0.05 ml, morpholine: 0.04 ml), 0.50 mmol (0.08 ml) of triethyl

orthoformate, and 1.75 mmol (0.23 ml) or 2.25 mmol (0.29 ml) of diethyl phosphite was heated at 125 °C in a closed vial in a CEM Discover Microwave reactor equipped with a pressure controller applying 30–40 W for 1 h. The crude product so obtained was purified on silica gel with dichloromethane/methanol (99:1) as the eluent. After evaporation of the solvent, the products (**8a–g**) were obtained as colorless oils. The following products were thus prepared:

Tetraethyl (diethylaminomethylene)bisphosphonate (8a)

Yield: 86% (0.15 g) of compound **5a** as colorless oil; ^{31}P NMR ($CDCl_3$) δ 19.73; ^{1}H NMR ($CDCl_3$) δ 1.050 (t, $^3J_{HH}$ = 7.1, 6 H, C*H*$_3$CH$_2$N), 1.342 (t, $^3J_{HH}$ = 7.1, 12 H, OCH$_2$C*H*$_3$), 2.900 (q, $^3J_{HH}$ = 7.0, 4 H, CH$_3$C*H*$_2$N), 3.557 (t, $^2J_{PH}$ = -25.3, 1 H, C*H*P$_2$), 4.11-4.29 (m, 8 H, OC*H*$_2$CH$_3$); ^{13}C NMR ($CDCl_3$) δ 14.39 (s, 2C, *C*H$_3$CH$_2$N), 16.47 (t, $^3J_{PC}$ = 1.7, 2C, (I), OCH$_2$*C*H$_3$), 16.52 (t, $^3J_{PC}$ = 1.7, 2C, (II), OCH$_2$*C*H$_3$), 46.97 (t, $^3J_{PC}$ = 4.6, 2C, *C*H$_2$N), 57.04 (t, $^1J_{PC}$ = -142.7, 1C, *C*HP$_2$), 62.40 (t, $^2J_{PC}$ = 3.6, 2C, (I), O*C*H$_2$CH$_3$), 62.92 (t, $^2J_{PC}$ = 3.2, 2C, (II), O*C*H$_2$CH$_3$); $[M+H]^+_{found}$ = 360.1711, $C_{13}H_{32}NO_6P_2$ requires 360.1699.

Tetraethyl (dibutylaminomethylene)bisphosphonate (8b)

Yield: 68 % (0.14 g) of compound **8b** as colorless oil; ^{31}P NMR ($CDCl_3$) δ 20.09; ^{1}H NMR ($CDCl_3$) δ 0.906 (t, $^3J_{HH}$ = 7.3, 6 H, CH$_2$CH$_2$C*H*$_3$), 1.342 (t, $^3J_{HH}$ = 7.1, 12 H, OCH$_2$C*H*$_3$), 1.27-1.36 (m, 4 H, CH$_2$C*H*$_2$CH$_3$), 1.36-1.45 (m, 4 H, C*H*$_2$CH$_2$CH$_3$), 2.821 (t, $^3J_{HH}$ = 7.3, 4 H, NC*H*$_2$), 3.520 (t, $^2J_{PH}$ = -25.4, 1 H, C*H*P$_2$), 4.13-4.26 (m, 8 H, OC*H*$_2$CH$_3$); ^{13}C NMR ($CDCl_3$) δ 14.13 (s, 2C, *C*H$_3$CH$_2$CH$_2$), 16.46 (t, $^3J_{PC}$ = 3.3, 2C, (I), OCH$_2$*C*H$_3$), 16.52 (t, $^3J_{PC}$ = 3.3, 2C, (II), OCH$_2$*C*H$_3$), 20.25 (s, 2C, CH$_3$*C*H$_2$CH$_2$), 31.46 (s, 2C, CH$_3$CH$_2$*C*H$_2$), 53.39 (t, $^3J_{PC}$ = 4.6, 2C, *C*H$_2$N), 57.98 (t, $^1J_{PC}$ = -142.6, 1C, *C*HP$_2$), 62.28 (t, $^2J_{PC}$ = 3.8, 2C, (I), O*C*H$_2$CH$_3$), 62.66 (t, $^2J_{PC}$ = 3.5, 2C, (II), O*C*H$_2$CH$_3$); $[M+H]^+_{found}$ = 416.2318, $C_{17}H_{40}NO_6P_2$ requires 416.2325.

Tetraethyl (*N*-butyl-*N*-methylaminomethylene)bisphosphonate (8c)

Yield: 79% (0.15 g) of compound **8c** as colorless oil; ^{31}P NMR ($CDCl_3$) δ 19.38; ^{1}H NMR ($CDCl_3$) δ 0.908 (t, $^3J_{HH}$ = 7.3, 3 H, CH$_2$CH$_2$C*H*$_3$), 1.349 (t, $^3J_{HH}$ = 7.1, 12 H, OCH$_2$C*H*$_3$), 1.28-1.37 (m, 2 H, CH$_2$C*H*$_2$CH$_3$), 1.39-1.50 (m, 2 H, C*H*$_2$CH$_2$CH$_3$), 2.644 (s, 3 H, NC*H*$_3$), 2.800 (t, $^3J_{HH}$ = 7.4 Hz, 2 H, C*H*$_2$N), 3.468 (t, $^2J_{PH}$ = -25.2, 1 H, C*H*P$_2$), 4.16-4.25 (m, 8 H, OC*H*$_2$CH$_3$); ^{13}C NMR ($CDCl_3$) δ 14.02 (s, 1C, *C*H$_3$CH$_2$CH$_2$), 16.45 (t, $^3J_{PC}$ = 3.3, 2C, (I), OCH$_2$*C*H$_3$), 16.51 (t, $^3J_{PC}$ = 3.3, 2C, (II), OCH$_2$*C*H$_3$), 20.14 (s, 1C, CH$_3$*C*H$_2$CH$_2$), 30.56 (s, 1C, CH$_3$CH$_2$*C*H$_2$), 40.43 (t, $^3J_{PC}$ = 3.8, 1C, N*C*H$_3$), 56.94 (t, $^3J_{PC}$ = 5.3, 1C, *C*H$_2$N), 60.51 (t,

$^{1}J_{PC}$ = -141.6, 1C, CHP_2), 62.43 (t, $^{2}J_{PC}$ = 3.6, 2C, (I), OCH_2CH_3), 62.79 (t, $^{2}J_{PC}$ = 3.3, 2C, (II), OCH_2CH_3); $[M+H]^{+}_{found}$ = 374.1846, $C_{14}H_{34}NO_6P_2$ requires 374.1856.

Tetraethyl (*N*-cyclohexyl-*N*-methylaminomethylene)bisphosphonate (8d)

Yield: 72% (0.14 g) of compound **8d** as colorless oil; ^{31}P NMR ($CDCl_3$) δ 19.95; ^{1}H NMR ($CDCl_3$) δ 1.03-1.30 (m, 5 H, c-C_6H_{11}), 1.344 (t, $^{3}J_{HH}$ = 7.1, 12 H, OCH_2CH_3), 1.54-1.63 (m, 1 H, c-C_6H_{11}), 1.73-1.80 (m, 2 H, c-C_6H_{11}), 1.92-2.00 (m, 2 H, c-C_6H_{11}), 2.692 (s, 3 H, NCH_3), 3.728 (t, $^{2}J_{PH}$ = -25.8, 1 H, CHP_2), 4.15-4.22 (m, 8 H, OCH_2CH_3); ^{13}C NMR ($CDCl_3$) δ 16.46 (t, $^{3}J_{PC}$ = 3.1, 2C, (I), OCH_2CH_3), 16.53 (t, $^{3}J_{PC}$ = 3.2, 2C, (II), OCH_2CH_3), 25.44 (s, 2C, cHx), 25.95 (s, 1C, cHx), 31.08 (s, 2C, cHx), 36.14 (t, $^{3}J_{PC}$ = 4.7, 1C, NCH_3), 57.08 (t, $^{1}J_{PC}$ = -143.9, 1C, CHP_2), 62.33 (t, $^{2}J_{PC}$ = 3.2, 2C, (I), OCH_2CH_3), 62.74 (t, $^{2}J_{PC}$ = 2.8, 2C, (II), OCH_2CH_3) and superimposed with 62.74 (br, C_1, cHx); $[M+H]^{+}_{found}$ = 400.2016, $C_{16}H_{36}NO_6P_2$ requires 400.2012.

Tetraethyl (*N*-benzyl-*N*-methylaminomethylene)bisphosphonate (8e)

Yield: 70% (0.14 g) of compound **8e** as colorless oil; ^{31}P NMR ($CDCl_3$) δ 19.66; δ[S6] ($CDCl_3$) 19.5; ^{1}H NMR ($CDCl_3$) δ 1.347 (t, $^{3}J_{HH}$ = 7.1, 12 H, CH_2CH_3), 2.663 (s, 3 H, NCH_3), 3.488 (t, $^{2}J_{PH}$ = -25.3, 1 H, CHP_2), 3.988 (s, 2H, CH_2N), 4.10-4.27 (m, 8 H, OCH_2CH_3), 7.20-7.42 (m, 5 H, C_6H_5); ^{13}C NMR ($CDCl_3$) δ 16.48 (t, $^{3}J_{PC}$ = 3.1, 2C, (I), OCH_2CH_3), 16.54 (t, $^{3}J_{PC}$ = 3.1, 2C, (II), OCH_2CH_3), 40.78 (t, $^{3}J_{PC}$ = 3.7, 1C, NCH_3), 59.04 (t, $^{1}J_{PC}$ = -141.4, 1C, CHP_2), 60.80 (t, $^{3}J_{PC}$ = 5.3, 1C, NCH_2), 62.39 (t, $^{2}J_{PC}$ = 3.5, 2C, (I), OCH_2CH_3), 62.68 (t, $^{2}J_{PC}$ = 3.2, 2C, (II), OCH_2CH_3), 127.26 (s, 2C, C_{ortho}, C-C_6H_5), 128.21 (s, 1C, C_{para}, C-C_6H_5), 129.33 (s, 2C, C_{meta}, C-C_6H_5), 138.90 (s, 1C, C_{ipso}, C-C_6H_5); $[M+H]^{+}_{found}$ = 408.1697, $C_{17}H_{32}NO_6P_2$ requires 408.1699.

Tetraethyl (methylanilinomethylene)bisphosphonate (8f)

Yield: 65% (0.12 g) of compound **8f** as pale yellow oil; ^{31}P NMR ($CDCl_3$) δ 17.81; ^{1}H NMR ($CDCl_3$) δ 1.266 (t, $^{3}J_{HH}$ = 7.1, 6 H, (I), OCH_2CH_3), 1.272 (t, $^{3}J_{HH}$ = 7.1, 6 H, (II), OCH_2CH_3), 3.171 (s, 3 H, NCH_3), 4.05-4.23 (m, 8 H, OCH_2CH_3), 4.617 (t, $^{2}J_{PH}$ = -25.6, 1 H, CHP_2), 6.791 (t, $^{3}J_{HH}$ = 7.2, 1 H, H_{para}, C_6H_5), 6.859 (d, $^{3}J_{HH}$ = 7.2, 2 H, H_{ortho}, C_6H_5), 7.241 (t, $^{3}J_{HH}$ = 8.0, 2 H, H_{meta}, C_6H_5); ^{13}C NMR ($CDCl_3$) δ 16.36 (t, $^{3}J_{PC}$ = 3.1, 2C, (I), OCH_2CH_3), 16.44 (t, $^{3}J_{PC}$ = 3.0, 2C, (II), OCH_2CH_3), 35.93 (s, 1C, NCH_3), 56.94 (t, $^{1}J_{PC}$ = -146.2, 1C, CHP_2), 63.03 (t, $^{2}J_{PC}$ = 3.0, 2C, (I), OCH_2CH_3), 63.27 (t, $^{2}J_{PC}$ = 2.8, 2C, (II), OCH_2CH_3), 113.83 (s,

2C, C_{ortho}, C-C_6H_5), 118.47 (s, 1C, C_{para}, C-C_6H_5), 129.14 (s, 2C, C_{meta}, C-C_6H_5), 149.82 (t, $^3J_{PC}$ = 3.1, 1C, C_{ipso}, C-C_6H_5); $[M+H]^+_{found}$ = 394.1546, $C_{16}H_{30}NO_6P_2$ requires 394.1543.

Tetraethyl (morpholinomethylene)bisphosphonate (8g)

Yield: 81% (0.15 g) of compound **8g** as colorless oil; ^{31}P NMR ($CDCl_3$) δ 18.19; ^{1}H NMR ($CDCl_3$) δ 1.362 (t, $^3J_{HH}$ = 7.0, 6 H, (I), OCH_2CH_3), 1.365 (t, $^3J_{HH}$ = 7.1, 6 H, (II), OCH_2CH_3), 3.037 (m, 4 H, $O(CH_2CH_2)_2N$), 3.323 (t, $^2J_{PH}$ = -24.7, 1 H, CHP_2), 3.675 (m, 4 H, $O(CH_2CH_2)_2N$), 4.13-4.31 (m, 8 H, OCH_2CH_3); ^{13}C NMR ($CDCl_3$) δ 16.61 (t, $^3J_{PC}$ = 2.9, 2C, (I), OCH_2CH_3), 16.72 (t, $^3J_{PC}$ = 3.6, 2C, (II), OCH_2CH_3), 52.13 (t, $^3J_{PC}$ = 4.7, 2C, OCH_2CH_2N), 61.81 (t, $^1J_{PC}$ = -141.7, 1C, CHP_2), 62.74 (t, $^2J_{PC}$ = 3.5, 2C, (I), OCH_2CH_3), 63.32 (t, $^2J_{PC}$ = 3.2, 2C, (II), OCH_2CH_3), 67.84 (s, 2C, OCH_2CH_2N); $[M+H]^+_{found}$ = 374.1491, $C_{13}H_{30}NO_4P$ requires 374.1492.

^{31}P NMR, ^{1}H NMR and ^{13}C NMR spectra

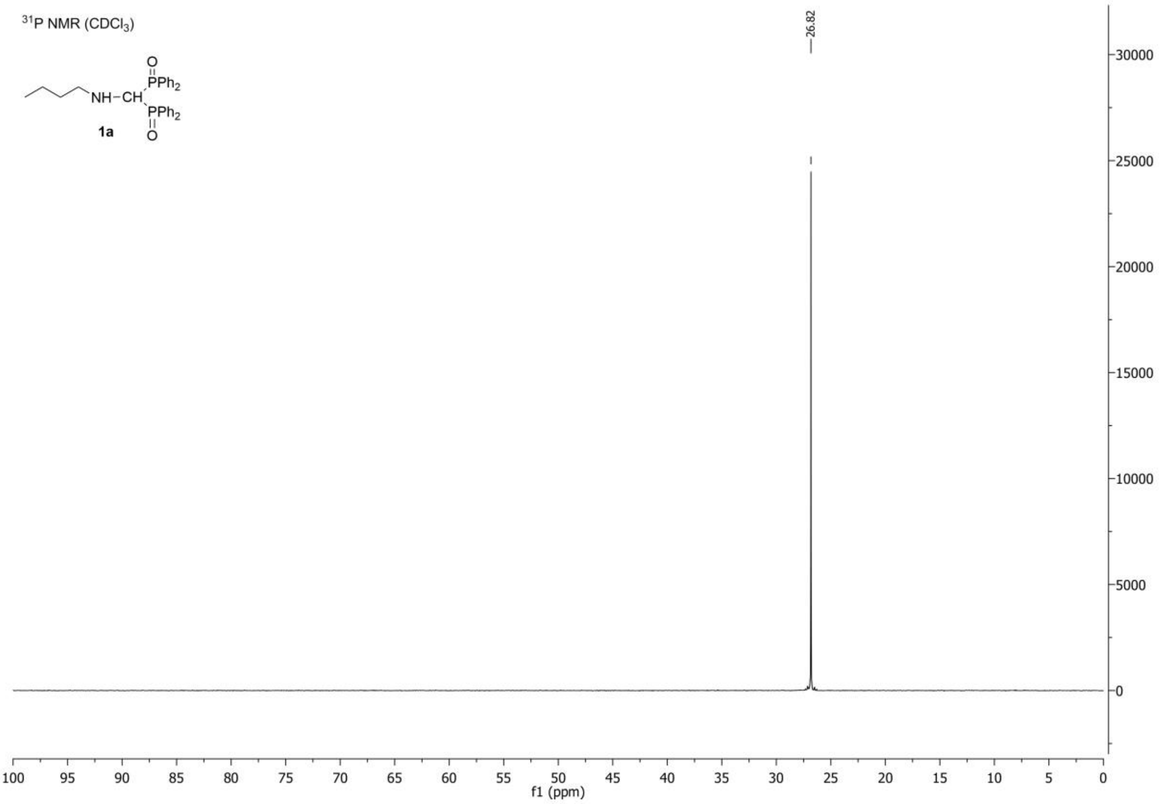

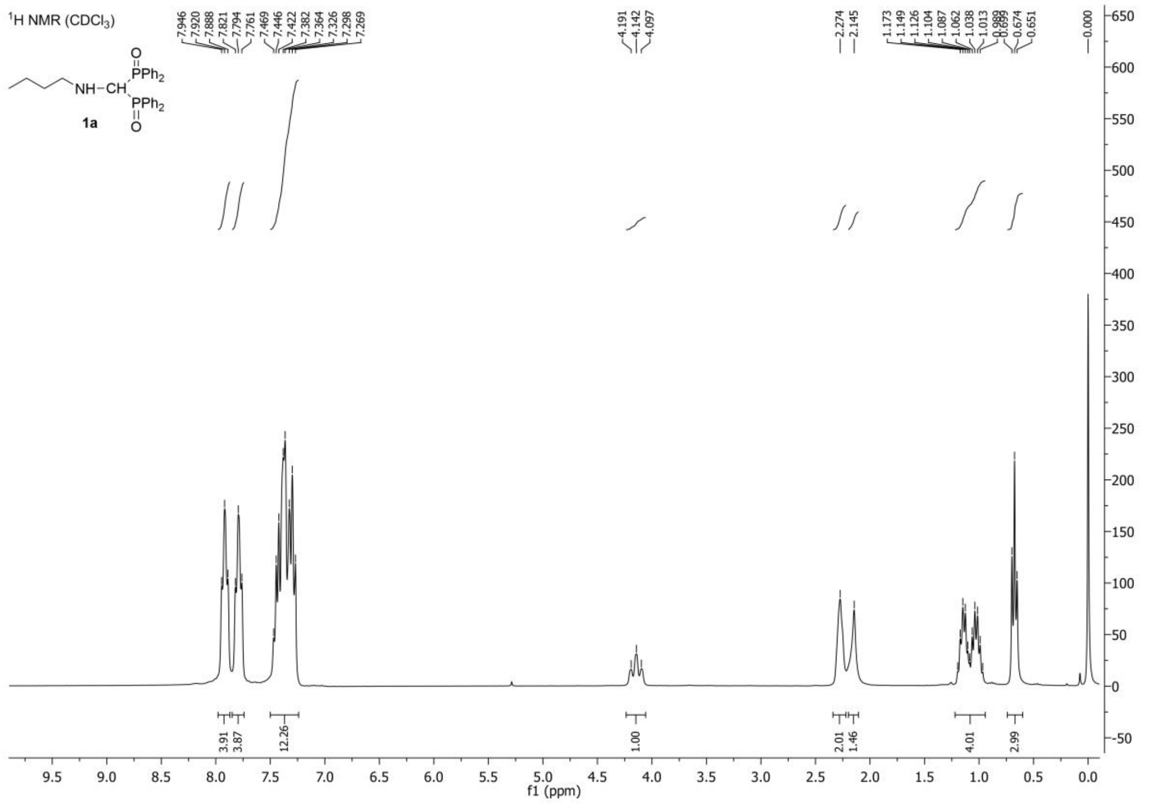

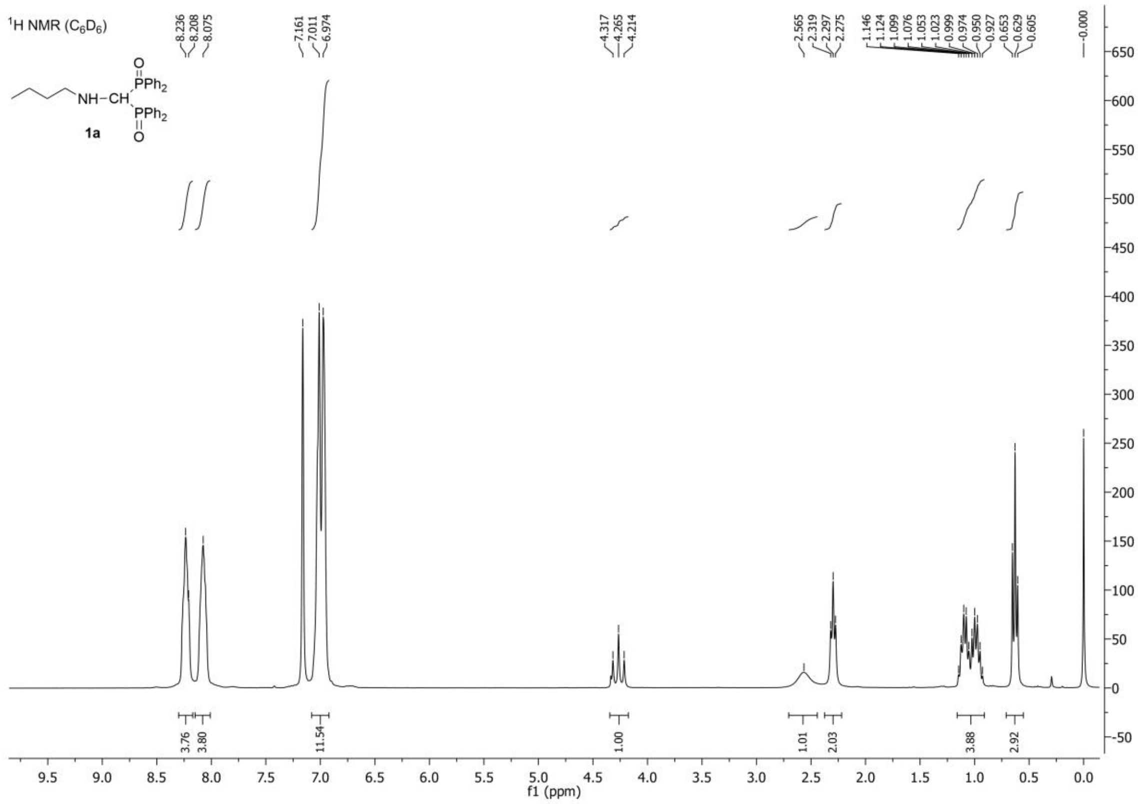
^{1}H NMR (C_6D_6)
NH−CH
PPh2
O
1a
8.236
8.208
8.075
7.161
7.011
6.974
4.317
4.265
4.214
2.565
2.319
2.297
2.275
1.146
1.124
1.099
1.076
1.053
1.023
0.999
0.974
0.950
0.927
0.653
0.629
0.605
0.000
3.76
3.80
11.54
1.00
1.01
2.03
3.88
2.92
f1 (ppm)

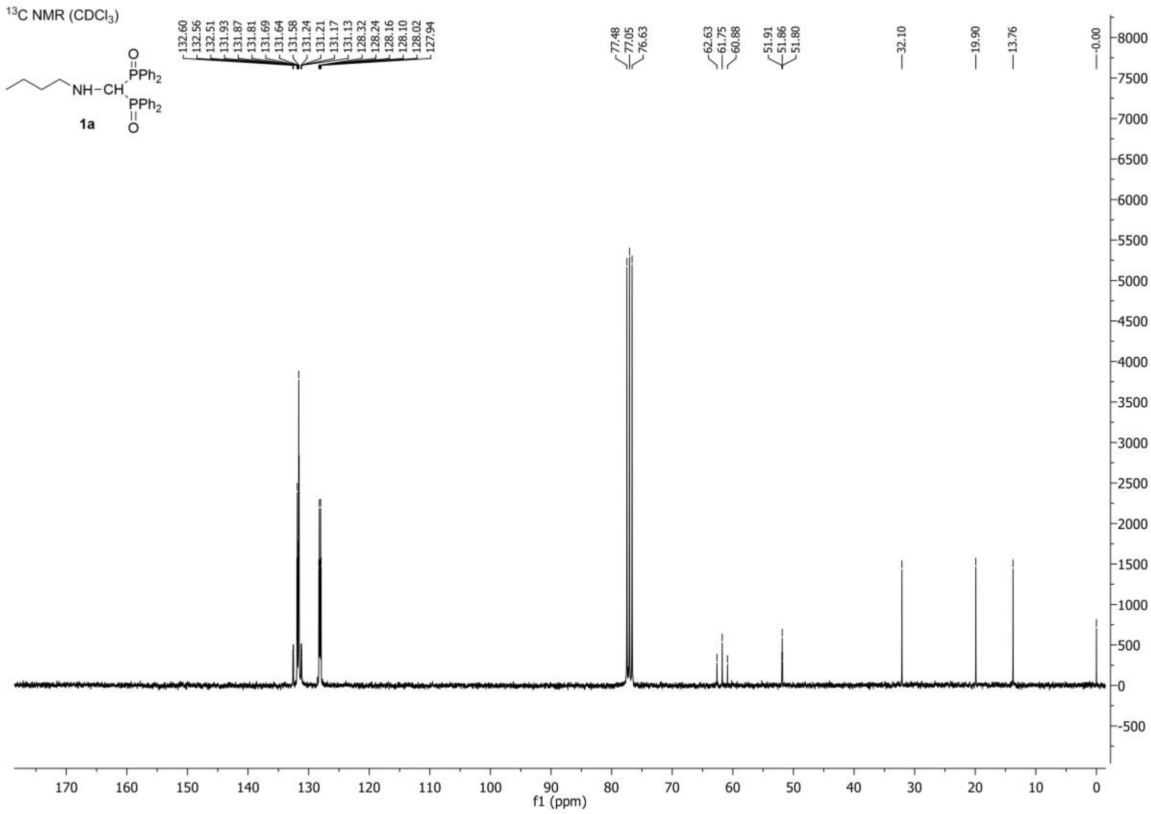
^{13}C NMR ($CDCl_3$)
NH−CH
PPh2
O
1a
132.60
132.56
132.51
131.93
131.87
131.81
131.69
131.64
131.58
131.24
131.21
131.17
131.13
128.32
128.24
128.16
128.10
128.02
127.94
77.48
77.05
76.63
62.63
61.75
60.88
51.91
51.86
51.80
32.10
19.90
13.76
-0.00
f1 (ppm)

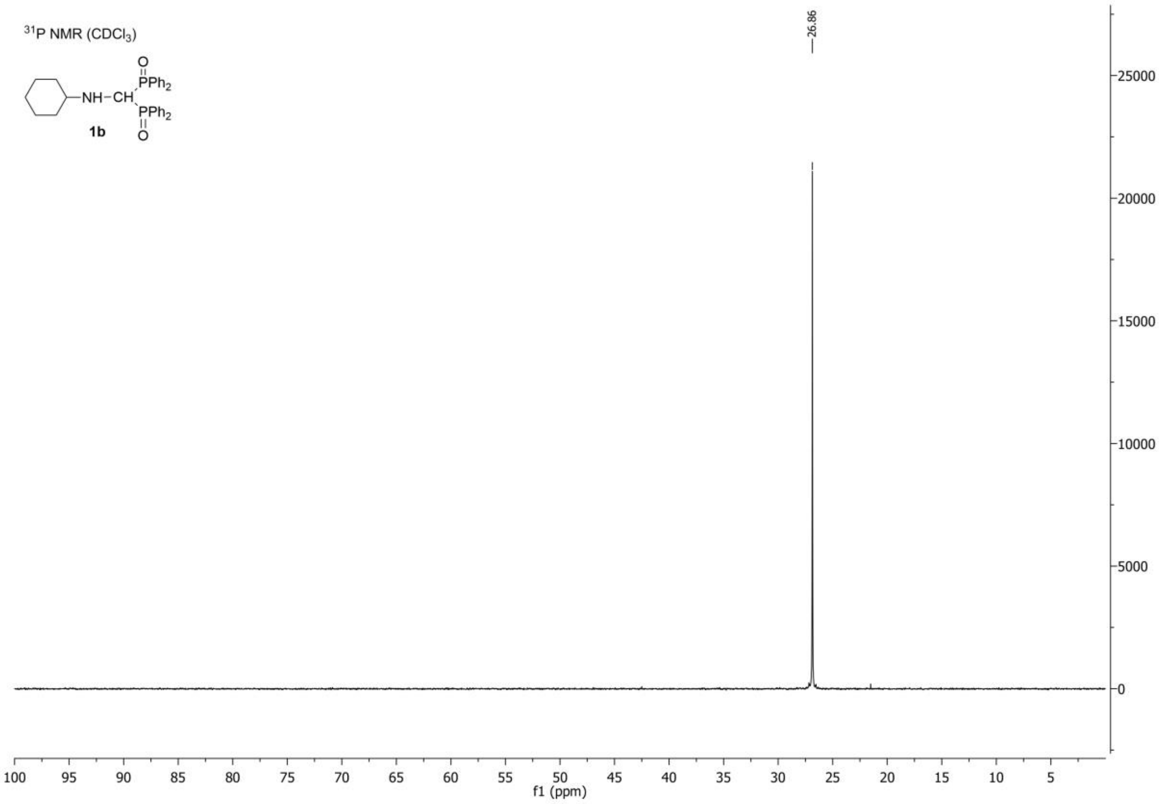

^{31}P NMR ($CDCl_3$)
1b
26.86
f1 (ppm)

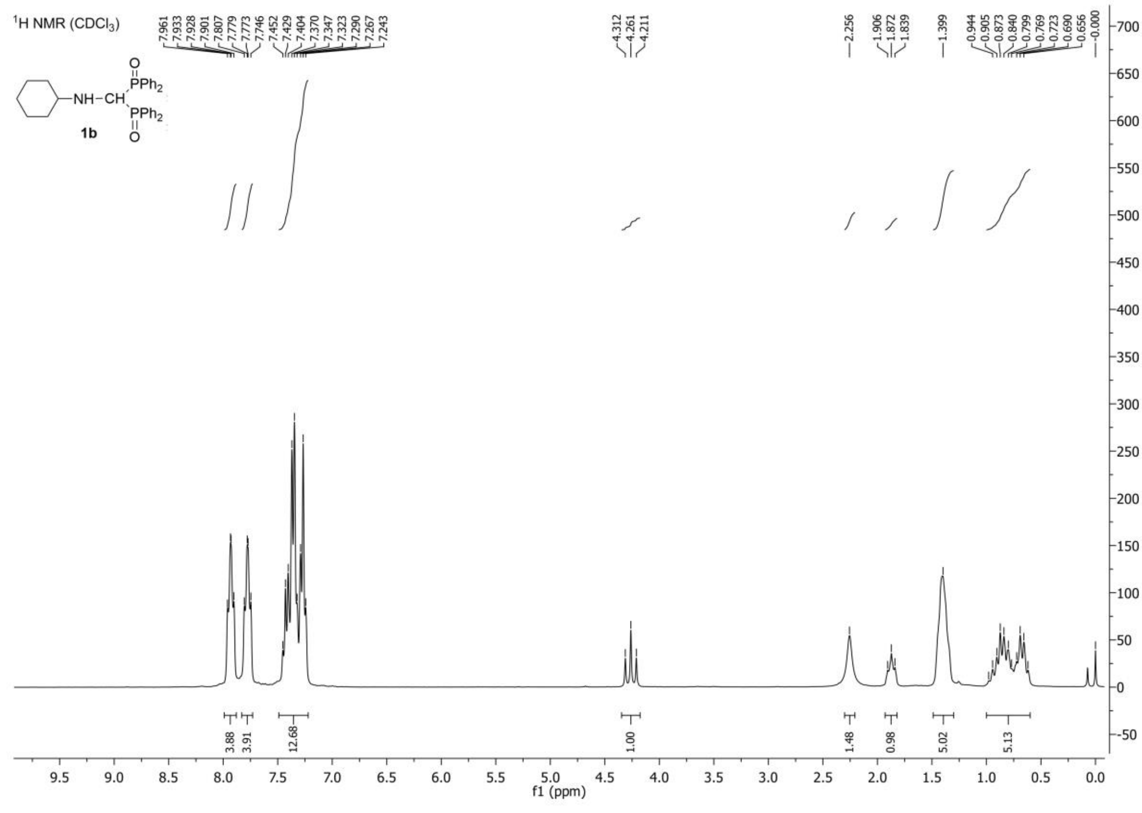

^{1}H NMR ($CDCl_3$)
1b
3.88
3.91
12.68
1.00
1.48
0.98
5.02
5.13
f1 (ppm)

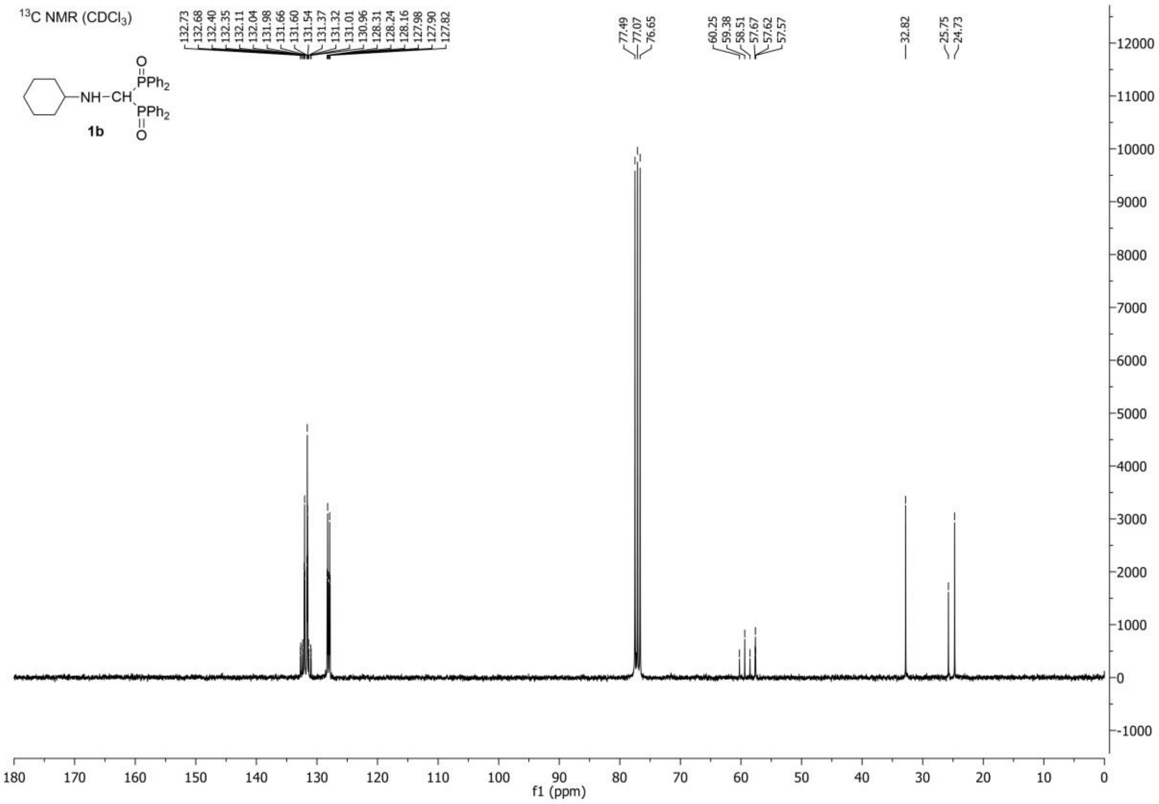
13C NMR (CDCl3)
1b
132.73
132.68
132.40
132.35
132.11
132.04
131.98
131.66
131.60
131.54
131.37
131.32
131.01
130.96
128.31
128.24
128.16
127.98
127.90
127.82
77.49
77.07
76.65
60.25
59.38
58.51
57.67
57.62
57.57
32.82
25.75
24.73
f1 (ppm)

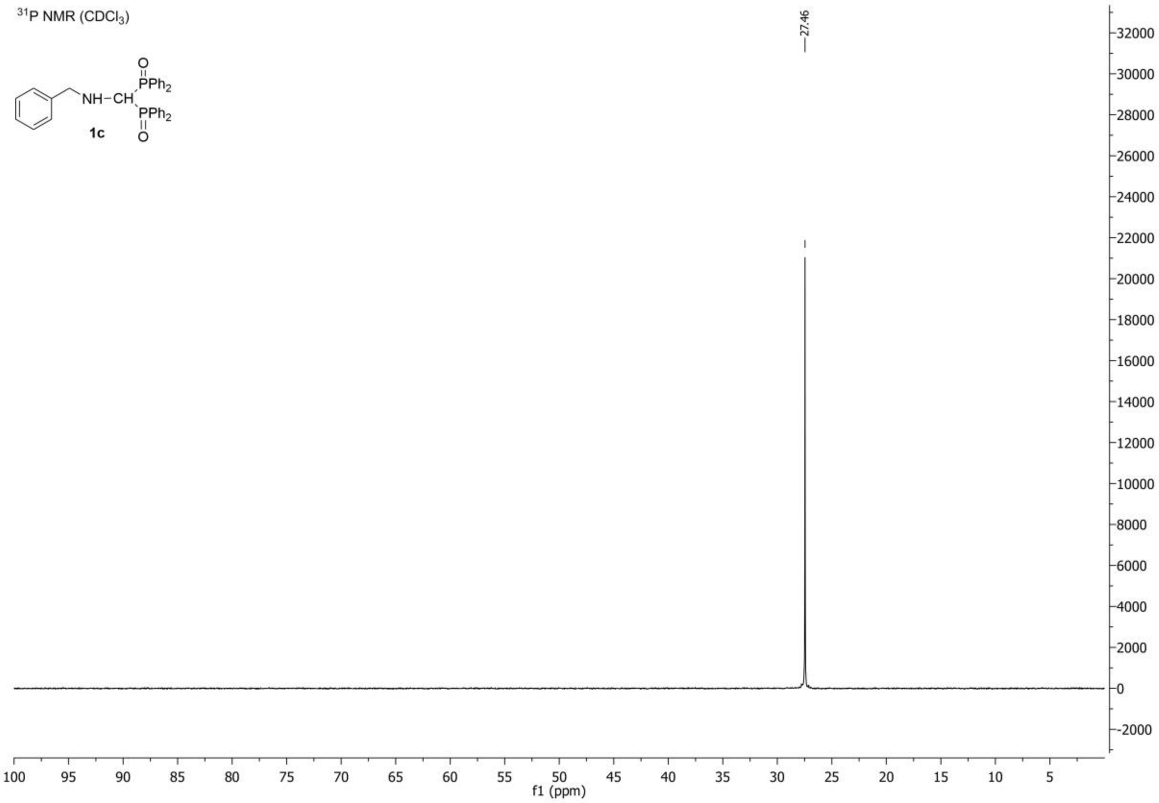
31P NMR (CDCl3)
1c
27.46
f1 (ppm)

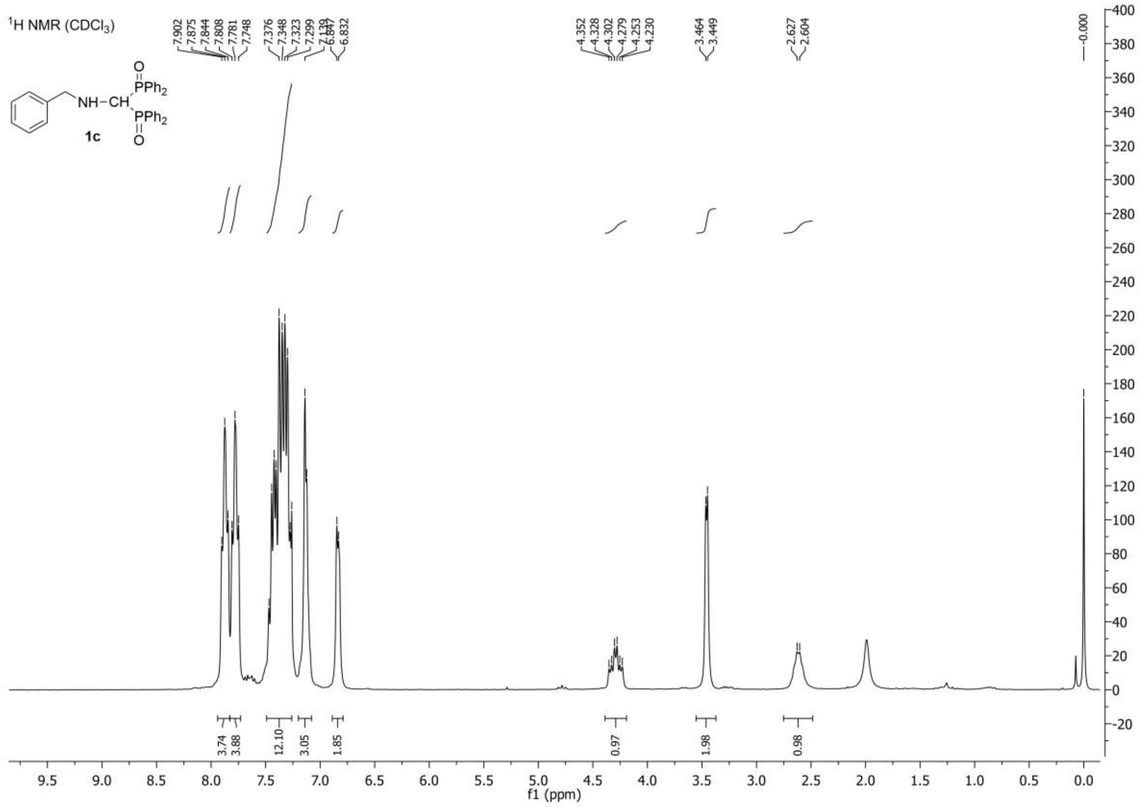

¹H NMR (CDCl₃)
1c
f1 (ppm)

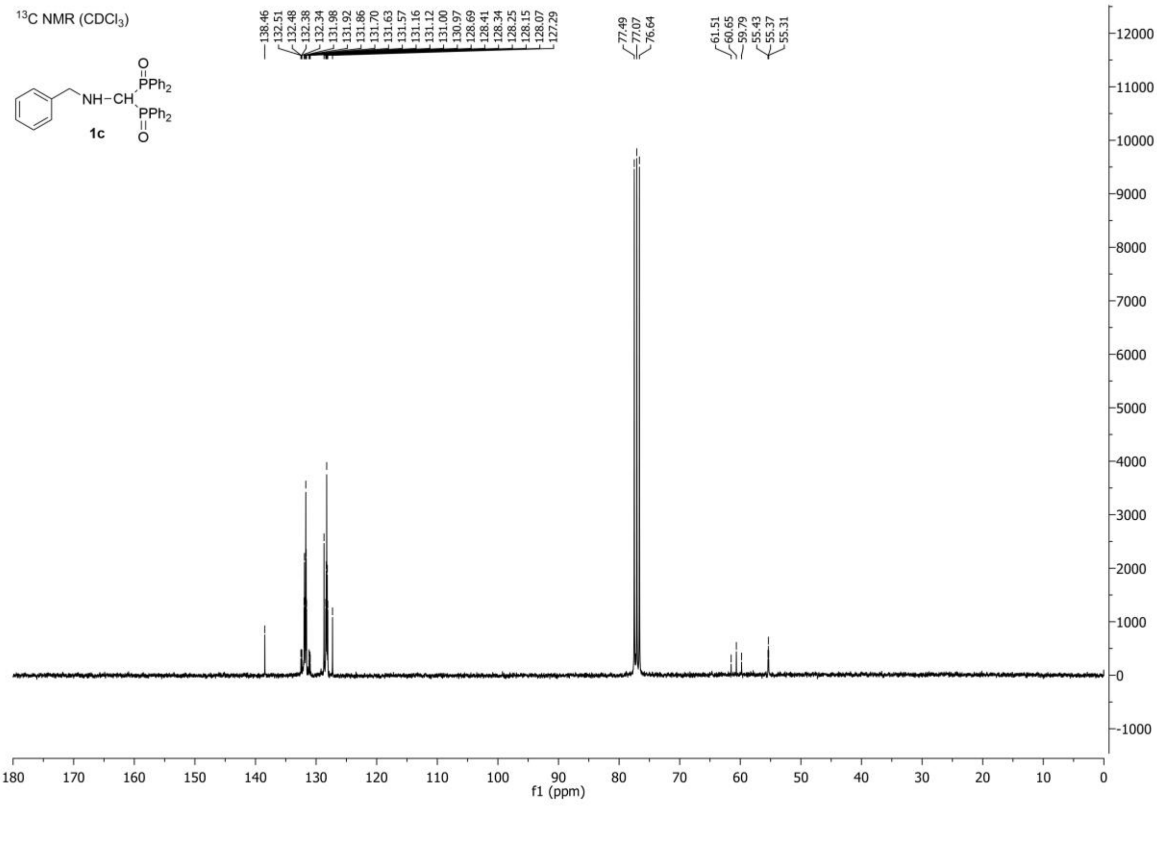

¹³C NMR (CDCl₃)
1c
f1 (ppm)

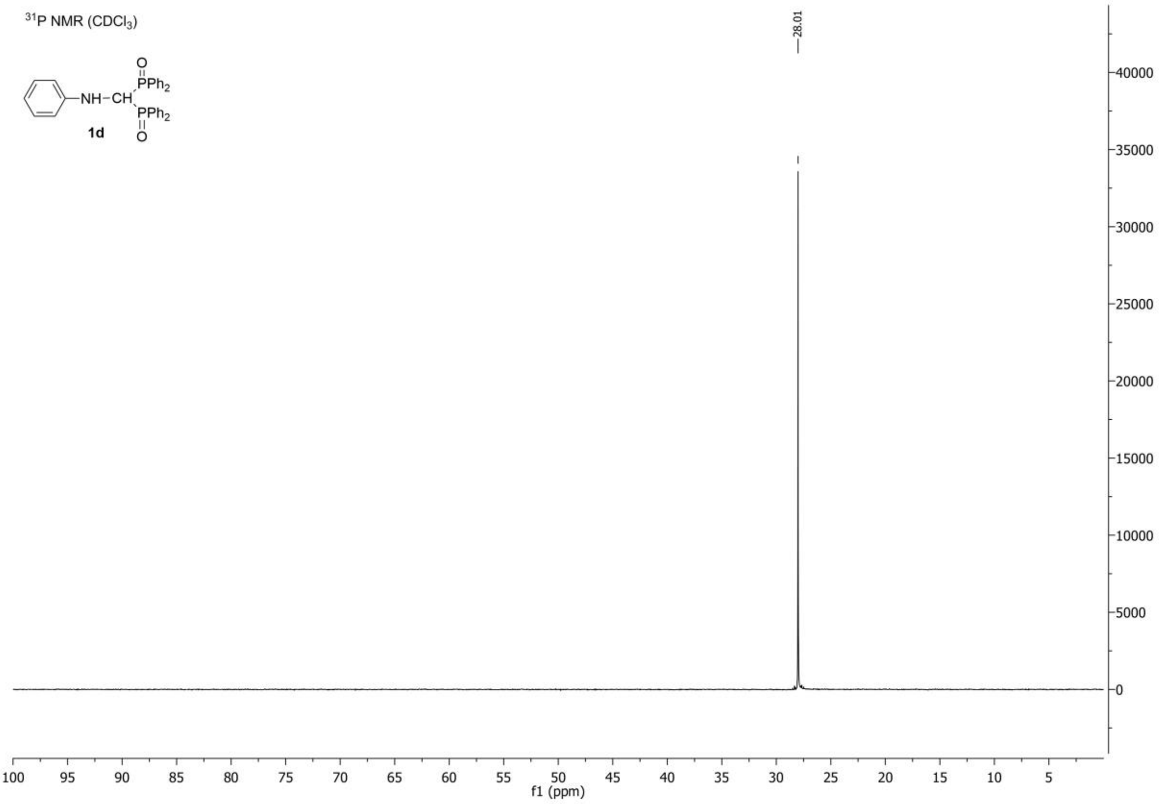
31P NMR (CDCl3)
NH−CH
PPh2
PPh2
1d
28.01
f1 (ppm)

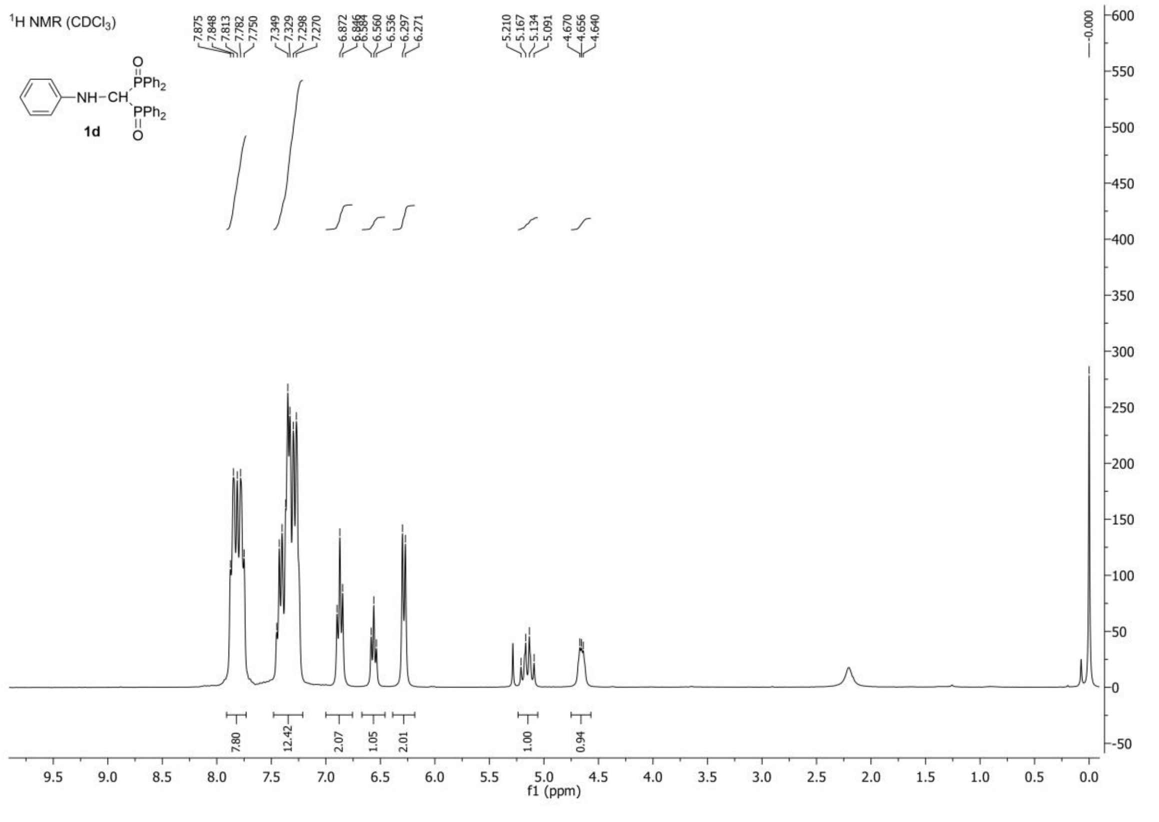
1H NMR (CDCl3)
NH−CH
PPh2
PPh2
1d
7.875
7.848
7.813
7.782
7.750
7.349
7.329
7.298
7.270
6.872
6.560
6.536
6.297
6.271
5.210
5.167
5.134
5.091
4.670
4.656
4.640
0.000
7.80
12.42
2.07
1.05
2.01
1.00
0.94
f1 (ppm)

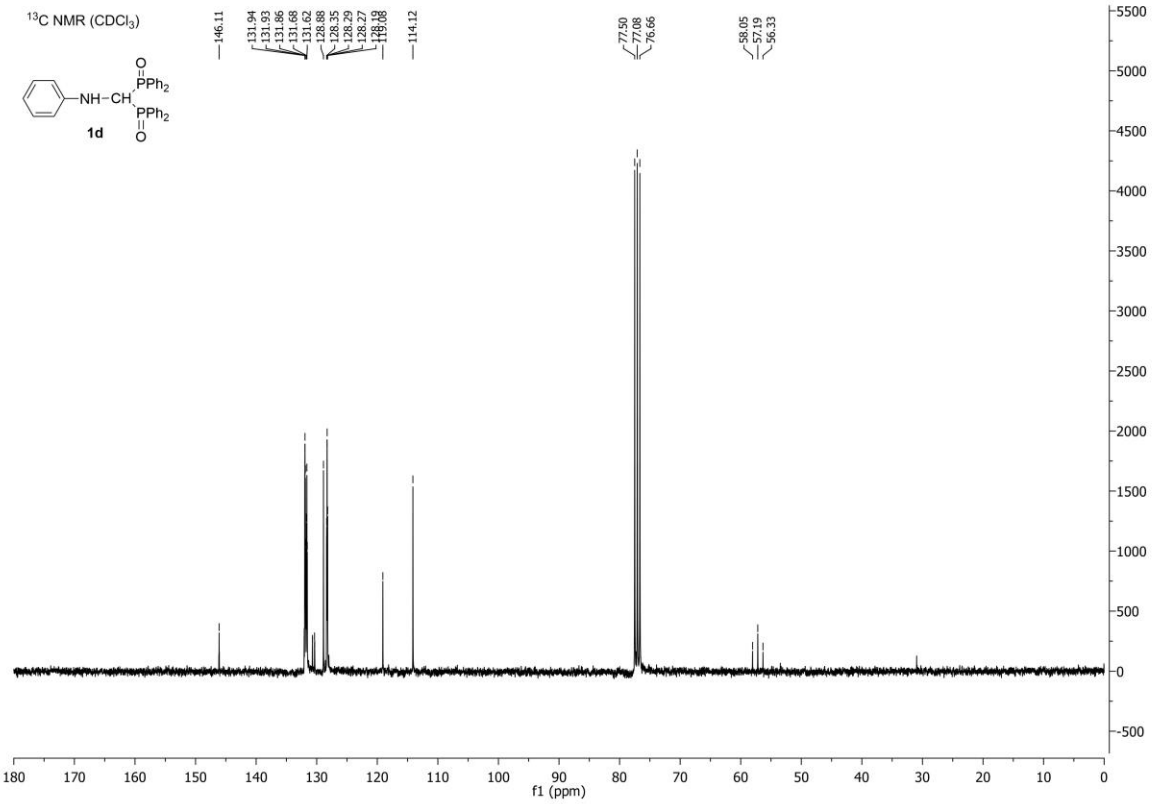

13C NMR (CDCl3)
1d
NH−CH
PPh2
O
146.11
131.94
131.93
131.86
131.68
131.62
128.88
128.35
128.29
128.27
128.19
119.08
114.12
77.50
77.08
76.66
58.05
57.19
56.33
f1 (ppm)

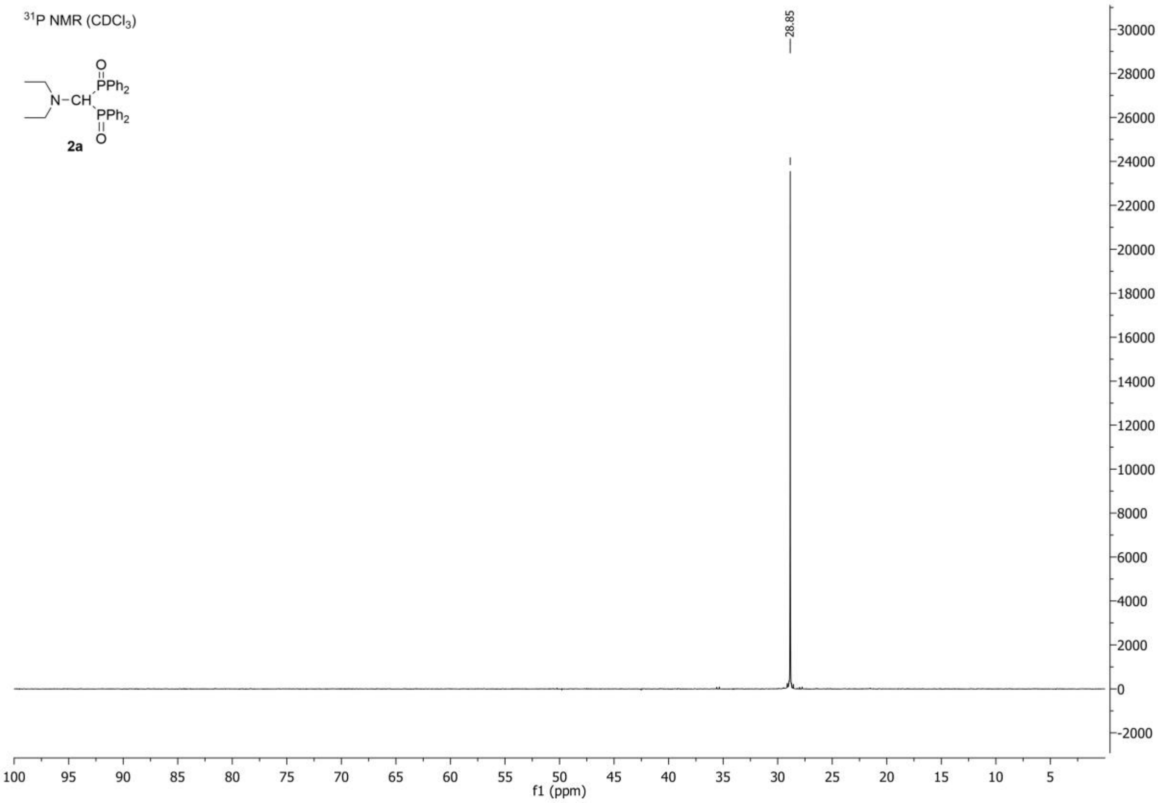

31P NMR (CDCl3)
2a
N−CH
PPh2
O
28.85
f1 (ppm)

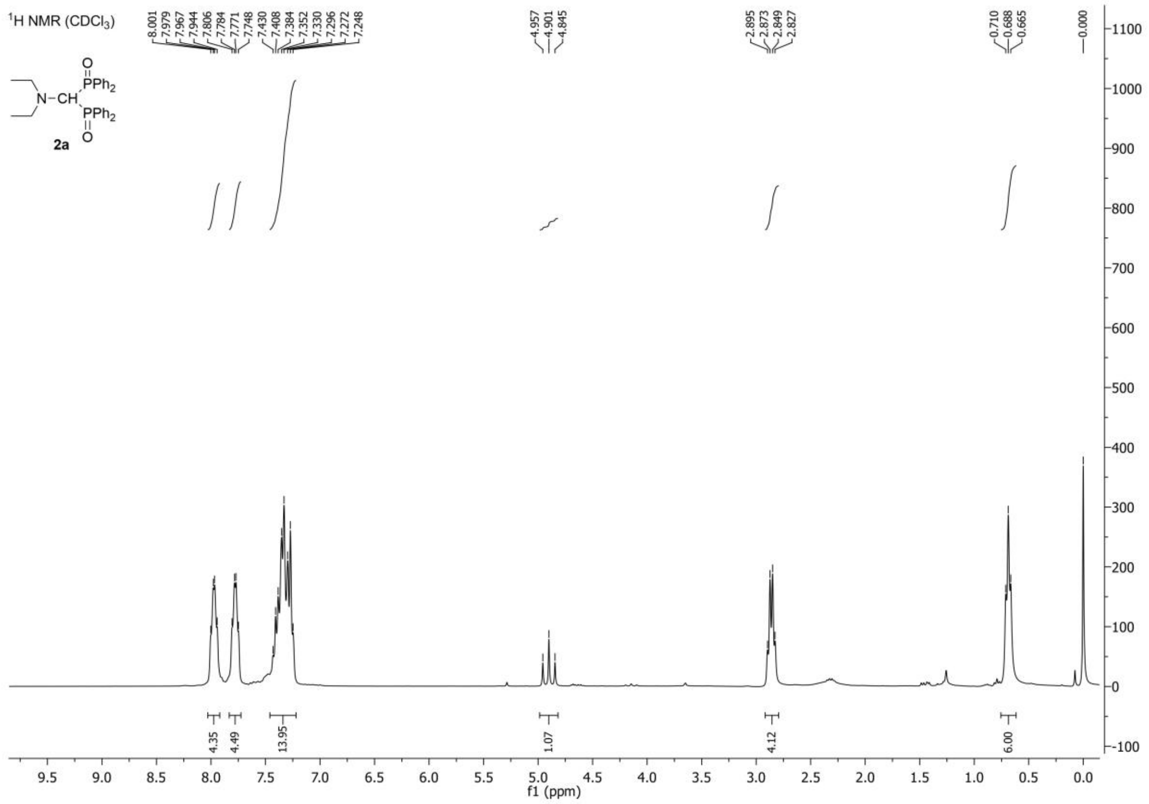
^{1}H NMR (CDCl$_3$)
2a
8.001 7.979 7.967 7.944 7.806 7.784 7.771 7.748 7.430 7.408 7.384 7.352 7.330 7.296 7.272 7.248
4.957 4.901 4.845
2.895 2.873 2.849 2.827
0.710 0.688 0.665
0.000
4.35 4.49 13.95 1.07 4.12 6.00
f1 (ppm)

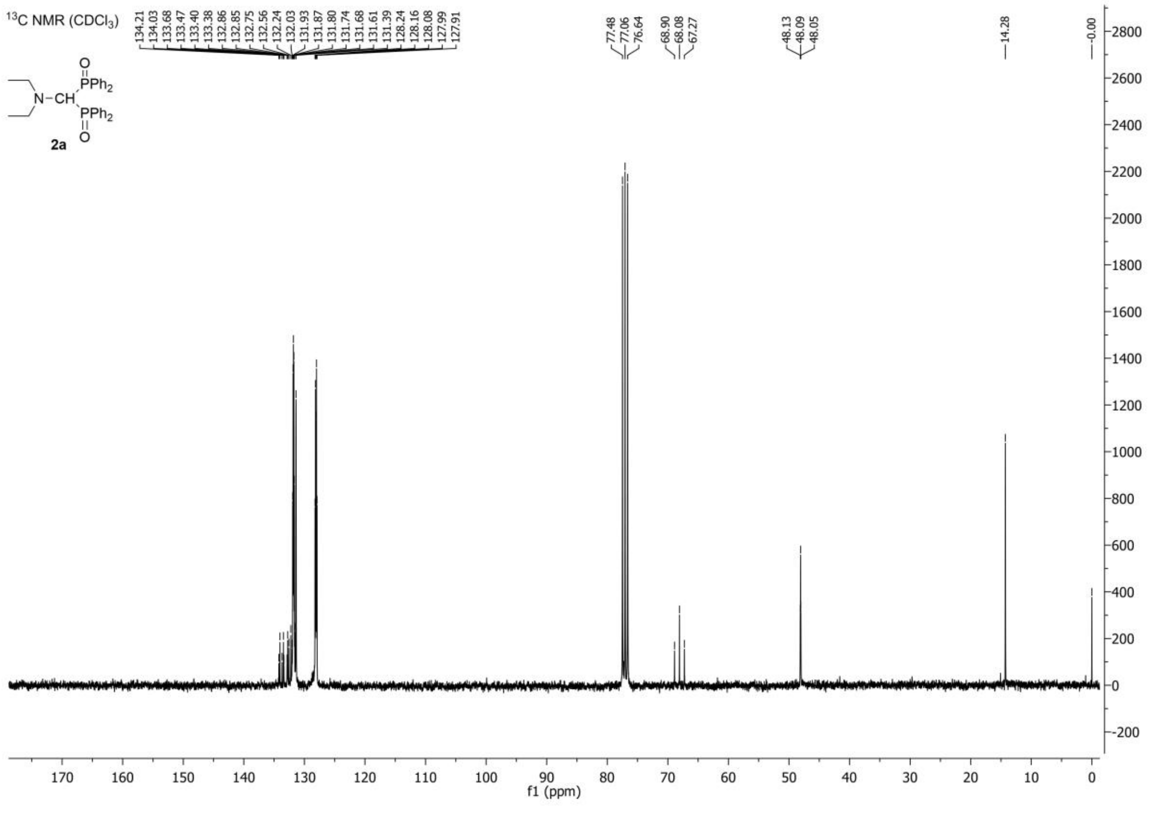
^{13}C NMR (CDCl$_3$)
2a
134.21 134.03 133.68 133.47 133.40 133.38 132.86 132.85 132.75 132.56 132.24 132.03 131.93 131.87 131.80 131.74 131.68 131.61 131.39 128.24 128.16 128.08 127.99 127.91
77.48 77.06 76.64
68.90 68.08 67.27
48.13 48.09 48.05
14.28
-0.00
f1 (ppm)

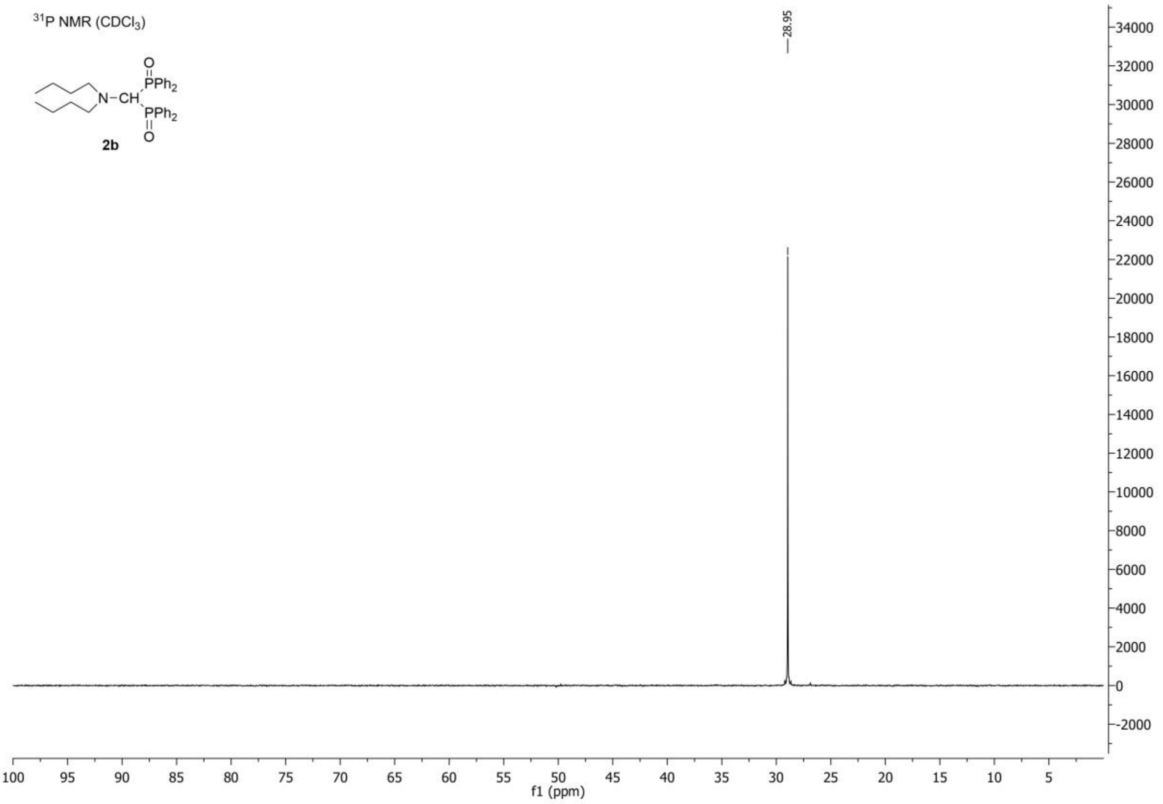
31P NMR (CDCl3)
PPh2
N–CH
PPh2
2b
28.95
f1 (ppm)

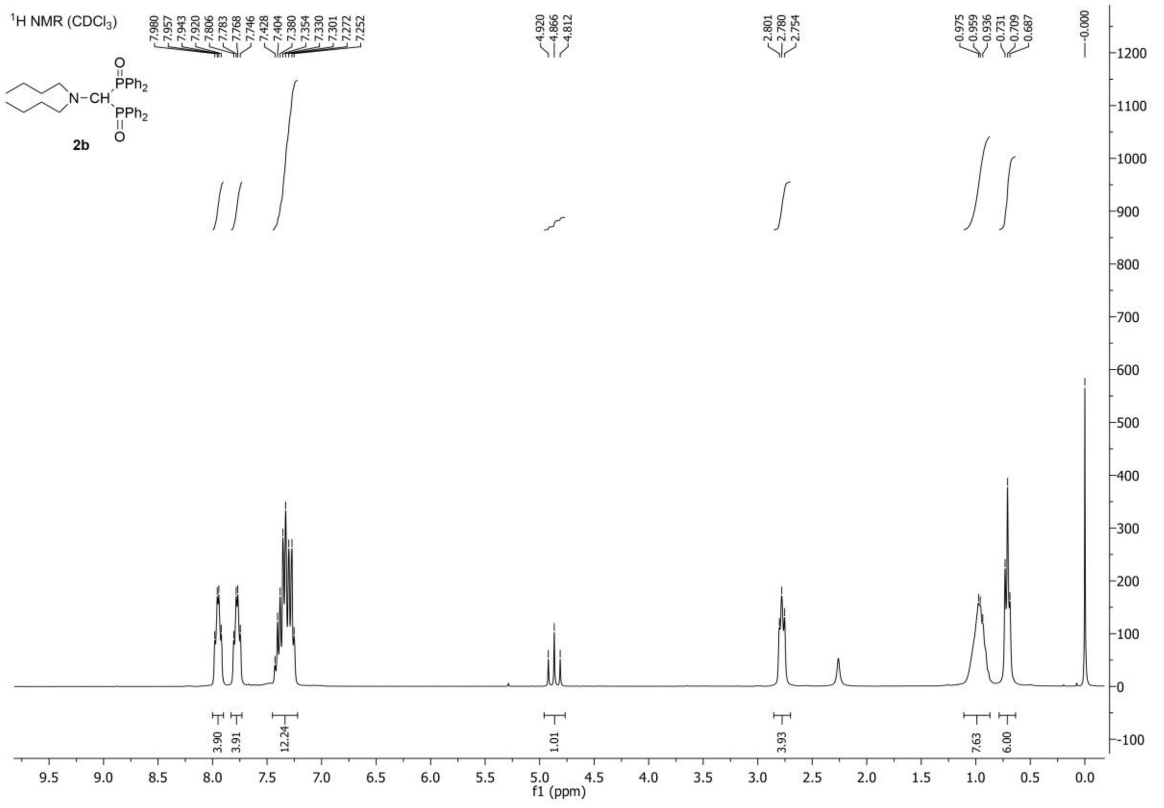
1H NMR (CDCl3)
PPh2
N–CH
PPh2
2b
7.980
7.957
7.943
7.920
7.806
7.783
7.768
7.746
7.428
7.404
7.380
7.354
7.330
7.301
7.272
7.252
4.920
4.866
4.812
2.801
2.780
2.754
0.975
0.959
0.936
0.731
0.709
0.687
-0.000
3.90
3.91
12.24
1.01
3.93
7.63
6.00
f1 (ppm)

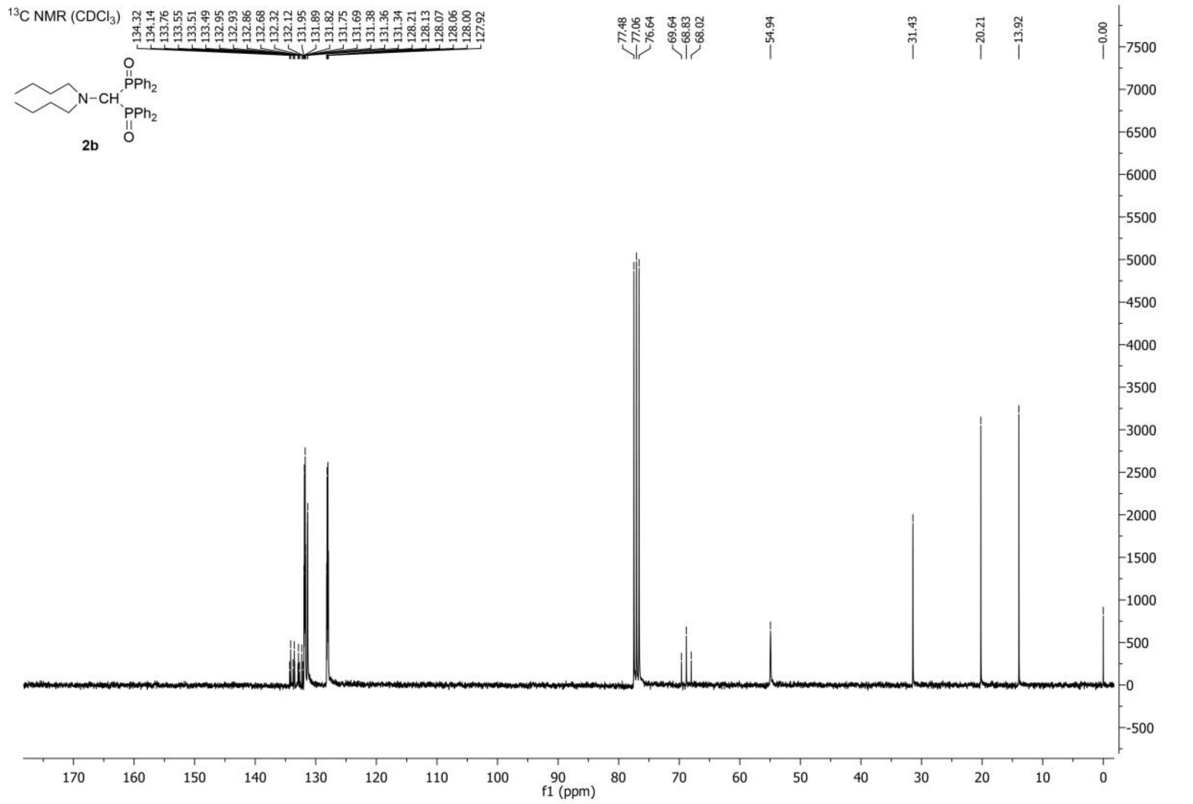

13C NMR (CDCl3)
2b
134.32
134.14
133.76
133.55
133.51
133.49
132.95
132.93
132.86
132.68
132.32
132.12
131.95
131.89
131.82
131.75
131.69
131.38
131.36
131.34
128.21
128.13
128.07
128.06
128.00
127.92
77.48
77.06
76.64
69.64
68.83
68.02
54.94
31.43
20.21
13.92
0.00
f1 (ppm)

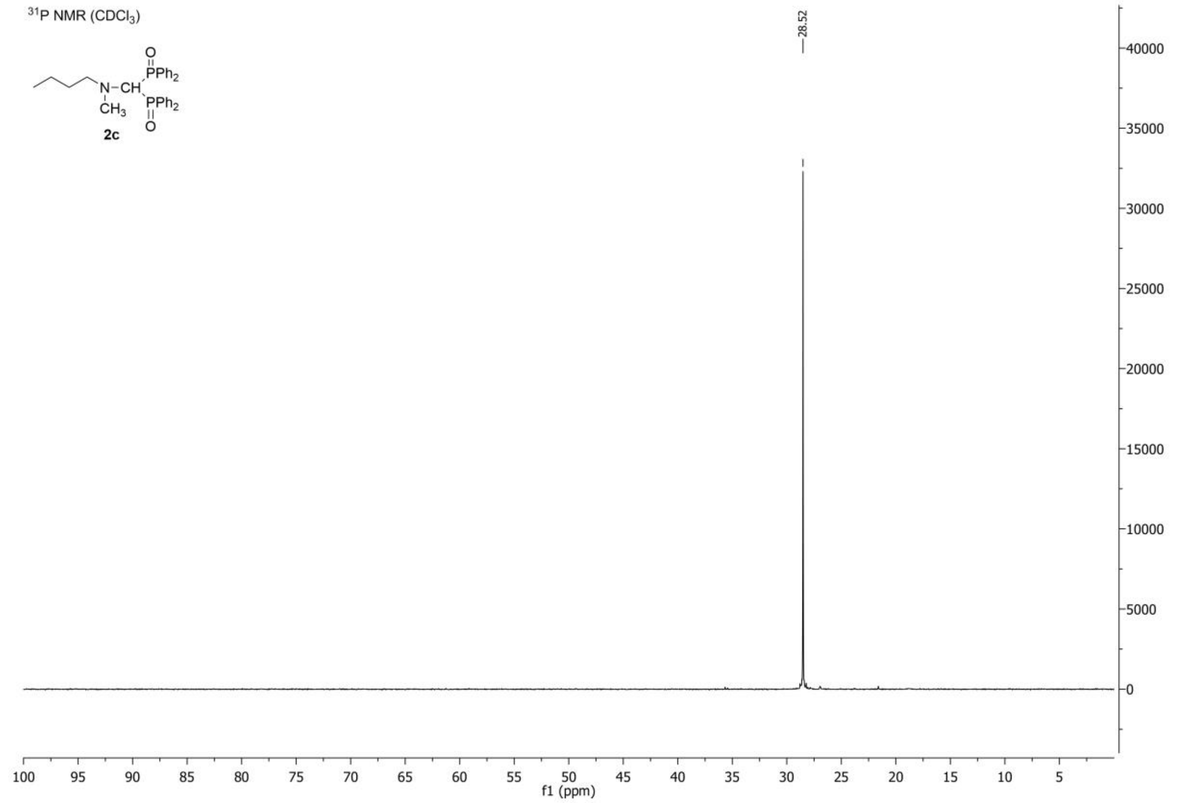

31P NMR (CDCl3)
2c
28.52
f1 (ppm)

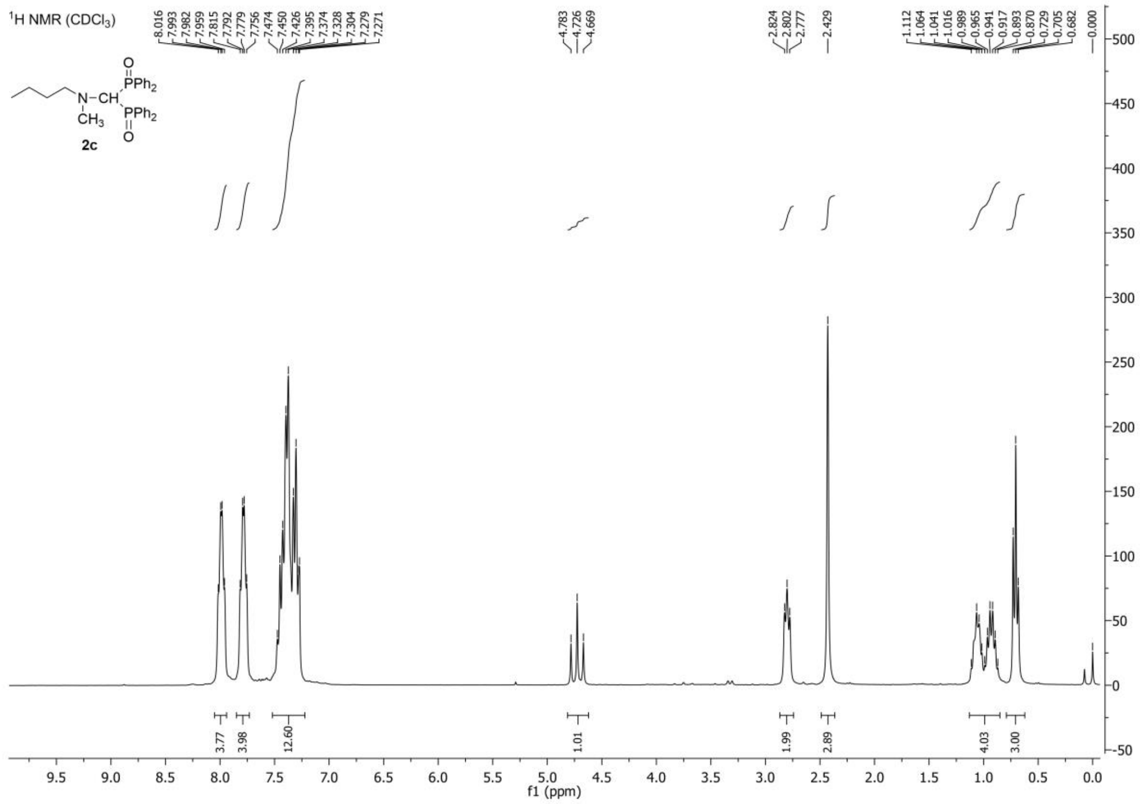
^{1}H NMR (CDCl$_3$)
2c
8.016 7.993 7.982 7.959 7.815 7.792 7.779 7.756 7.474 7.450 7.426 7.395 7.374 7.328 7.304 7.279 7.271
4.783 4.726 4.669
2.824 2.802 2.777 2.429
1.112 1.064 1.041 1.016 0.989 0.965 0.941 0.917 0.893 0.870 0.729 0.705 0.682
0.000
3.77 3.98 12.60 1.01 1.99 2.89 4.03 3.00
f1 (ppm)

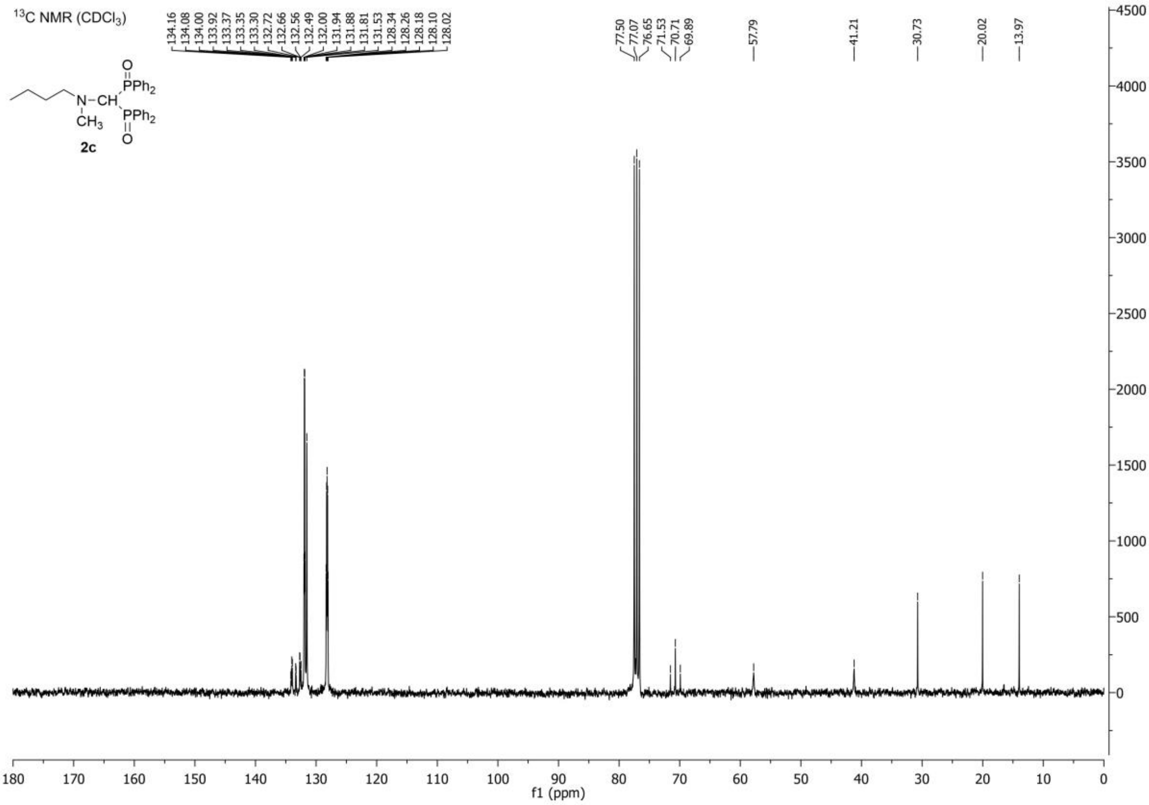
^{13}C NMR (CDCl$_3$)
2c
134.16 134.08 134.00 133.92 133.37 133.35 133.30 132.72 132.66 132.56 132.49 132.00 131.94 131.88 131.81 131.53 128.34 128.26 128.18 128.10 128.02
77.50 77.07 76.65 71.53 70.71 69.89
57.79
41.21
30.73
20.02
13.97
f1 (ppm)

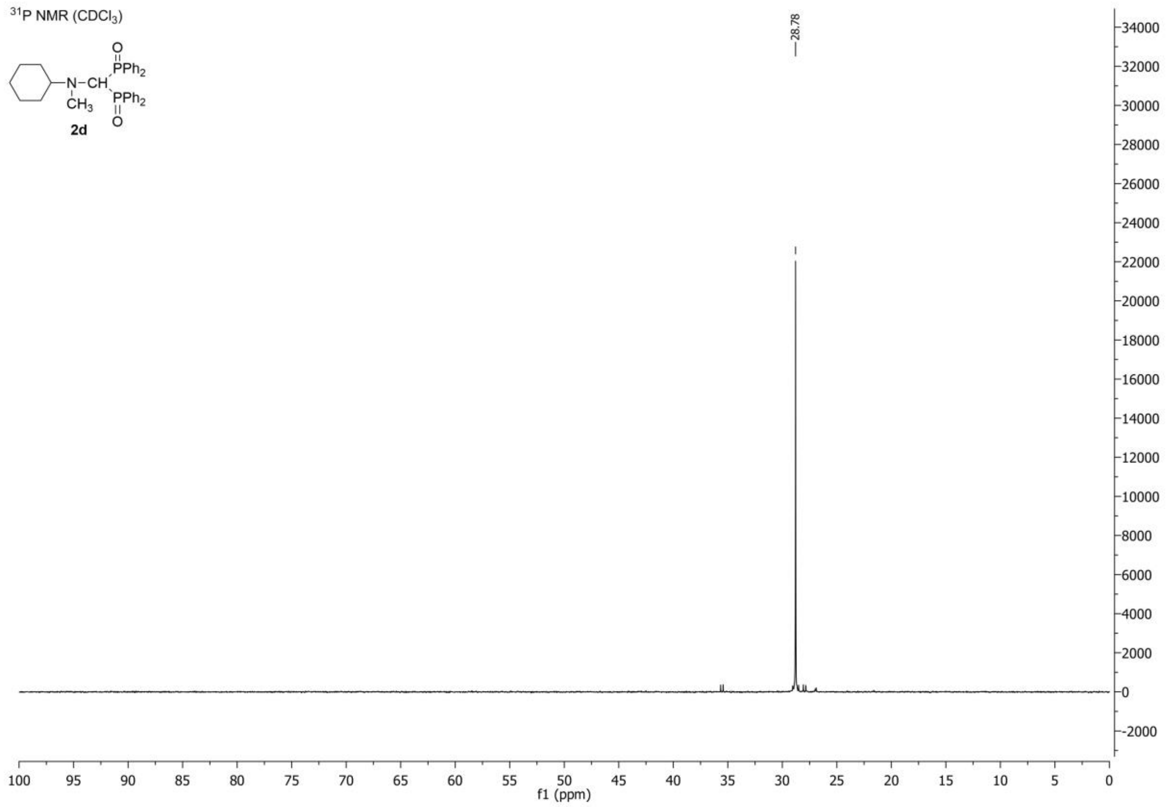
31P NMR (CDCl3)
2d
28.78
f1 (ppm)

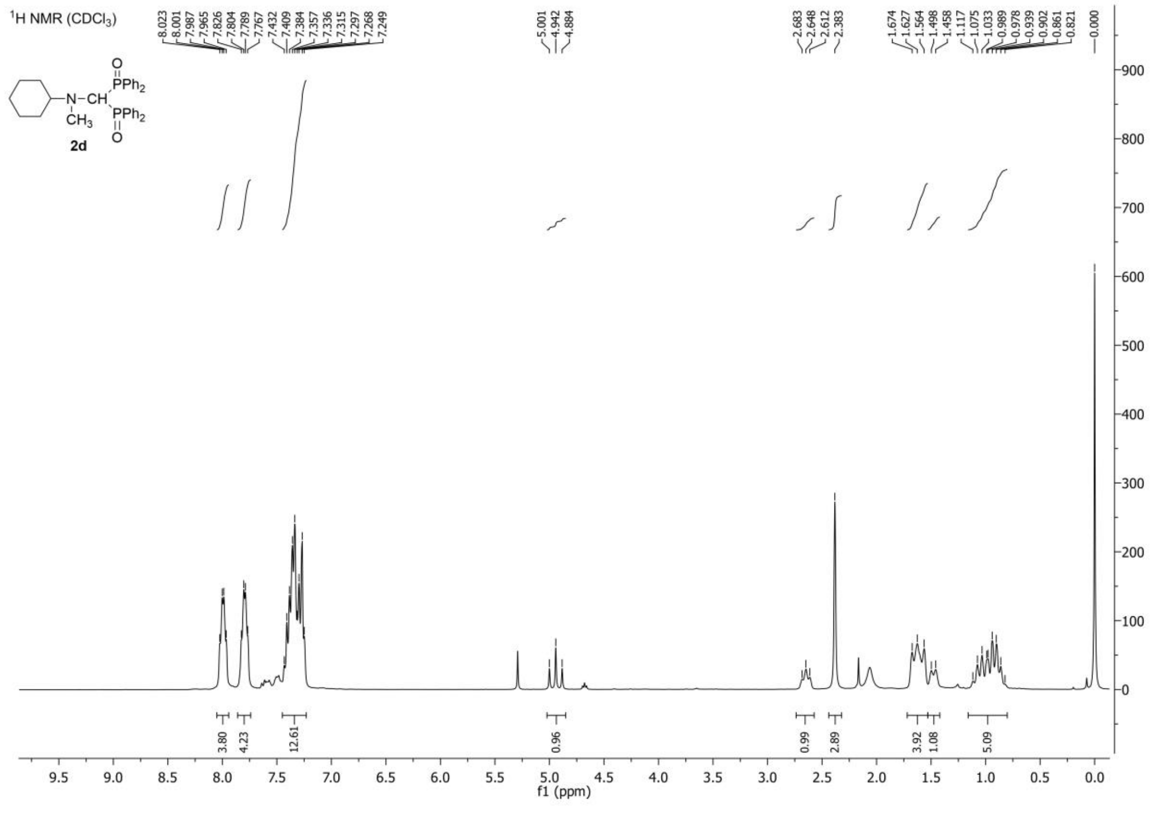
1H NMR (CDCl3)
2d
f1 (ppm)

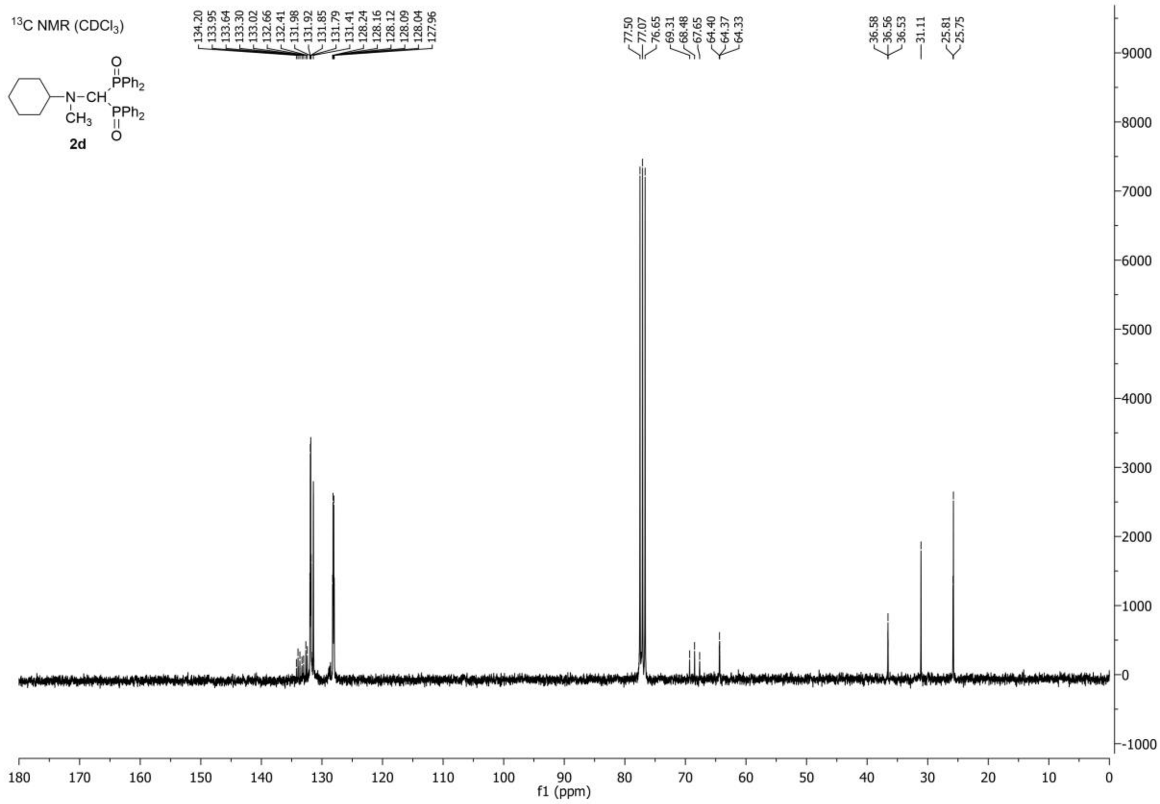

13C NMR (CDCl3)
2d
134.20
133.95
133.64
133.30
133.02
132.66
132.41
131.98
131.92
131.85
131.79
131.41
128.24
128.16
128.12
128.09
128.04
127.96
77.50
77.07
76.65
69.31
68.48
67.65
64.40
64.37
64.33
36.58
36.56
36.53
31.11
25.81
25.75
f1 (ppm)

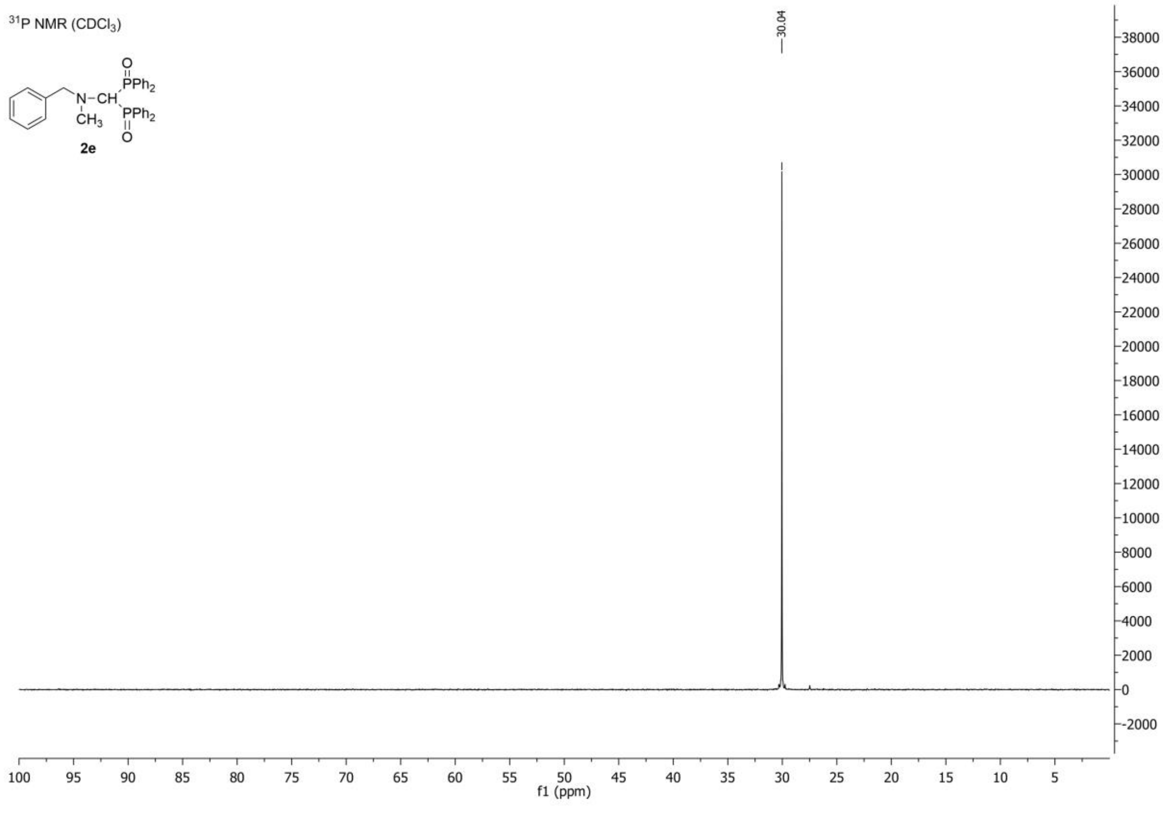

31P NMR (CDCl3)
2e
30.04
f1 (ppm)

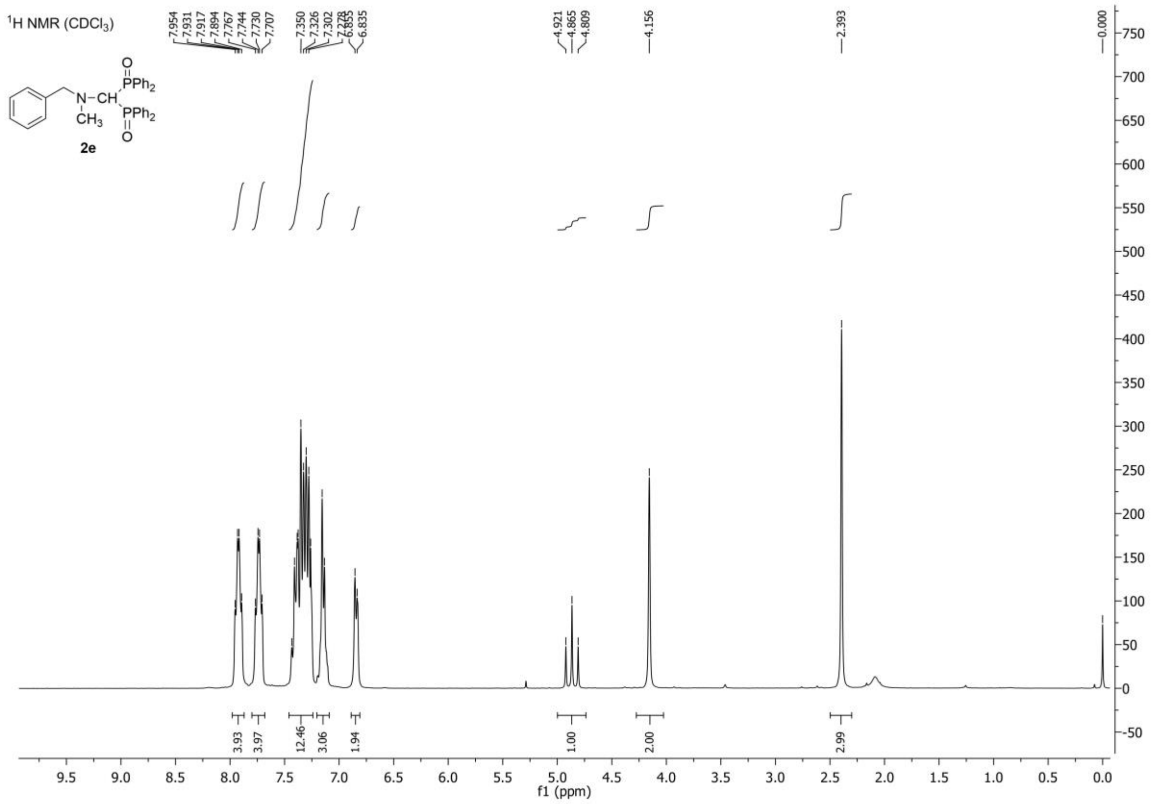

1H NMR (CDCl3)
7.954
7.931
7.917
7.894
7.767
7.744
7.730
7.707
7.350
7.326
7.302
7.278
6.855
6.835
4.921
4.865
4.809
4.156
2.393
0.000
PPh2
N
CH
CH3
PPh2
O
O
2e
3.93
3.97
12.46
3.06
1.94
1.00
2.00
2.99
f1 (ppm)

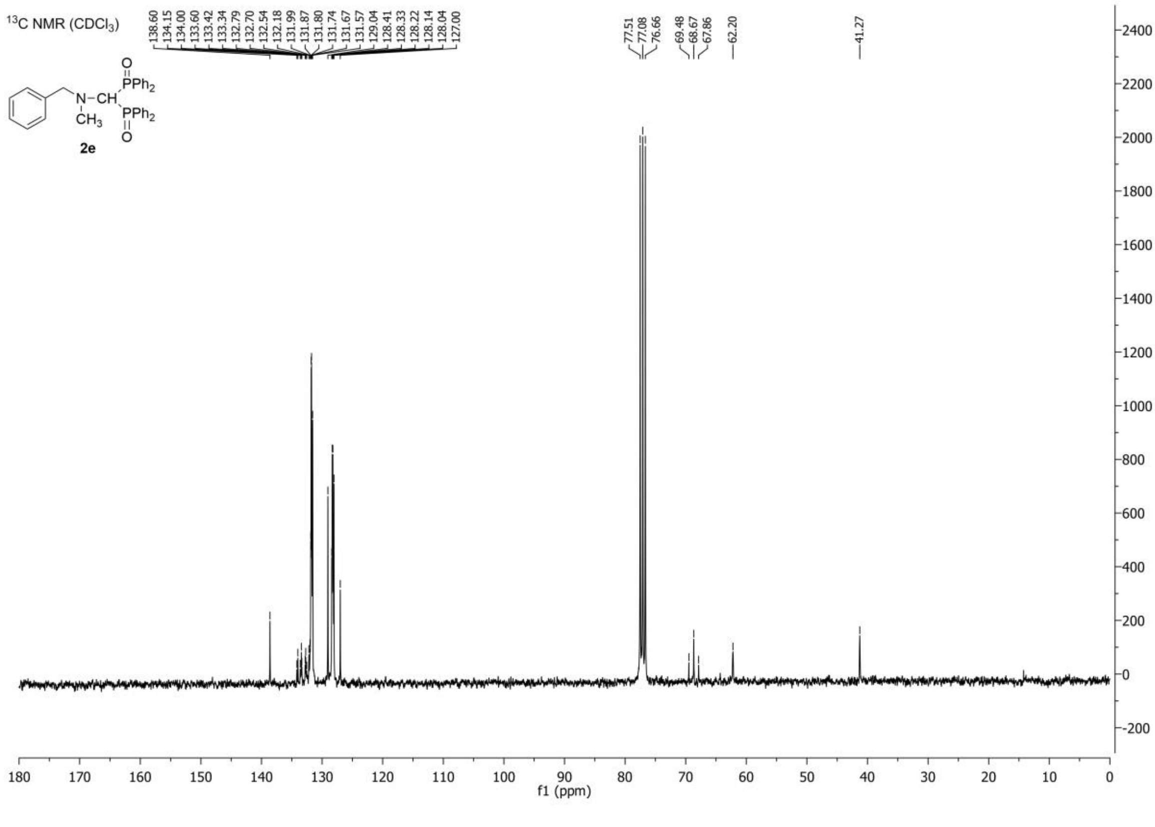

13C NMR (CDCl3)
138.60
134.15
134.00
133.60
133.42
133.34
132.79
132.70
132.54
132.18
131.99
131.87
131.80
131.74
131.67
131.57
129.04
128.41
128.33
128.22
128.14
128.04
127.00
77.51
77.08
76.66
69.48
68.67
67.86
62.20
41.27
PPh2
N
CH
CH3
PPh2
O
O
2e
f1 (ppm)

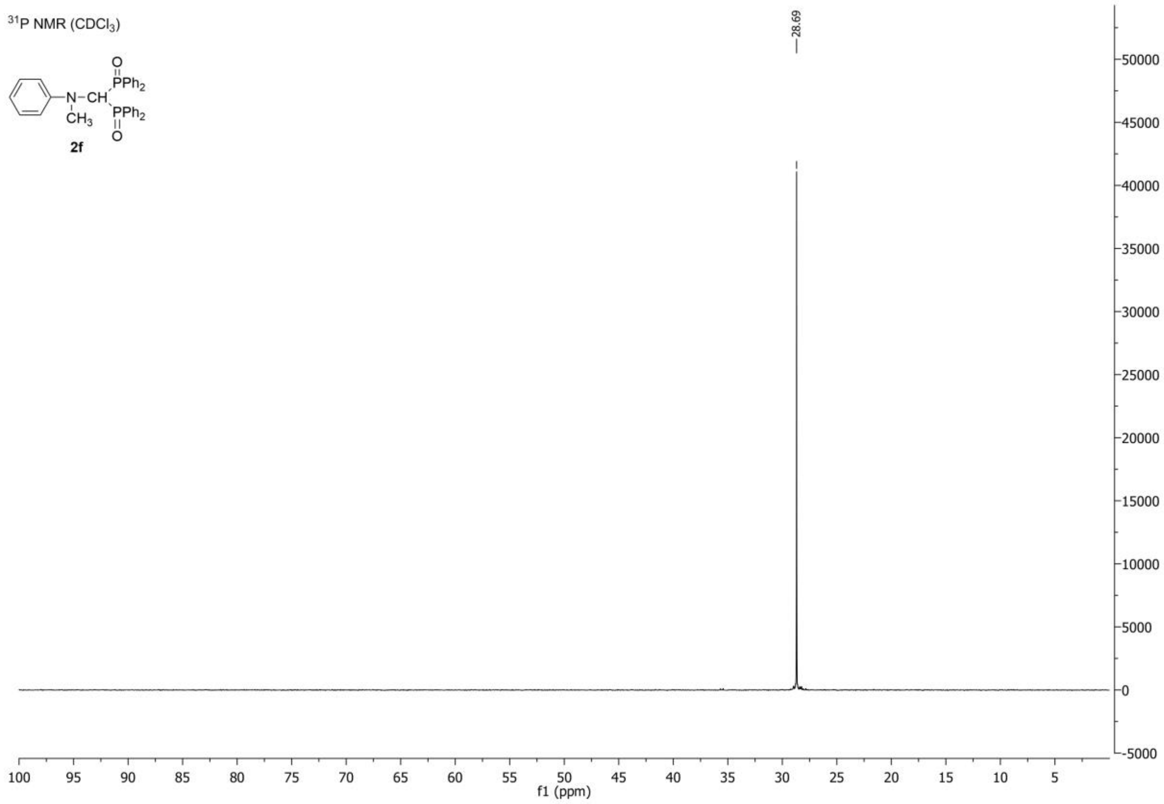
31P NMR (CDCl3)
2f
28.69
f1 (ppm)

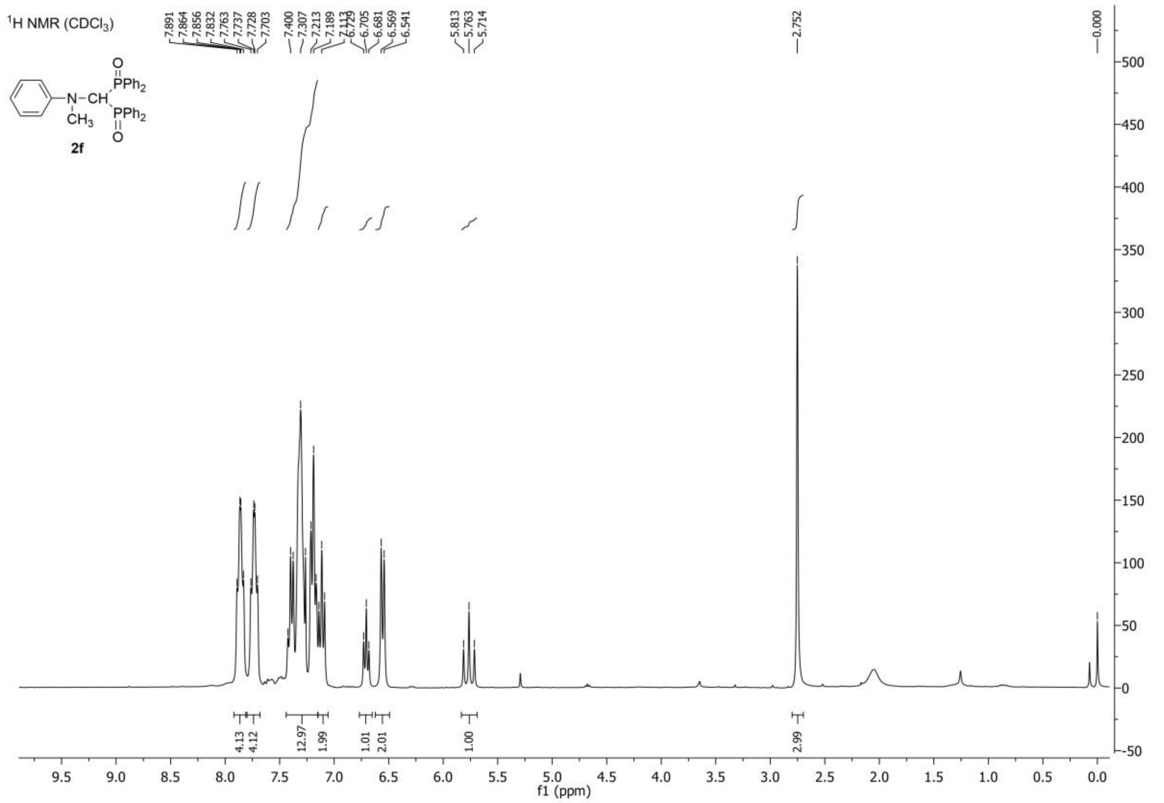
1H NMR (CDCl3)
2f
7.891 7.864 7.856 7.832 7.763 7.737 7.728 7.703
7.400 7.307 7.213 7.189 7.113 6.729 6.705 6.681 6.569 6.541
5.813 5.763 5.714
2.752
0.000
4.13 4.12 12.97 1.99 1.01 2.01 1.00 2.99
f1 (ppm)

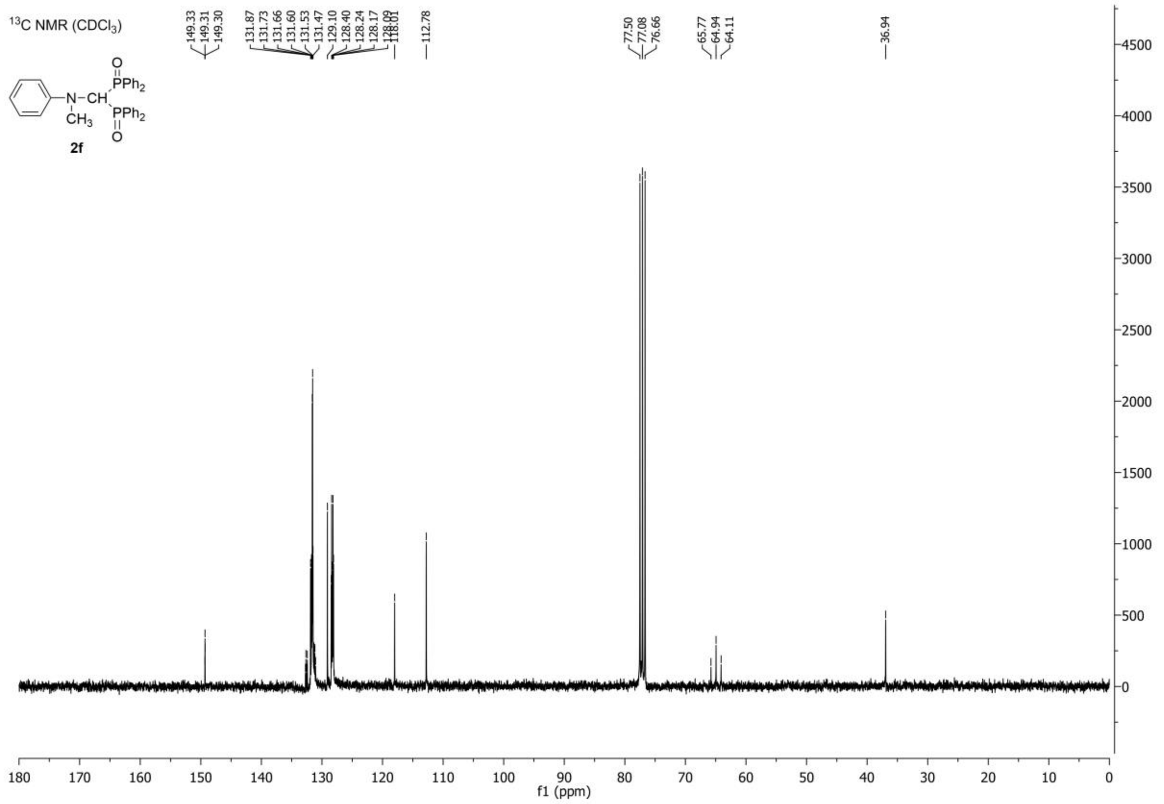
^{13}C NMR (CDCl$_3$)
2f
149.33
149.31
149.30
131.87
131.73
131.66
131.60
131.53
131.47
129.10
128.40
128.24
128.17
128.09
118.01
112.78
77.50
77.08
76.66
65.77
64.94
64.11
36.94
f1 (ppm)

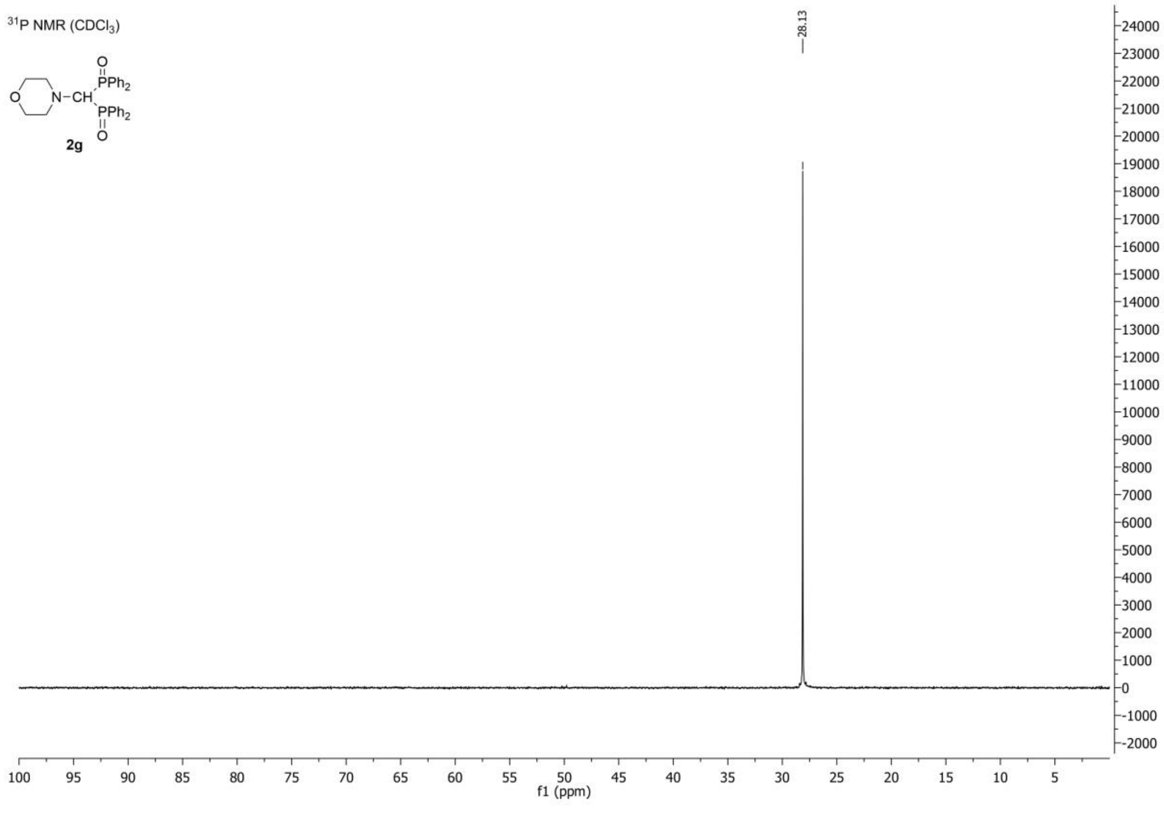
^{31}P NMR (CDCl$_3$)
2g
28.13
f1 (ppm)

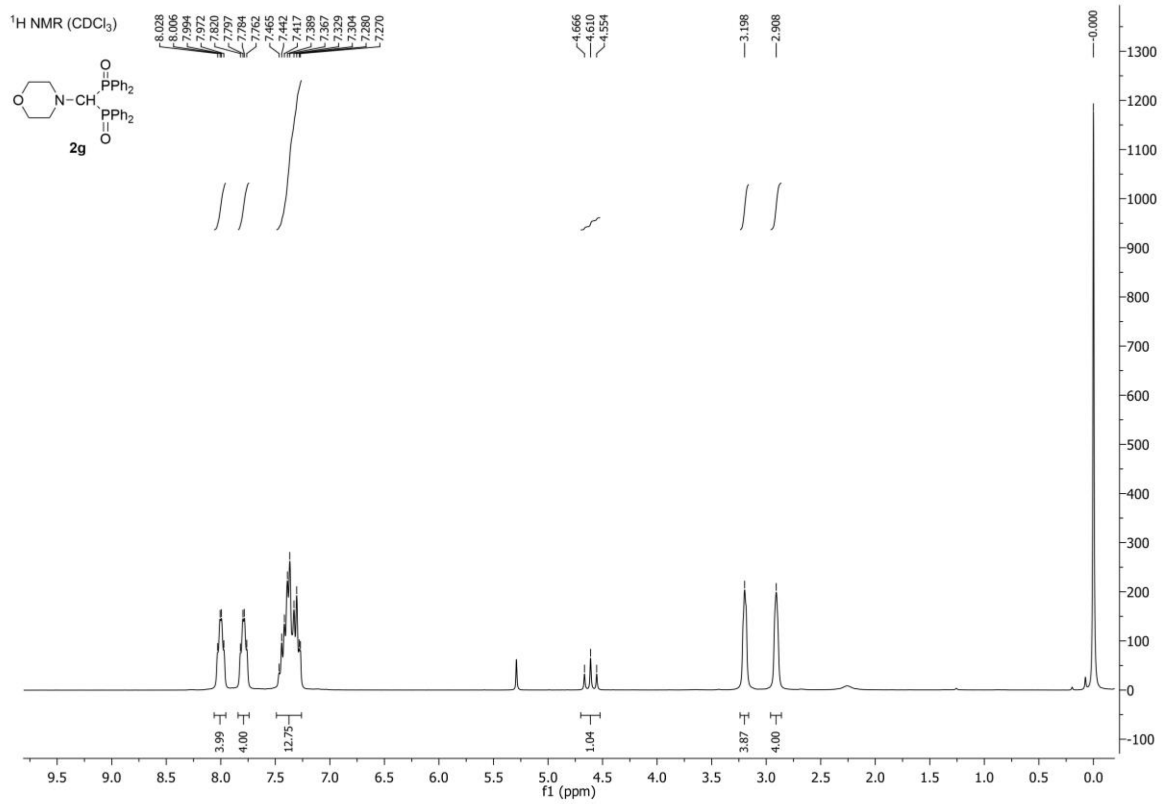

^{1}H NMR (CDCl$_3$)
2g
8.028 8.006 7.994 7.972 7.820 7.797 7.784 7.762 7.465 7.442 7.417 7.389 7.367 7.329 7.304 7.280 7.270
4.666 4.610 4.554
3.198
2.908
0.000
3.99 4.00 12.75 1.04 3.87 4.00
f1 (ppm)

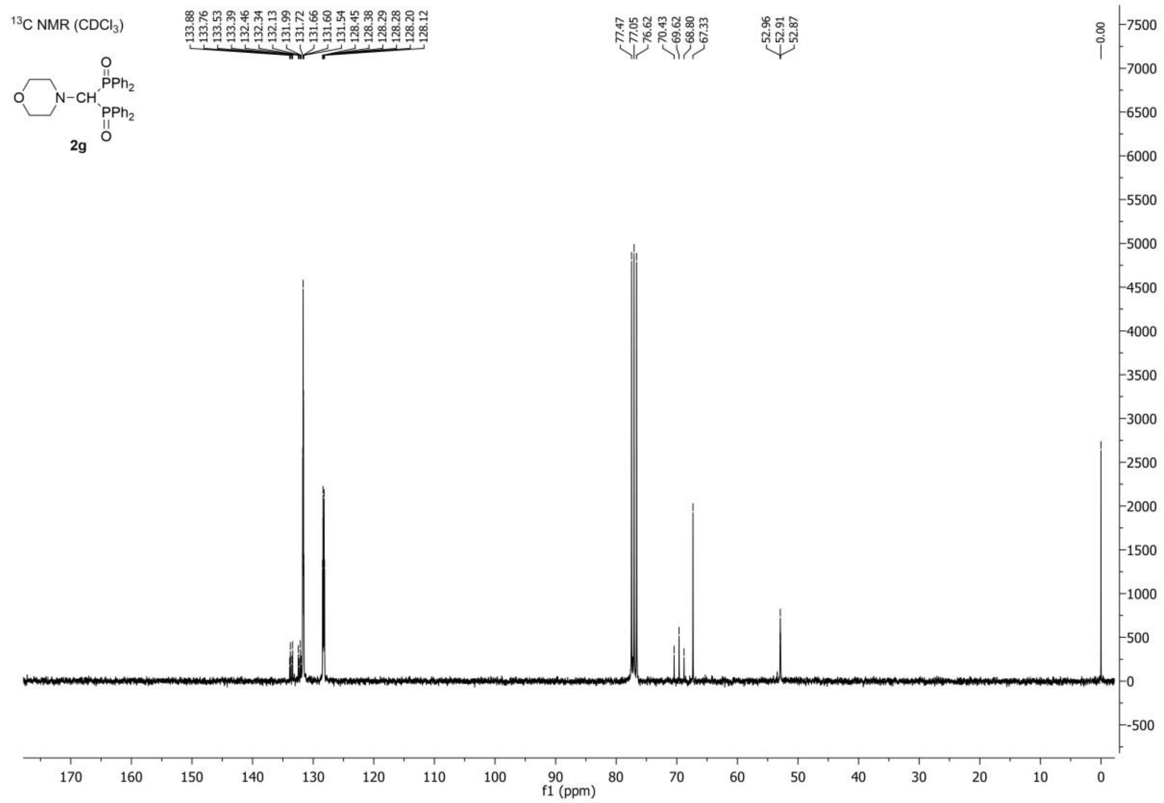

^{13}C NMR (CDCl$_3$)
2g
133.88 133.76 133.53 133.39 132.46 132.34 132.13 131.99 131.72 131.66 131.60 131.54 128.45 128.38 128.29 128.28 128.20 128.12
77.47 77.05 76.62 70.43 69.62 68.80 67.33
52.96 52.91 52.87
0.00
f1 (ppm)

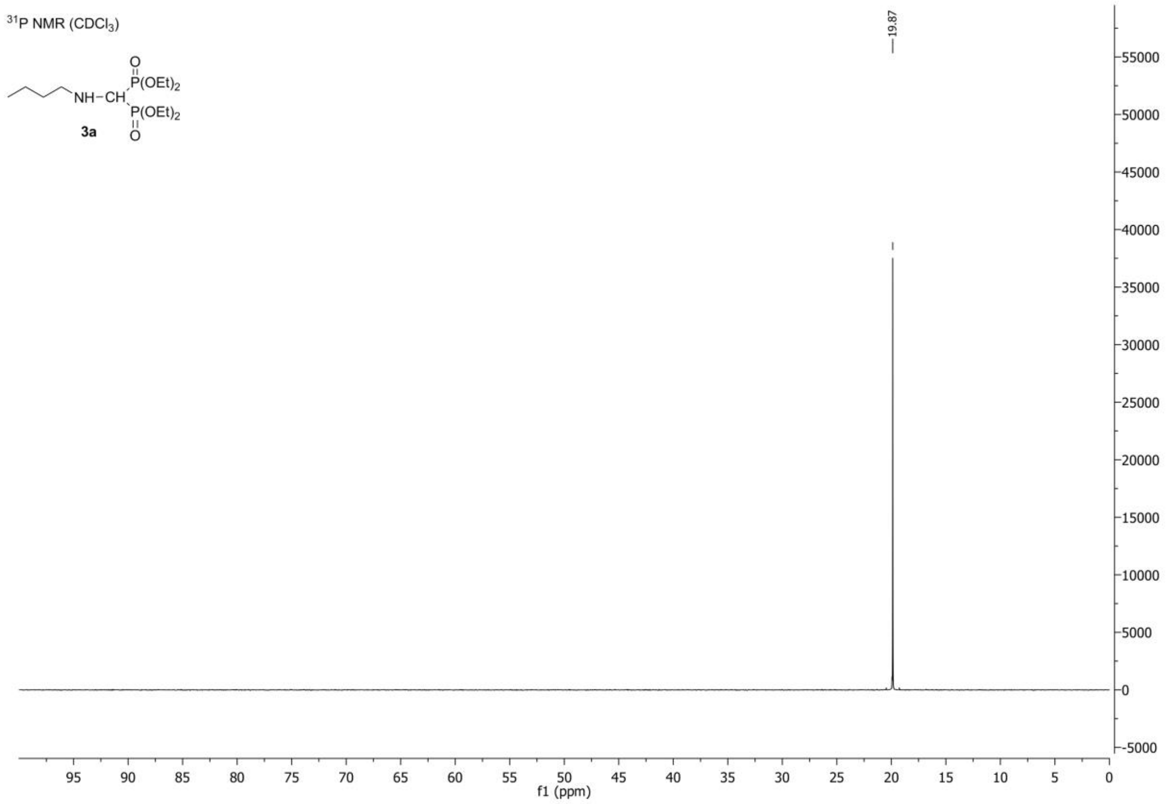
^{31}P NMR (CDCl$_3$)
NH−CH
P(OEt)$_2$
P(OEt)$_2$
O
O
3a
19.87
f1 (ppm)

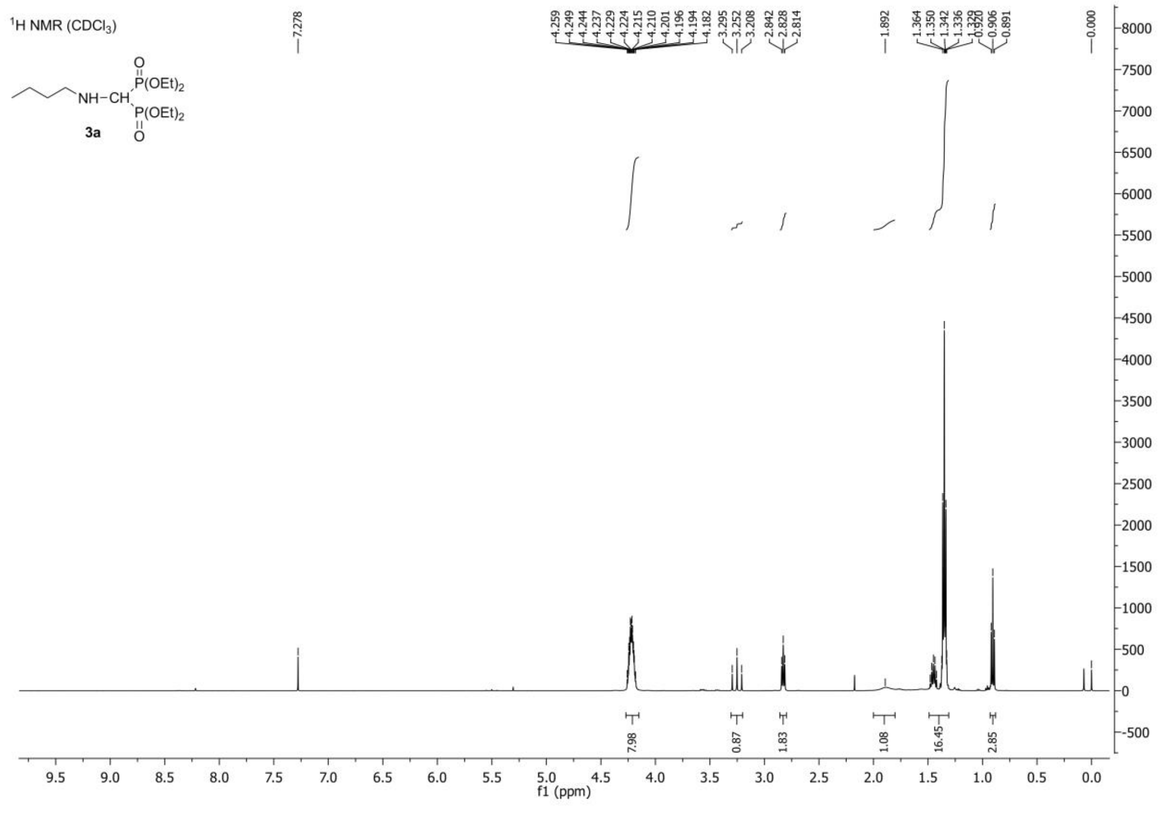
^{1}H NMR (CDCl$_3$)
NH−CH
P(OEt)$_2$
P(OEt)$_2$
O
O
3a
7.278
4.259
4.249
4.244
4.237
4.229
4.224
4.215
4.210
4.201
4.196
4.194
4.182
3.295
3.252
3.208
2.842
2.828
2.814
1.892
1.364
1.350
1.342
1.336
1.329
0.920
0.906
0.891
0.000
7.98
0.87
1.83
1.08
16.45
2.85
f1 (ppm)

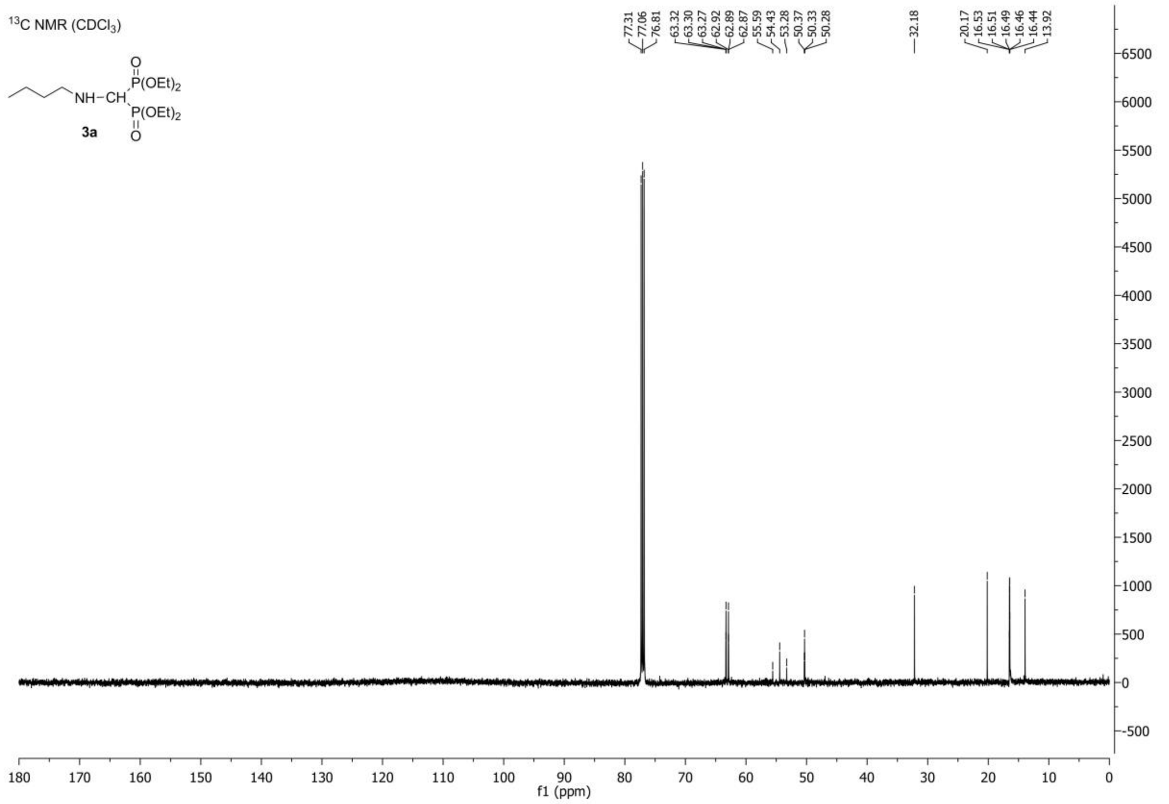

^{13}C NMR (CDCl$_3$)
NH−CH
P(OEt)$_2$
P(OEt)$_2$
3a
77.31
77.06
76.81
63.32
63.30
63.27
62.92
62.89
62.87
55.59
54.43
53.28
50.37
50.33
50.28
32.18
20.17
16.53
16.51
16.49
16.46
16.44
13.92
f1 (ppm)

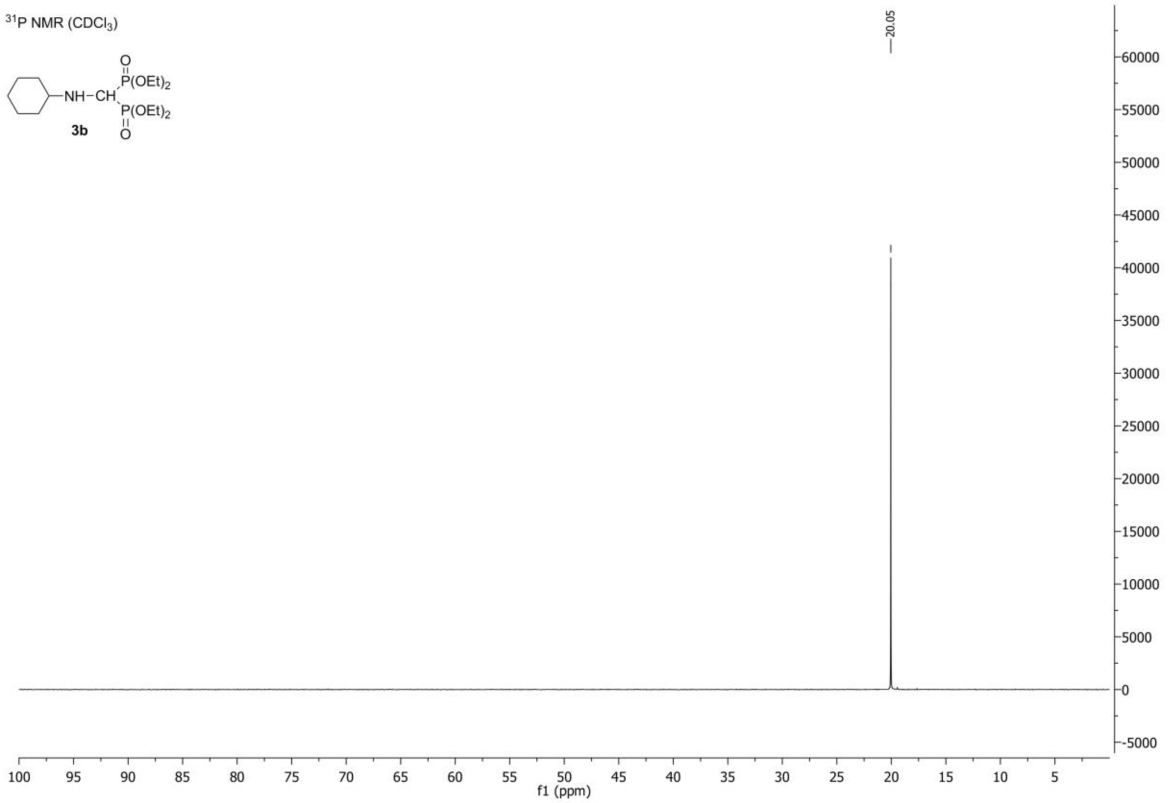

^{31}P NMR (CDCl$_3$)
NH−CH
P(OEt)$_2$
P(OEt)$_2$
3b
20.05
f1 (ppm)

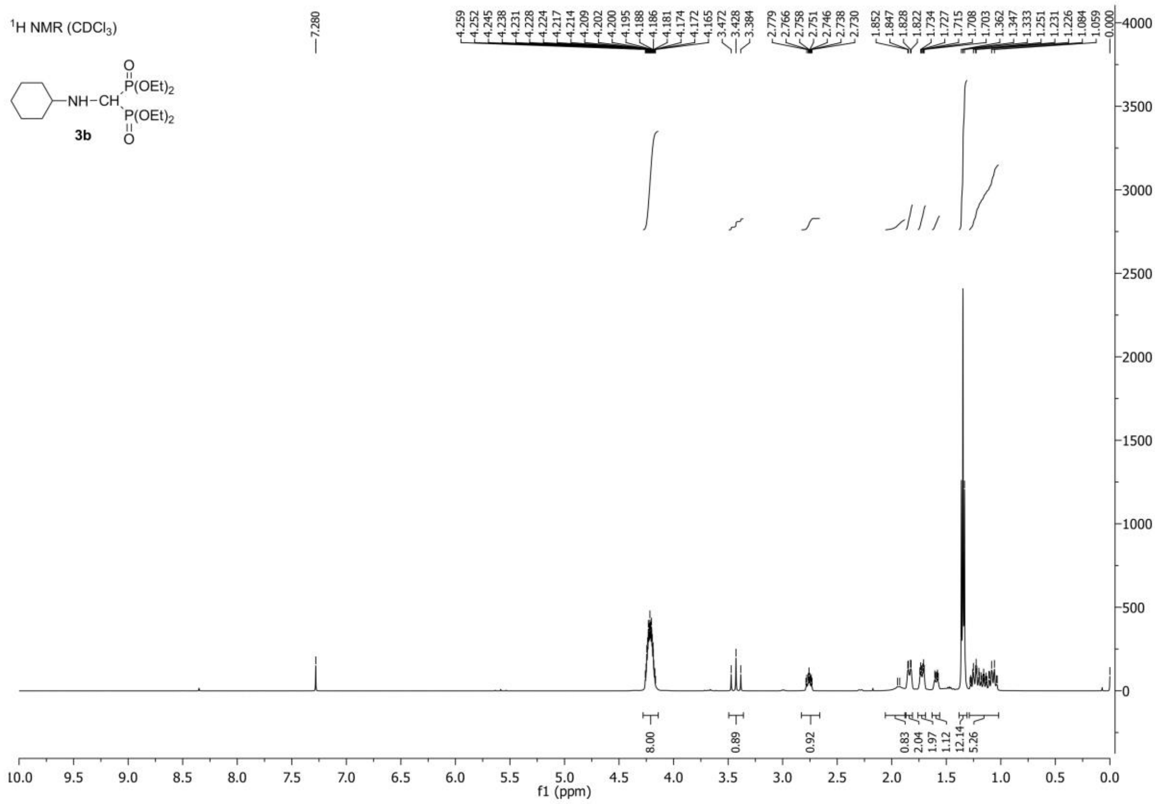

1H NMR (CDCl3)
3b
8.00
0.89
0.92
0.83
2.04
1.97
1.12
12.14
5.26
f1 (ppm)

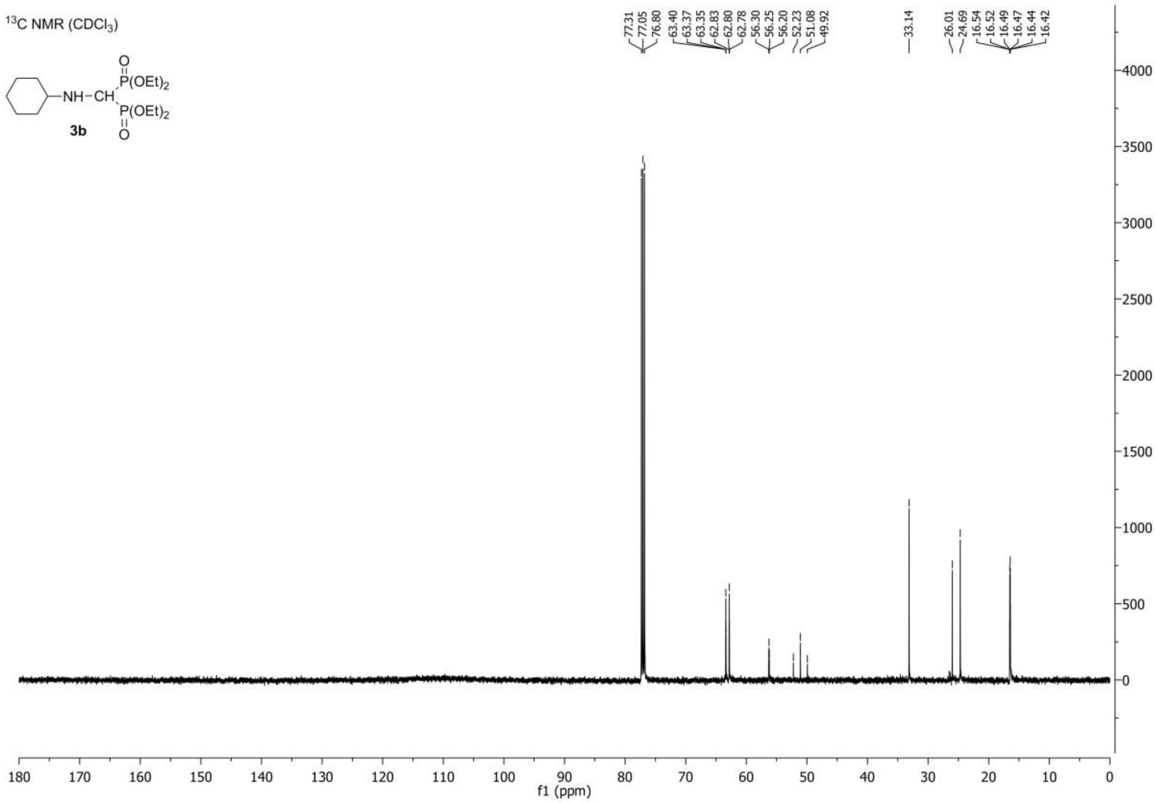

13C NMR (CDCl3)
3b
f1 (ppm)

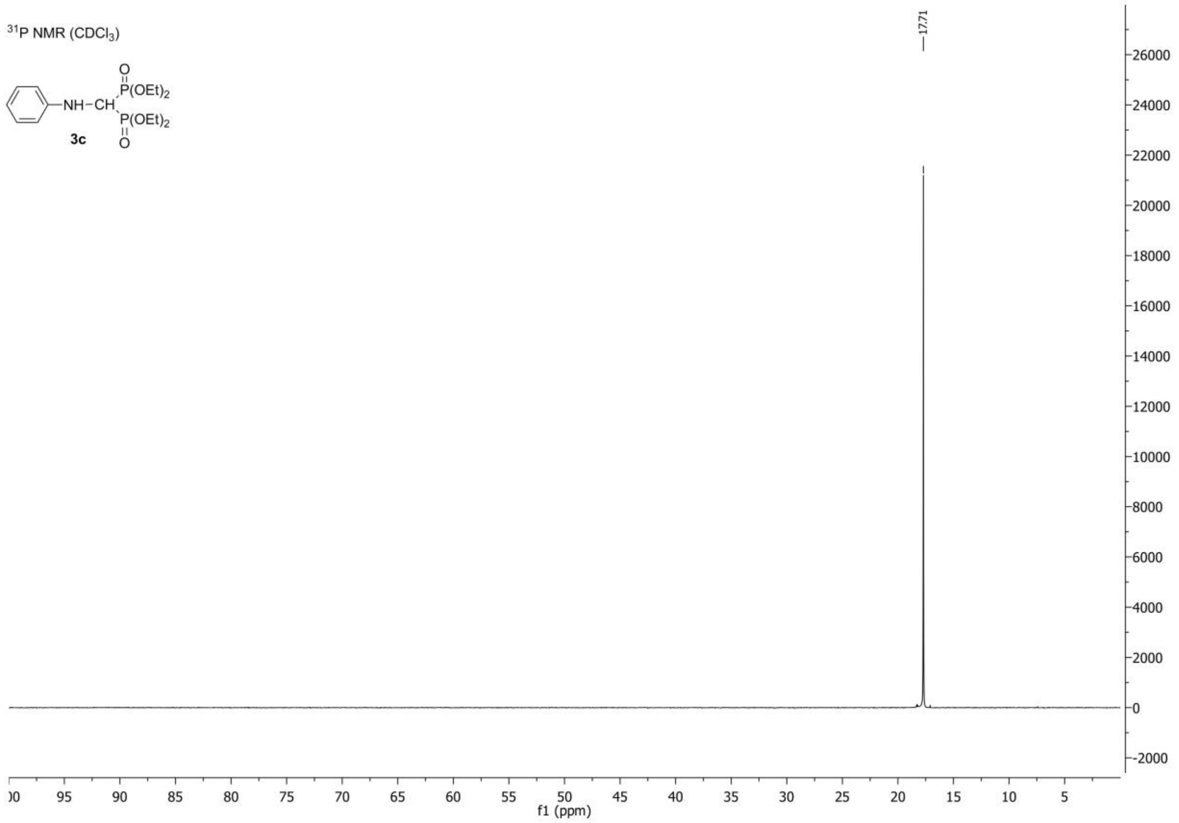

^{31}P NMR (CDCl$_3$)
3c
17.71
f1 (ppm)

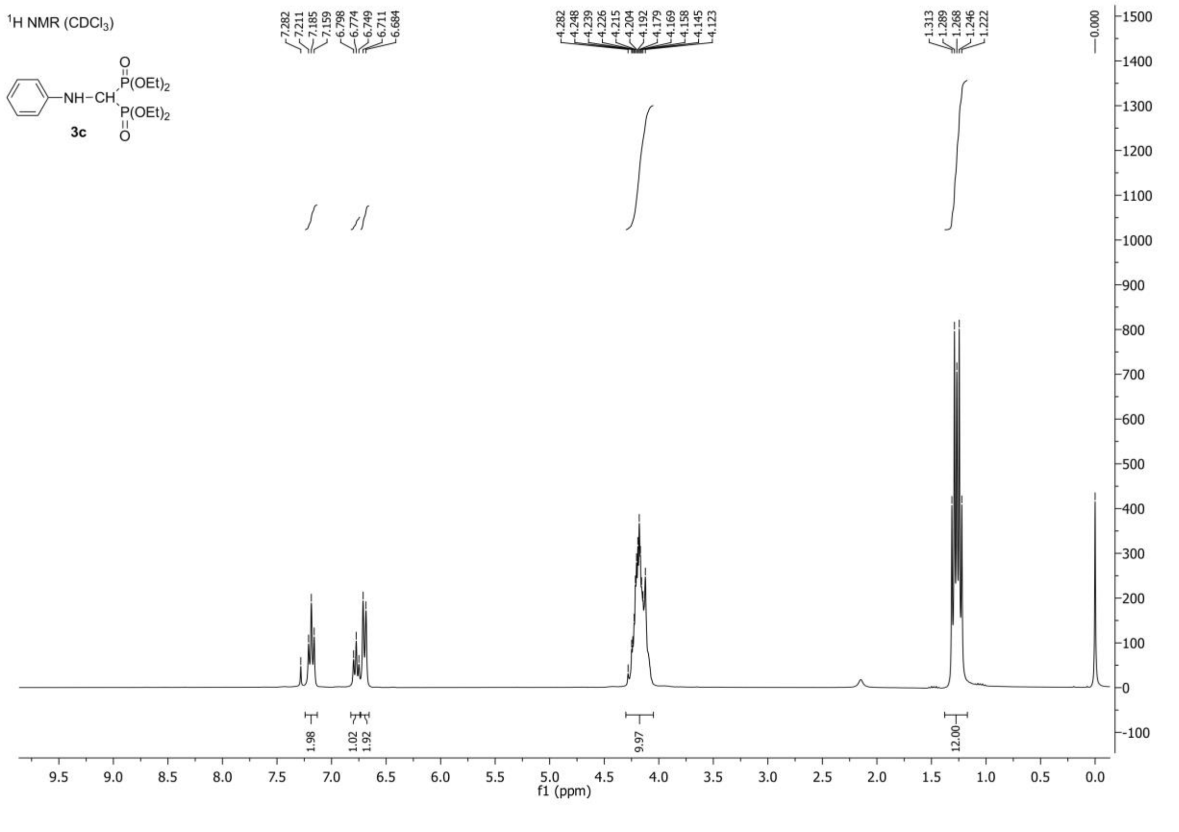

^{1}H NMR (CDCl$_3$)
3c
7.282
7.211
7.185
7.159
6.798
6.774
6.749
6.711
6.684
4.282
4.248
4.239
4.226
4.215
4.204
4.192
4.179
4.169
4.158
4.145
4.123
1.313
1.289
1.268
1.246
1.222
0.000
1.98
1.02
1.92
9.97
12.00
f1 (ppm)

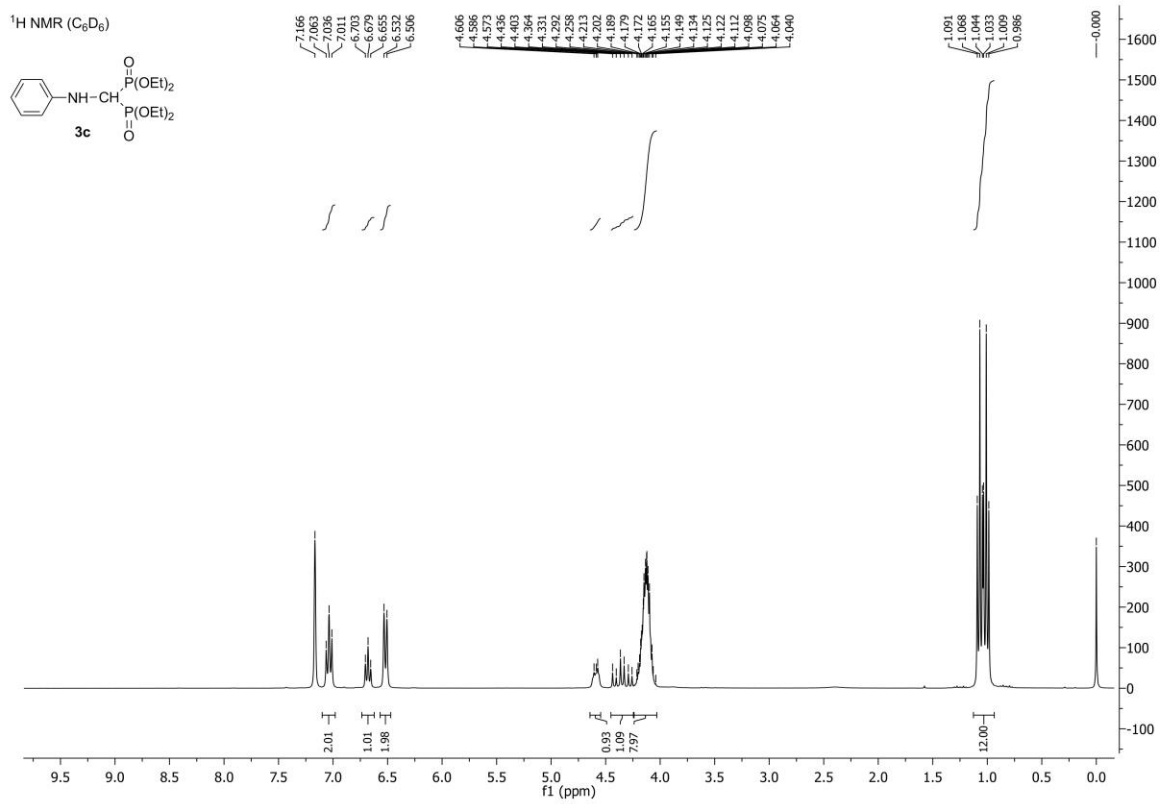

1H NMR (C6D6)
P(OEt)2
NH−CH
3c
7.166 7.063 7.036 7.011 6.703 6.679 6.655 6.532 6.506
4.606 4.586 4.573 4.436 4.403 4.364 4.331 4.292 4.258 4.213 4.202 4.189 4.179 4.172 4.165 4.155 4.149 4.134 4.125 4.122 4.112 4.098 4.075 4.064 4.040
1.091 1.068 1.044 1.033 1.009 0.986
0.000
2.01 1.01 1.98 0.93 1.09 7.97 12.00
f1 (ppm)

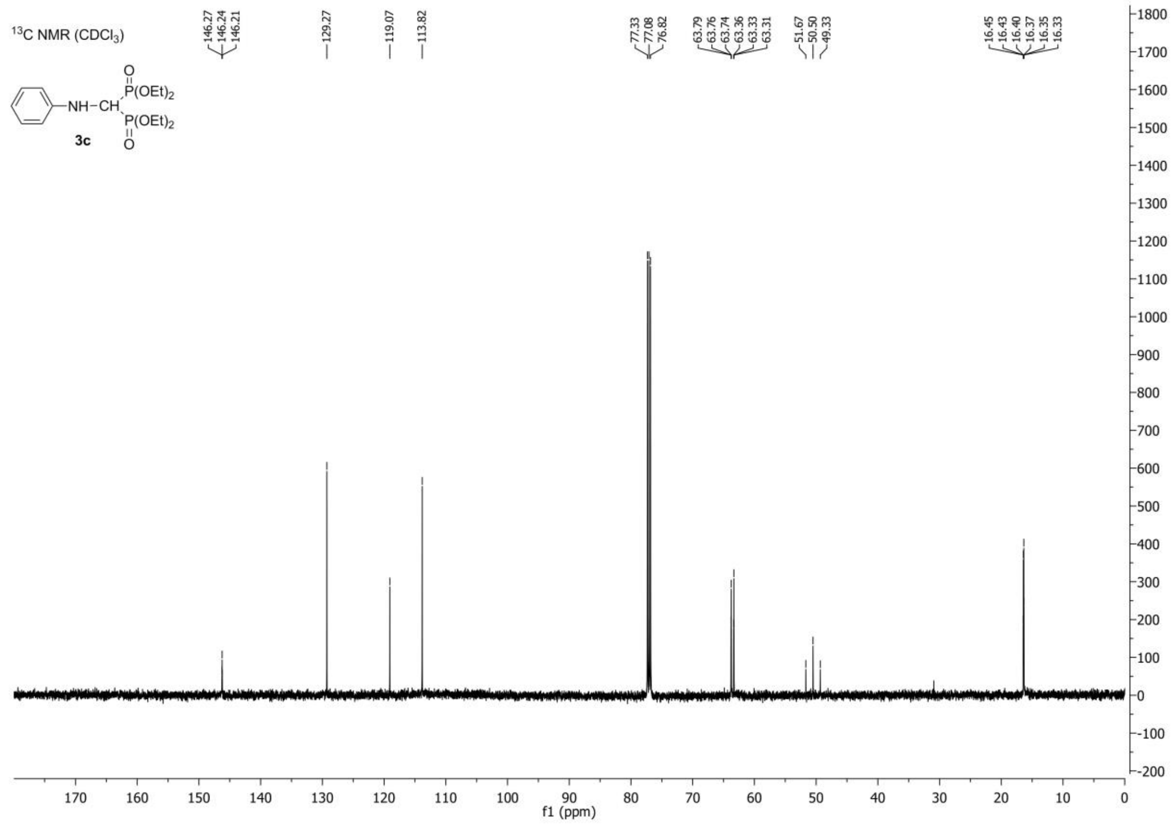

13C NMR (CDCl3)
P(OEt)2
NH−CH
3c
146.27 146.24 146.21
129.27
119.07
113.82
77.33 77.08 76.82
63.79 63.76 63.74 63.36 63.33 63.31
51.67 50.50 49.33
16.45 16.43 16.40 16.37 16.35 16.33
f1 (ppm)

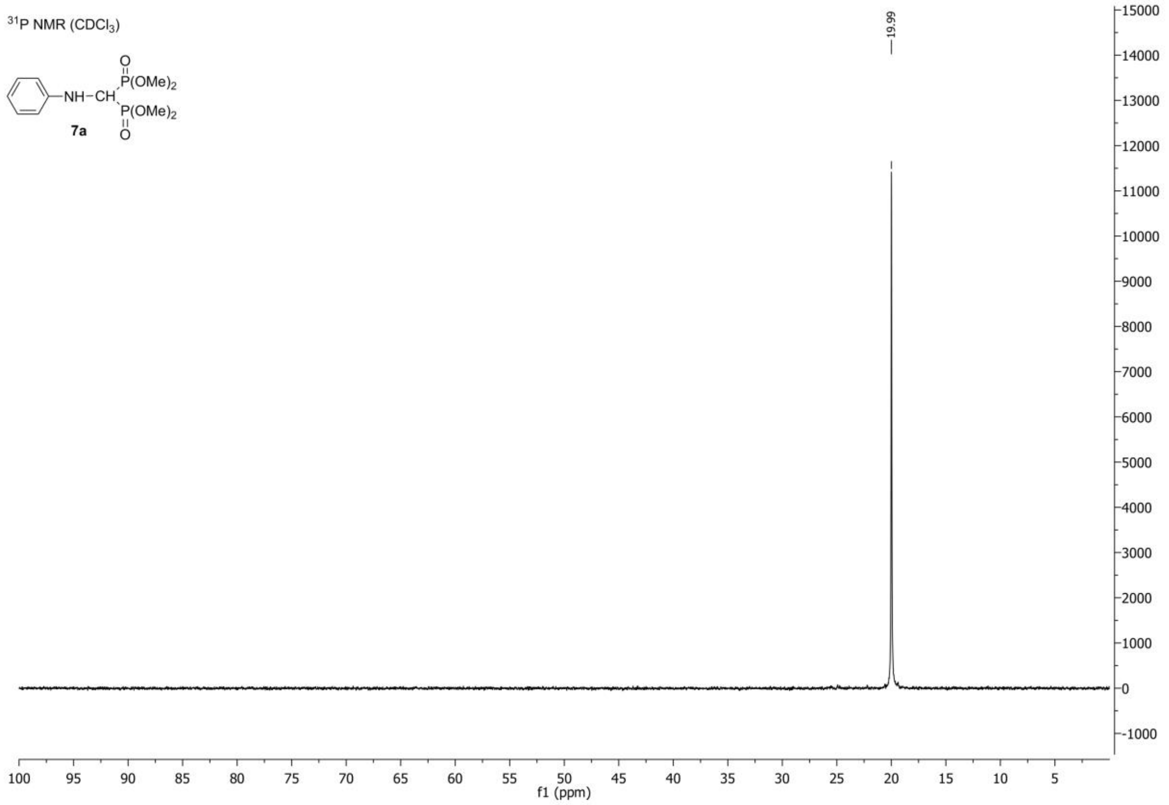

^{31}P NMR (CDCl$_3$)
NH−CH
P(OMe)$_2$
P(OMe)$_2$
7a
19.99
f1 (ppm)

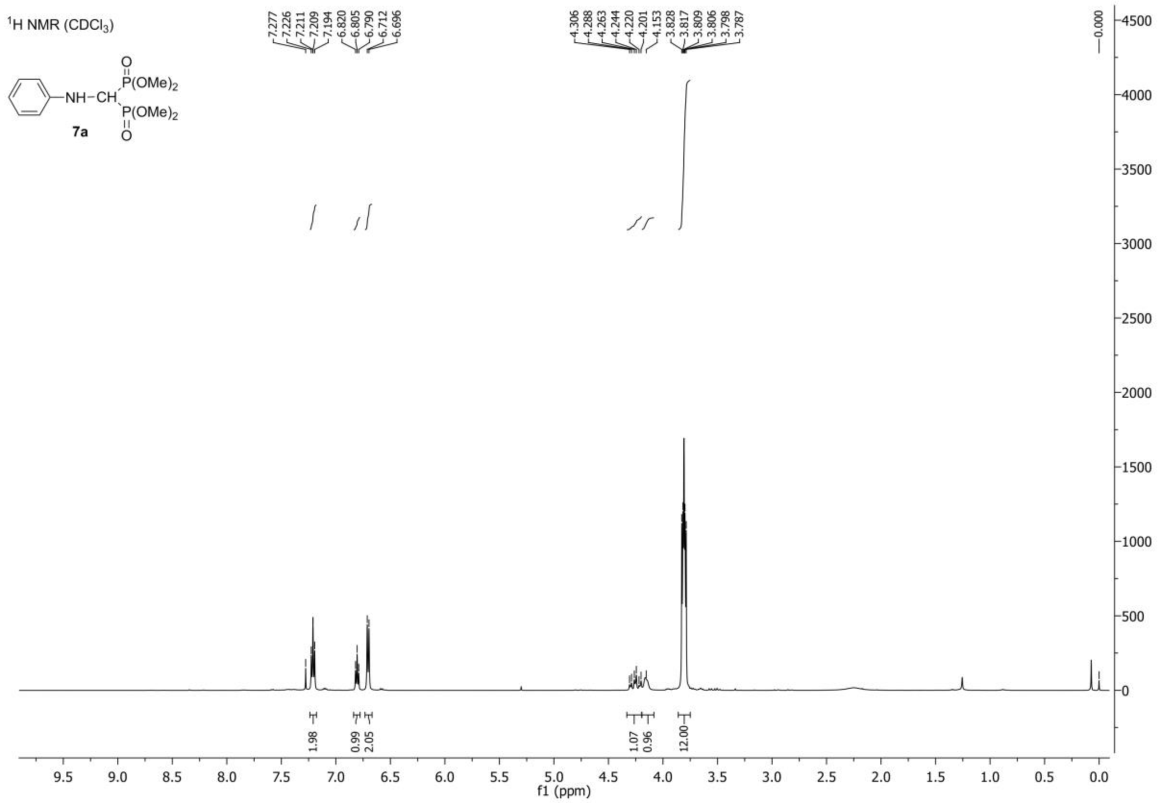

^{1}H NMR (CDCl$_3$)
NH−CH
P(OMe)$_2$
P(OMe)$_2$
7a
7.277
7.226
7.211
7.209
7.194
6.820
6.805
6.790
6.712
6.696
4.306
4.288
4.263
4.244
4.220
4.201
4.153
3.828
3.817
3.809
3.806
3.798
3.787
0.000
1.98
0.99
2.05
1.07
0.96
12.00
f1 (ppm)

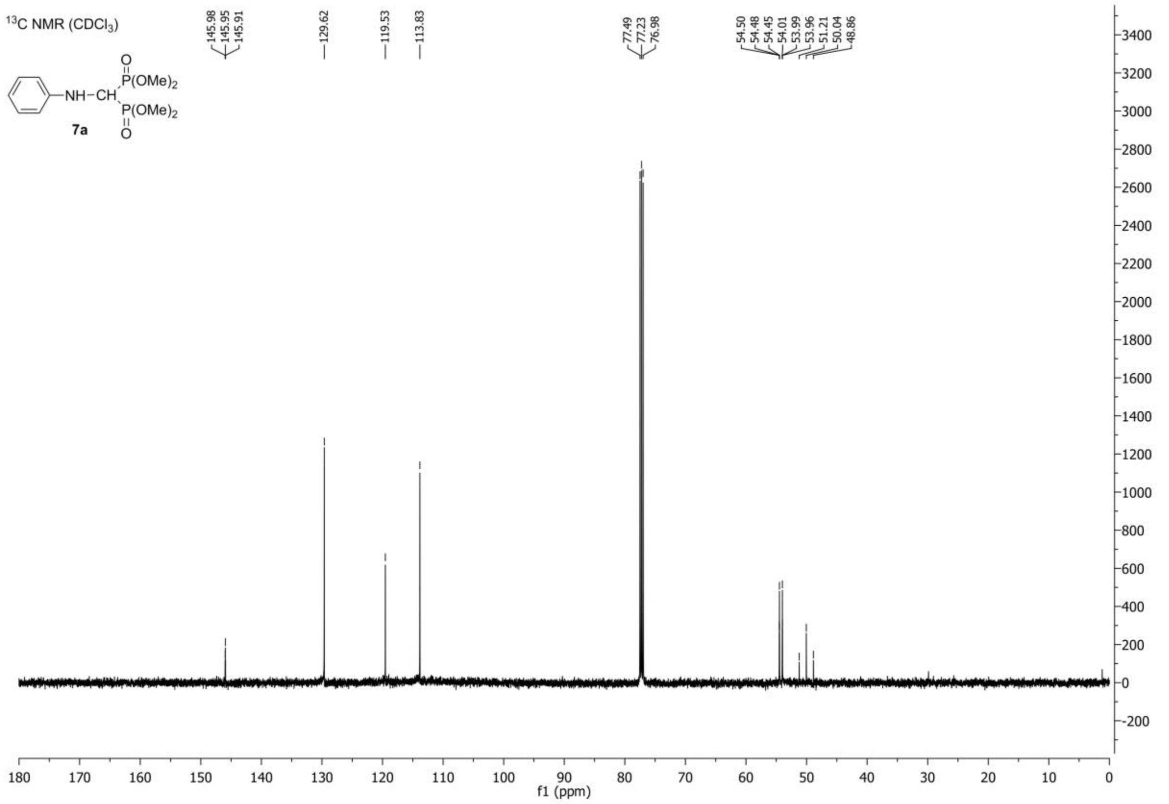

^{13}C NMR (CDCl$_3$)
7a
f1 (ppm)

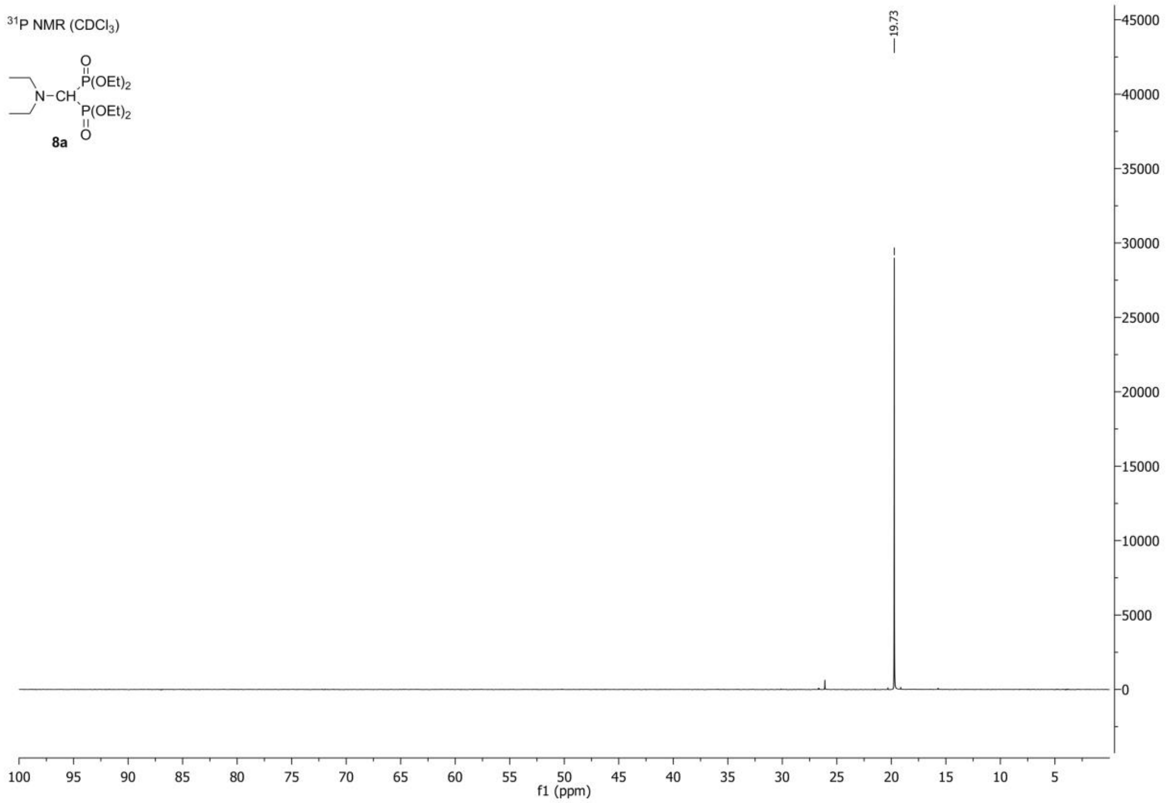

^{31}P NMR (CDCl$_3$)
8a
19.73
f1 (ppm)

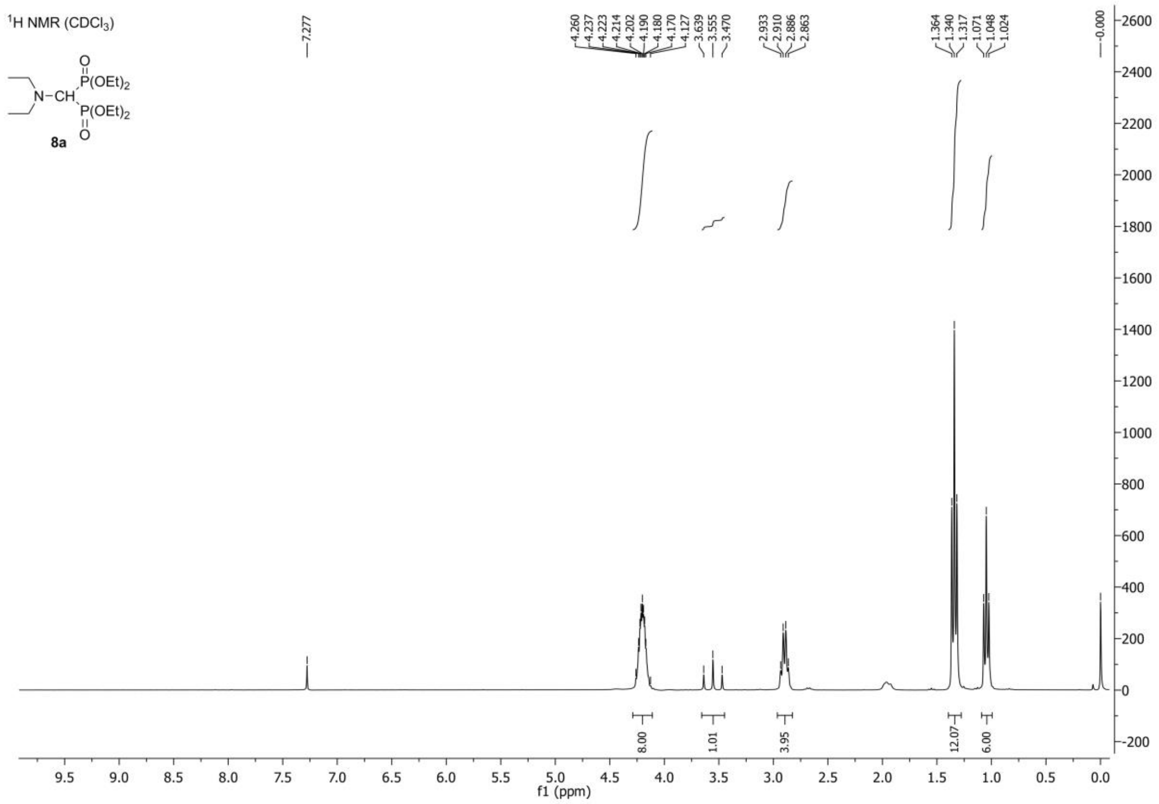

^{1}H NMR (CDCl$_3$)
8a
f1 (ppm)

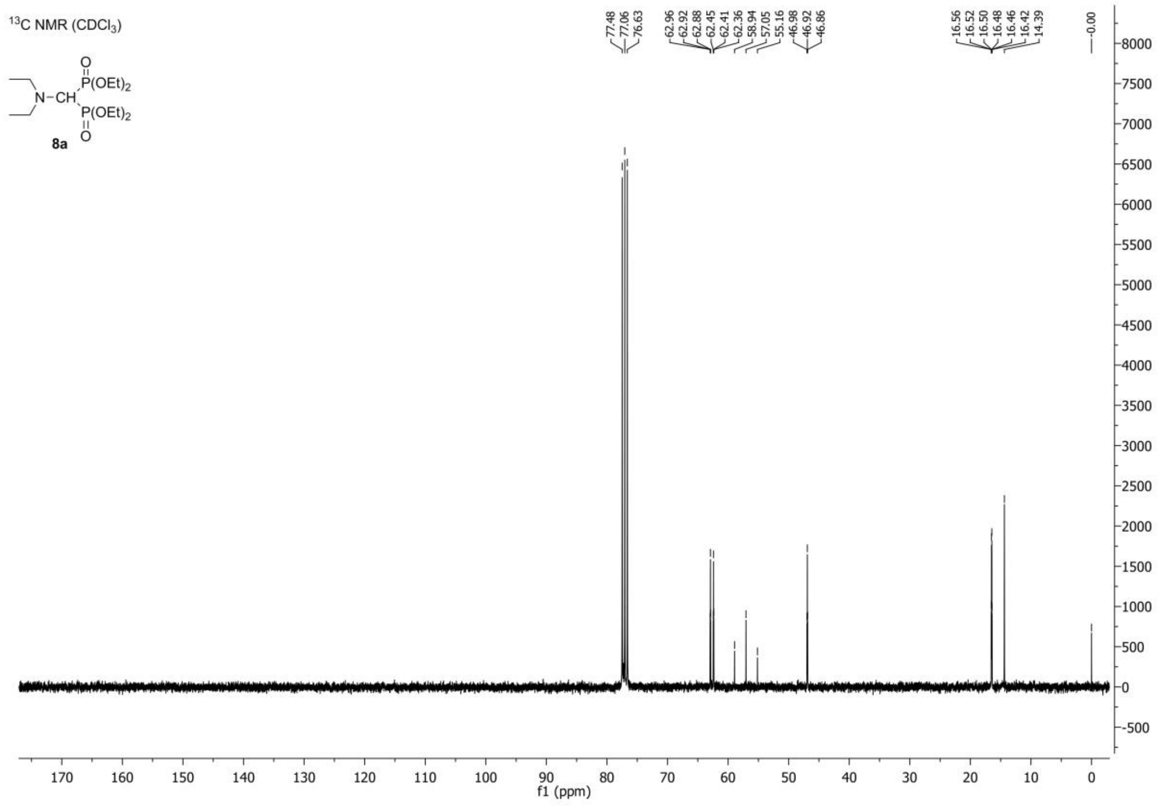

^{13}C NMR (CDCl$_3$)
8a
f1 (ppm)

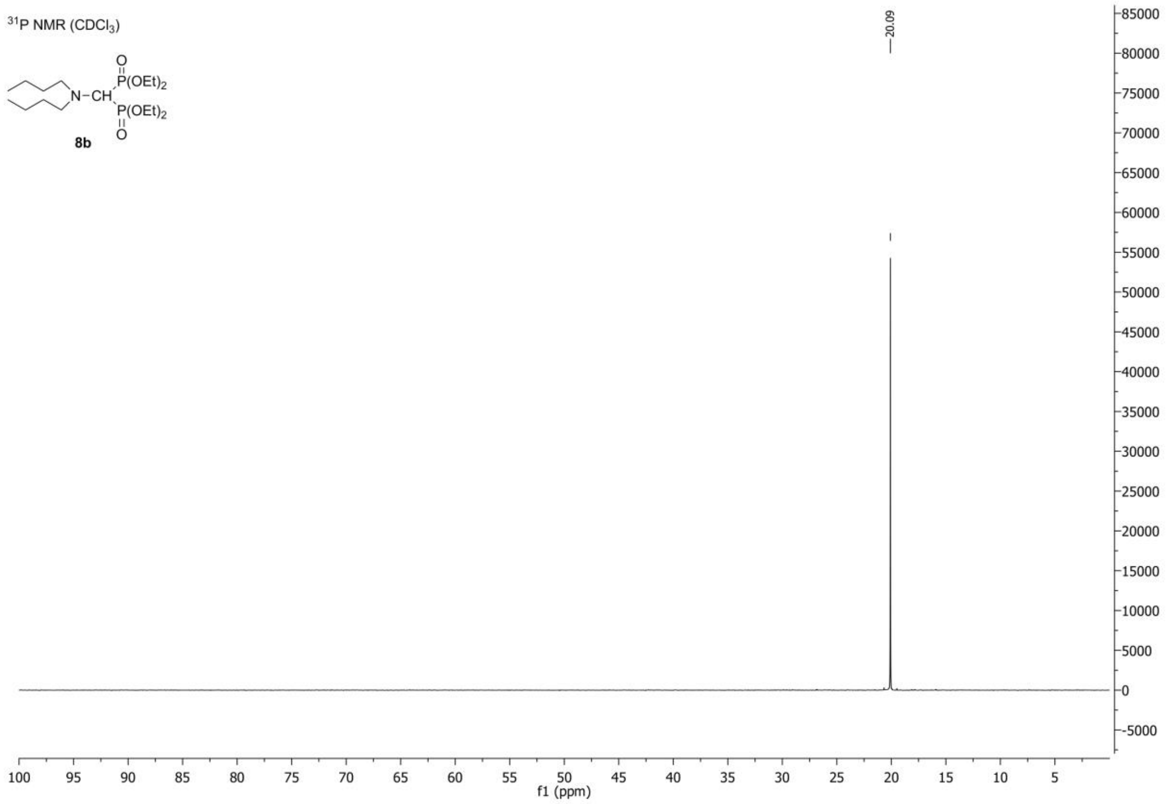

^{31}P NMR ($CDCl_3$)
P(OEt)2
N−CH
P(OEt)2
8b
20.09
f1 (ppm)

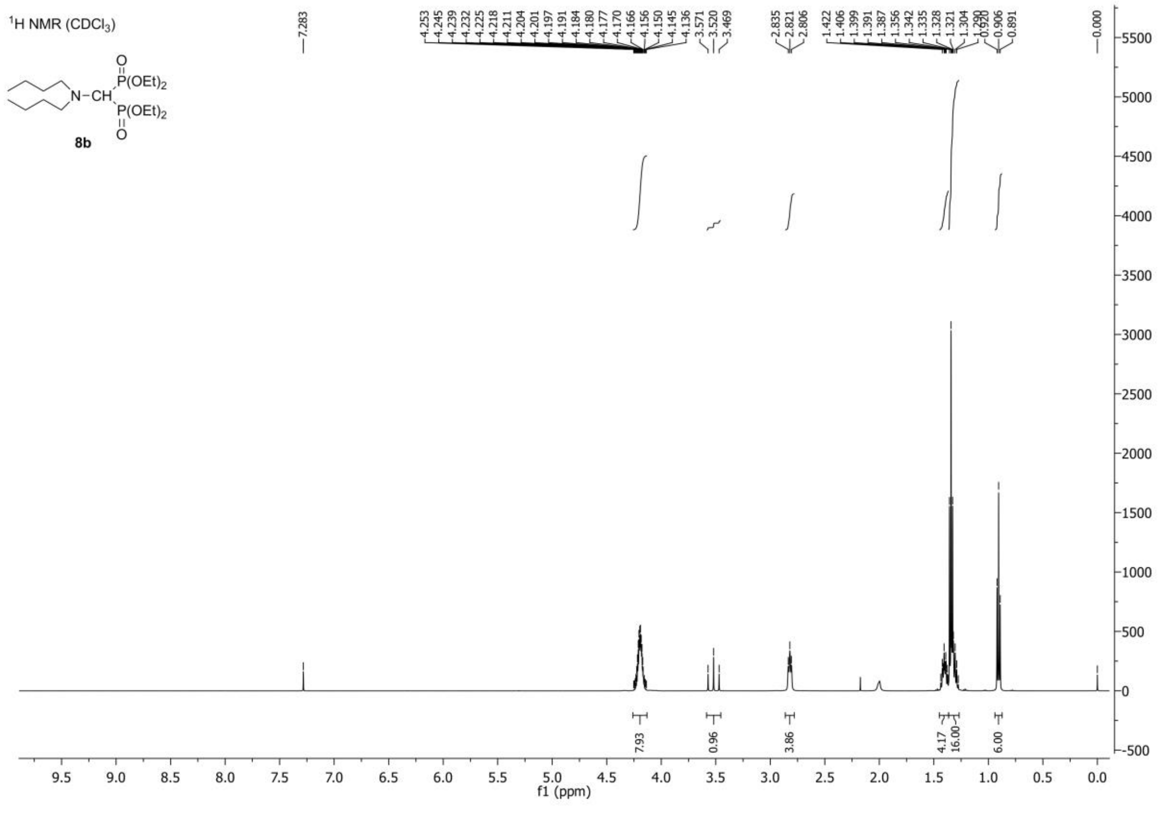

^{1}H NMR ($CDCl_3$)
P(OEt)2
N−CH
P(OEt)2
8b
7.283
4.253 4.245 4.239 4.232 4.225 4.218 4.211 4.204 4.201 4.197 4.191 4.184 4.180 4.177 4.170 4.166 4.156 4.150 4.145 4.136
3.571 3.520 3.469
2.835 2.821 2.806
1.422 1.406 1.399 1.391 1.387 1.356 1.342 1.335 1.328 1.321 1.304
0.906 0.891
0.000
7.93 0.96 3.86 4.17 16.00 6.00
f1 (ppm)

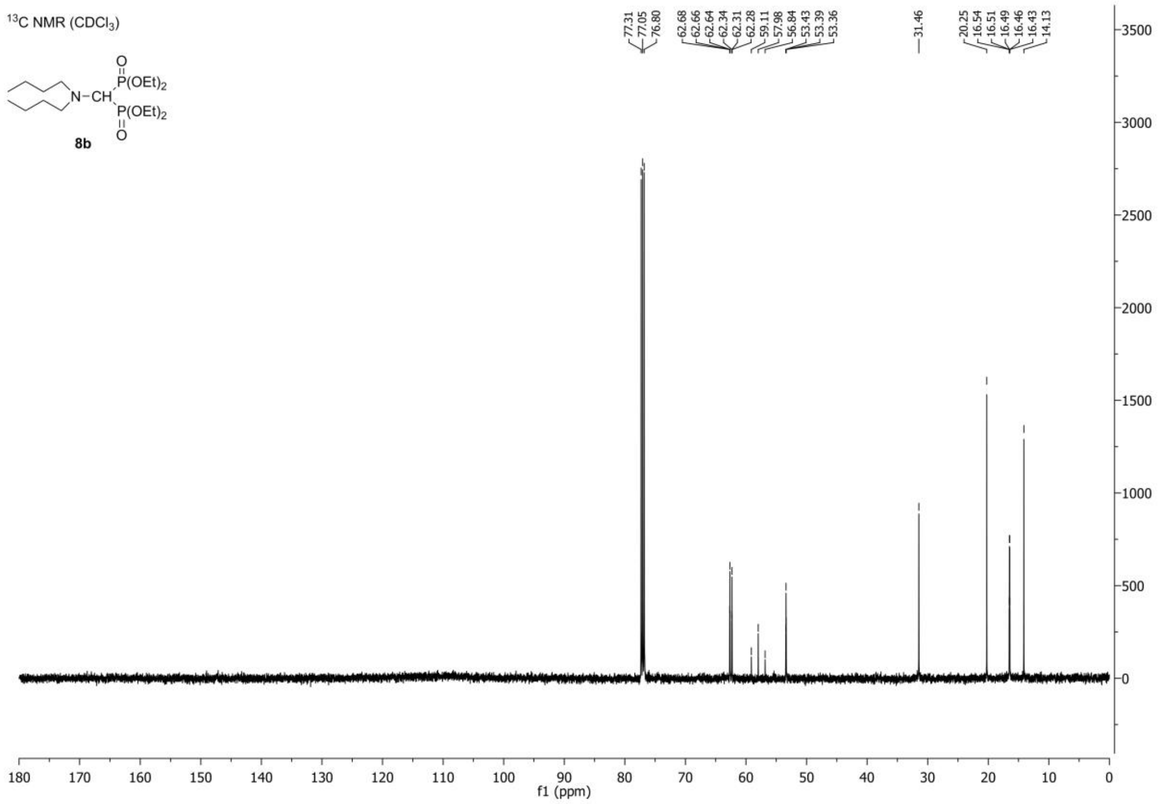
^{13}C NMR (CDCl$_3$)
8b
77.31
77.05
76.80
62.68
62.66
62.64
62.34
62.31
62.28
59.11
57.98
56.84
53.43
53.39
53.36
31.46
20.25
16.54
16.51
16.49
16.46
16.43
14.13
f1 (ppm)

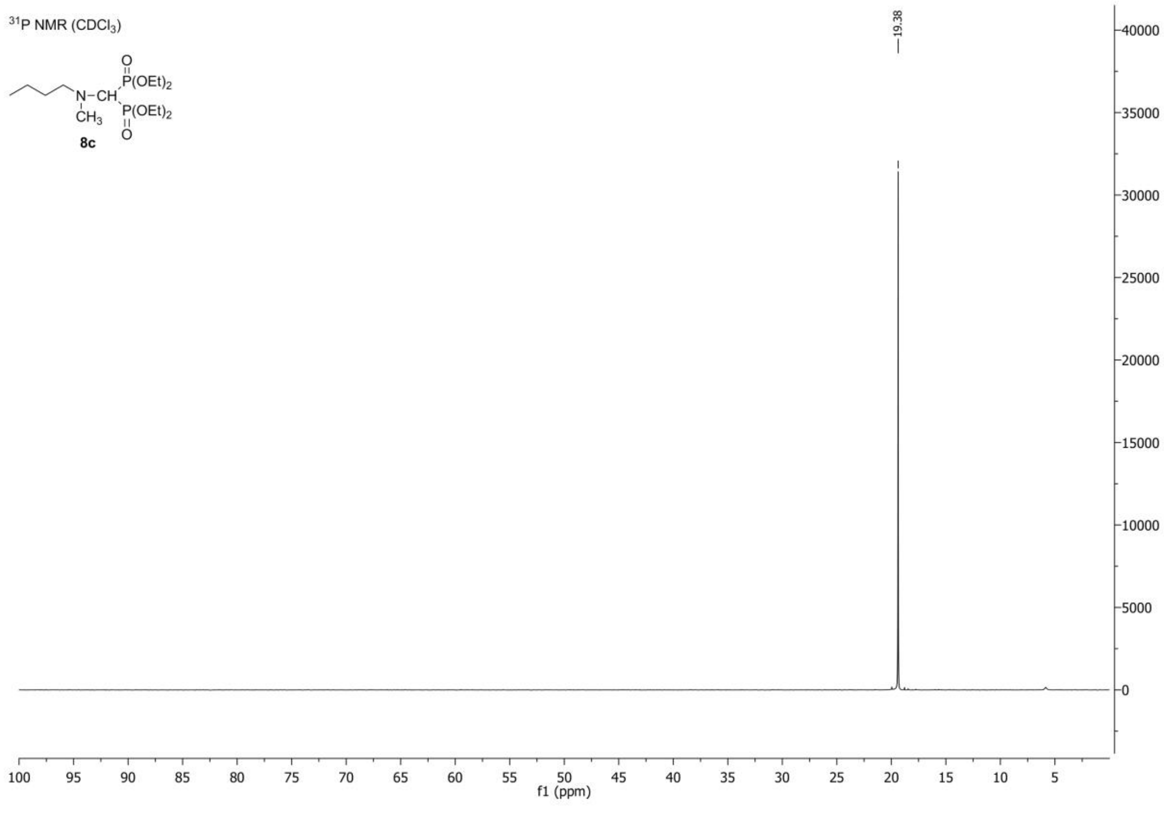
^{31}P NMR (CDCl$_3$)
8c
19.38
f1 (ppm)

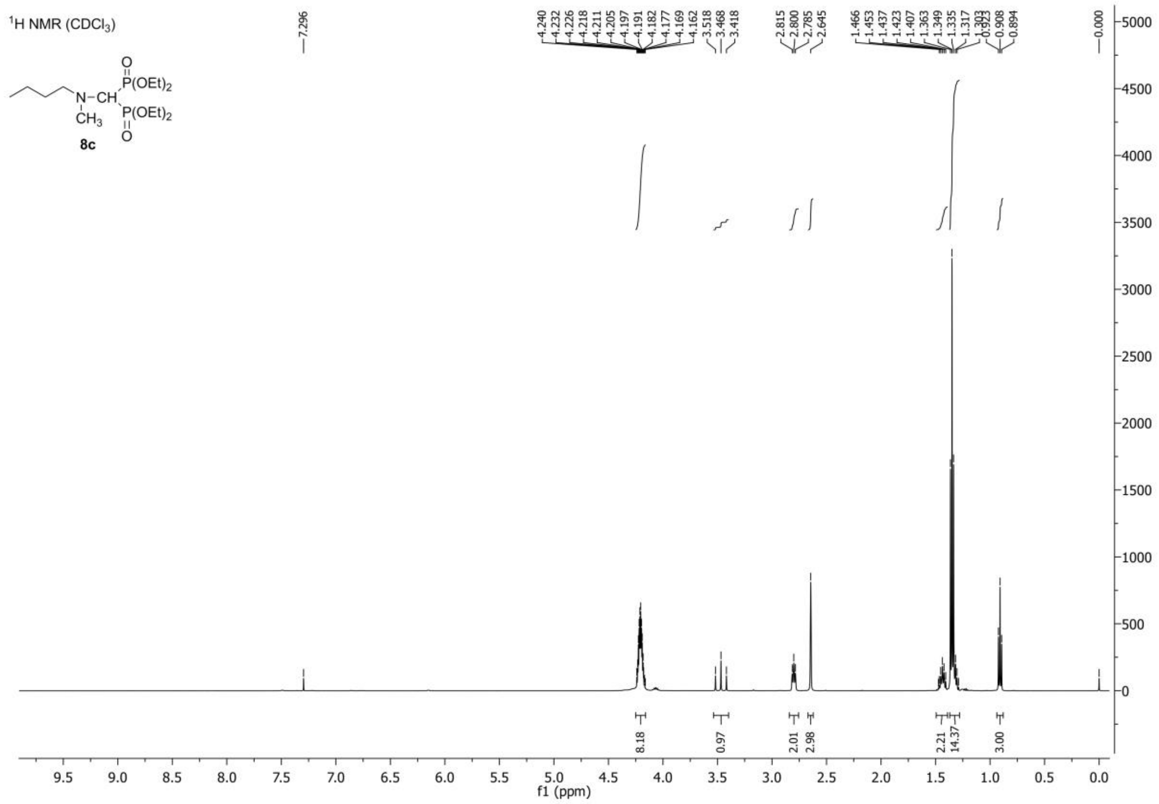

^{1}H NMR (CDCl$_3$)
8c
7.296
4.240 4.232 4.226 4.218 4.211 4.205 4.197 4.191 4.182 4.177 4.169 4.162 3.518 3.468 3.418
2.815 2.800 2.785 2.645
1.466 1.453 1.437 1.423 1.407 1.363 1.349 1.335 1.317 0.908 0.894
0.000
8.18 0.97 2.01 2.98 2.21 14.37 3.00
f1 (ppm)

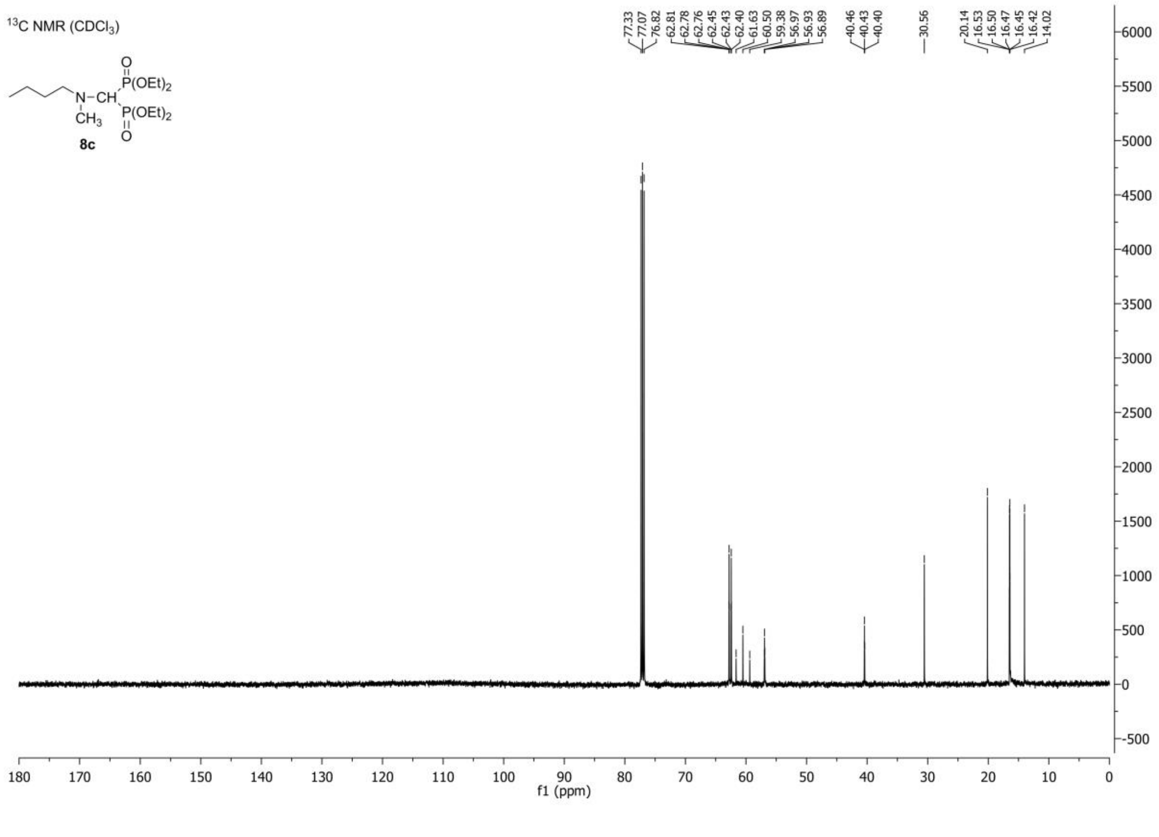

^{13}C NMR (CDCl$_3$)
8c
77.33 77.07 76.82 62.81 62.78 62.76 62.45 62.43 62.40 61.63 60.50 59.38 56.97 56.93 56.89
40.46 40.43 40.40
30.56
20.14 16.53 16.50 16.47 16.45 16.42 14.02
f1 (ppm)

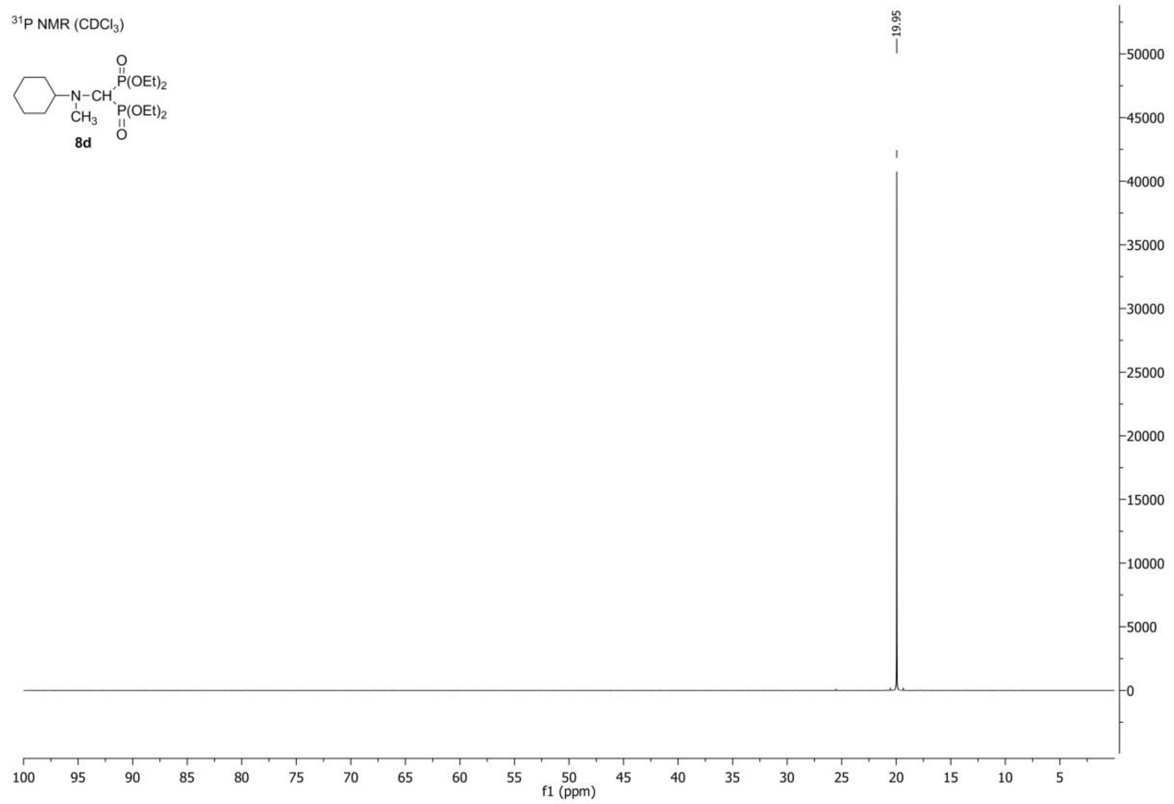
^{31}P NMR ($CDCl_3$)
8d
19.95
f1 (ppm)

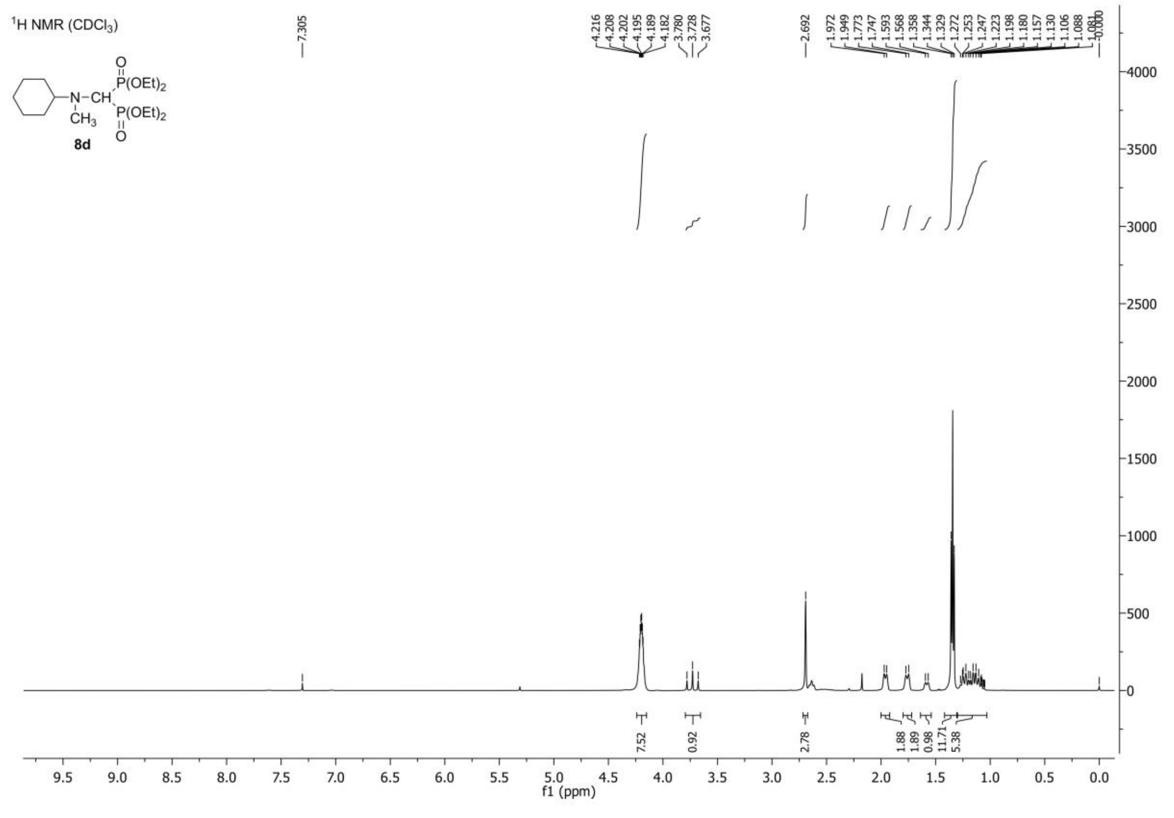
^{1}H NMR ($CDCl_3$)
8d
7.305
2.692
7.52
0.92
2.78
1.88
1.89
0.98
11.71
5.38
f1 (ppm)

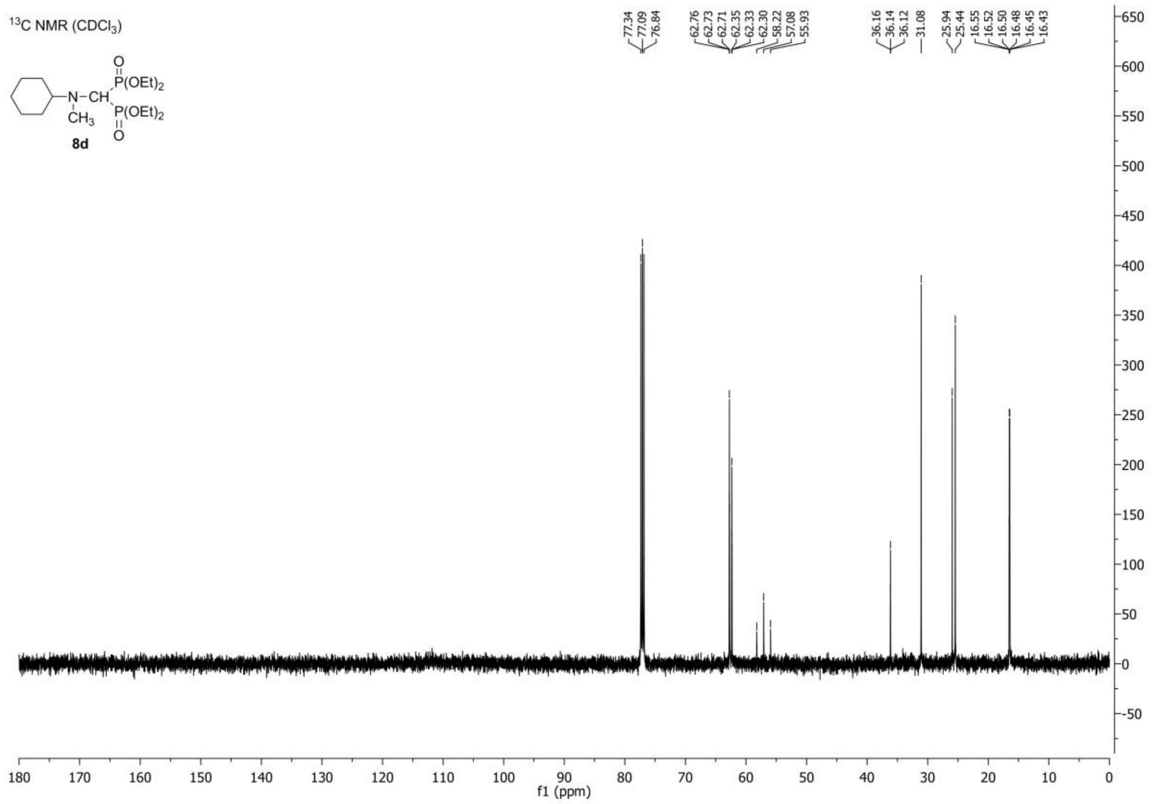

^{13}C NMR ($CDCl_3$)
8d
77.34
77.09
76.84
62.76
62.73
62.71
62.35
62.33
62.30
58.22
57.08
55.93
36.16
36.14
36.12
31.08
25.94
25.44
16.55
16.52
16.50
16.48
16.45
16.43
f1 (ppm)

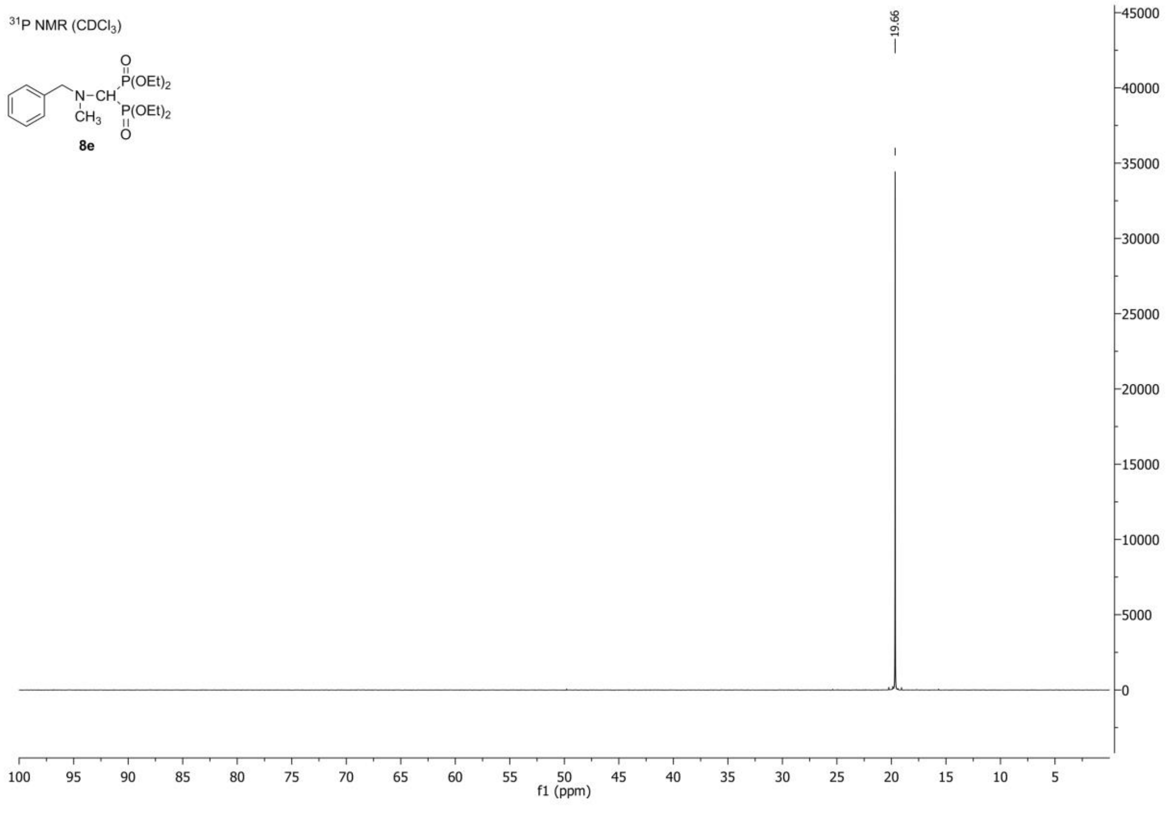

^{31}P NMR ($CDCl_3$)
8e
19.66
f1 (ppm)

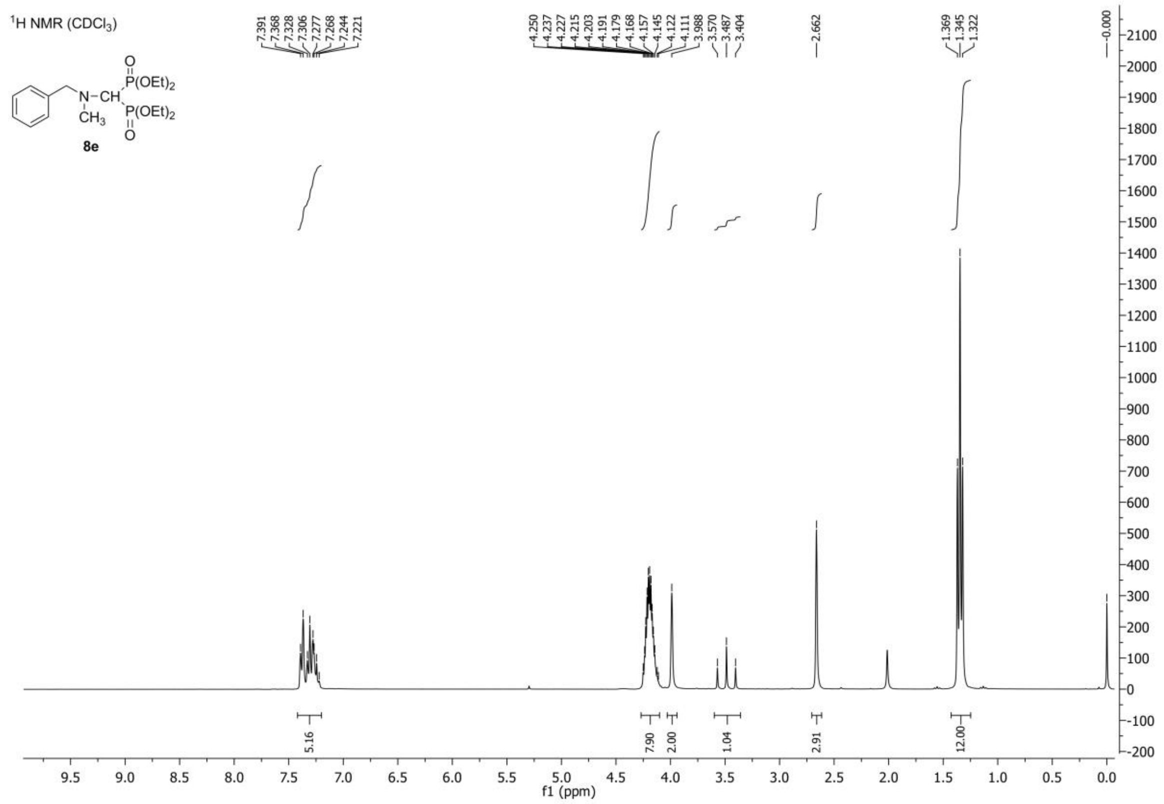

^{1}H NMR (CDCl3)
P(OEt)2
N-CH
CH3
P(OEt)2
8e
7.391
7.368
7.328
7.306
7.277
7.268
7.244
7.221
4.250
4.237
4.227
4.215
4.203
4.191
4.179
4.168
4.157
4.145
4.122
4.111
3.988
3.570
3.487
3.404
2.662
1.369
1.345
1.322
0.000
5.16
7.90
2.00
1.04
2.91
12.00
f1 (ppm)

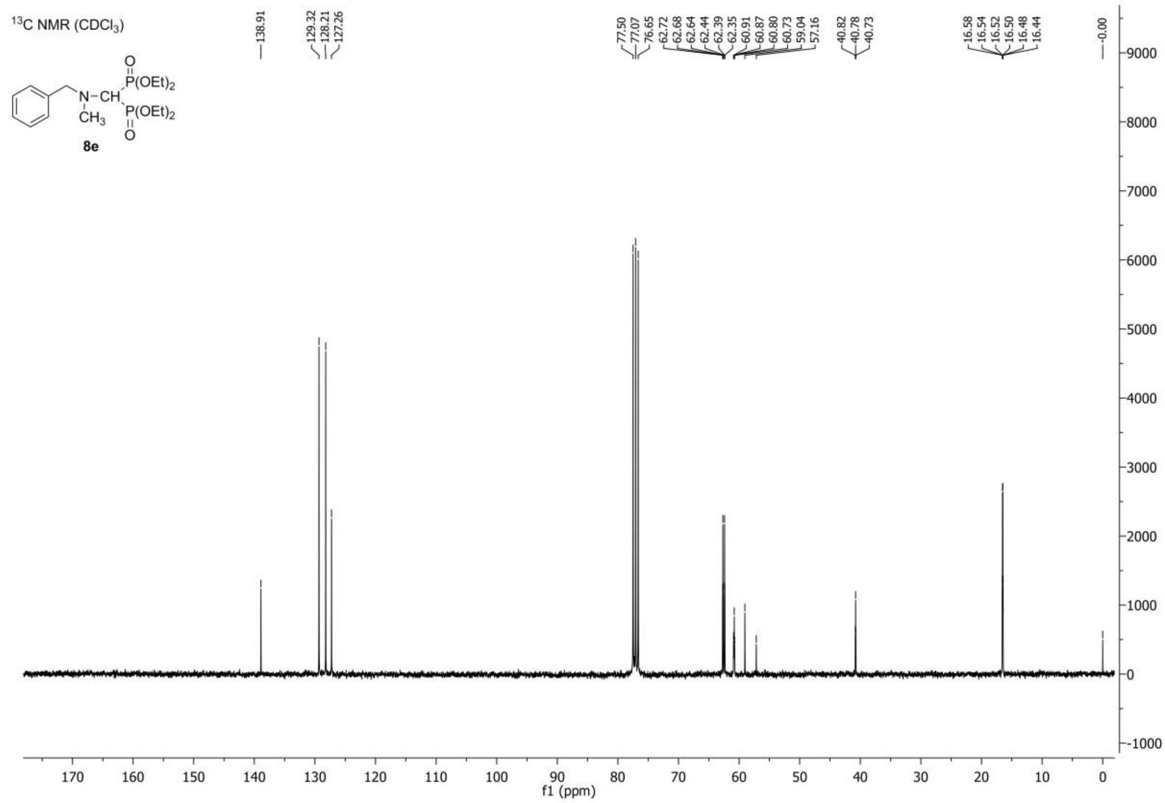

^{13}C NMR (CDCl3)
P(OEt)2
N-CH
CH3
P(OEt)2
8e
138.91
129.32
128.21
127.26
77.50
77.07
76.65
62.72
62.68
62.64
62.44
62.39
62.35
60.91
60.87
60.80
60.73
59.04
57.16
40.82
40.78
40.73
16.58
16.54
16.52
16.50
16.48
16.44
0.00
f1 (ppm)

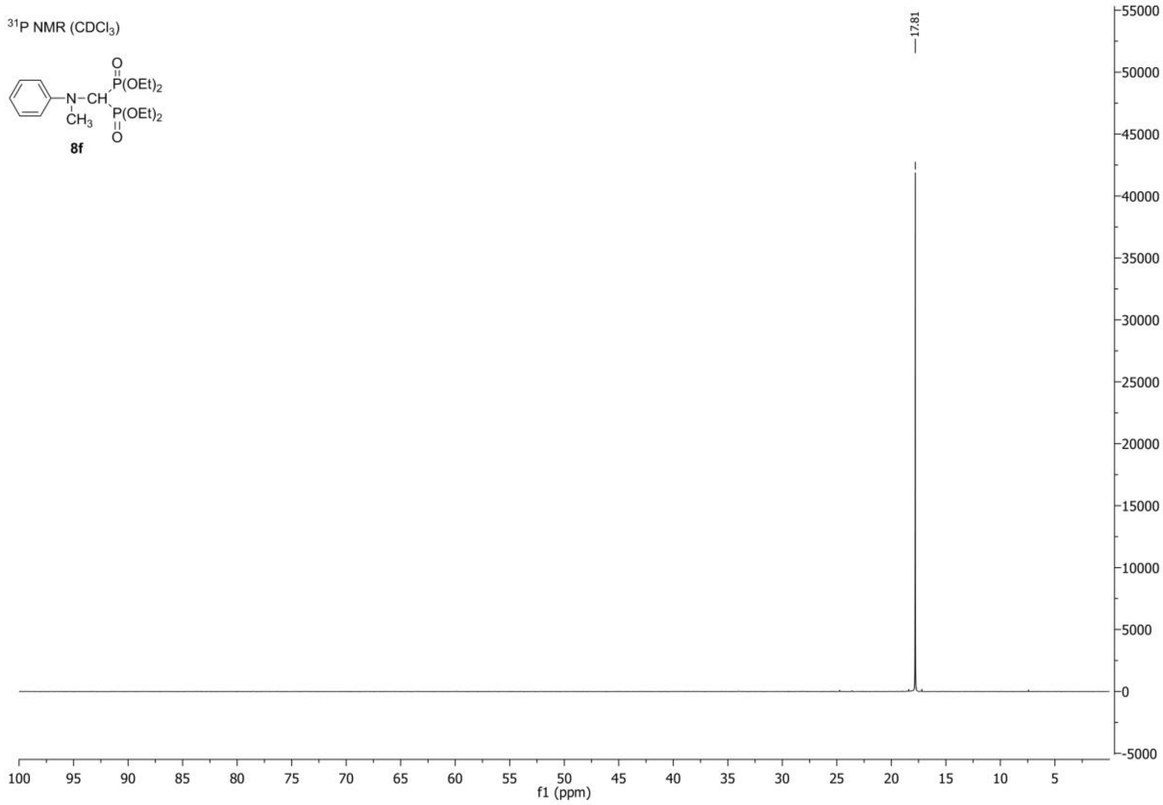

^{31}P NMR ($CDCl_3$)
8f
17.81
f1 (ppm)

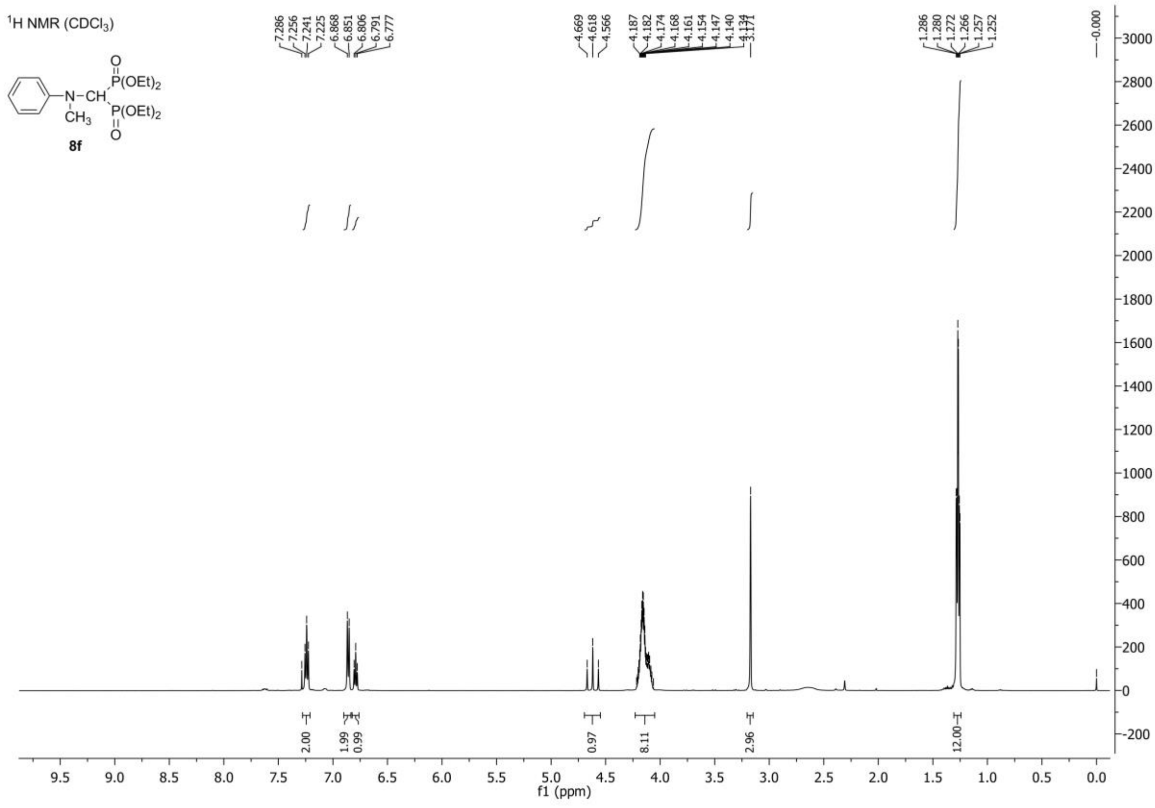

^{1}H NMR ($CDCl_3$)
8f
7.286 7.256 7.241 7.225 6.868 6.851 6.806 6.791 6.777
4.669 4.618 4.566
4.187 4.182 4.174 4.168 4.161 4.154 4.147 4.140 4.134
3.171
1.286 1.280 1.272 1.266 1.257 1.252
-0.000
2.00 1.99 0.99 0.97 8.11 2.96 12.00
f1 (ppm)

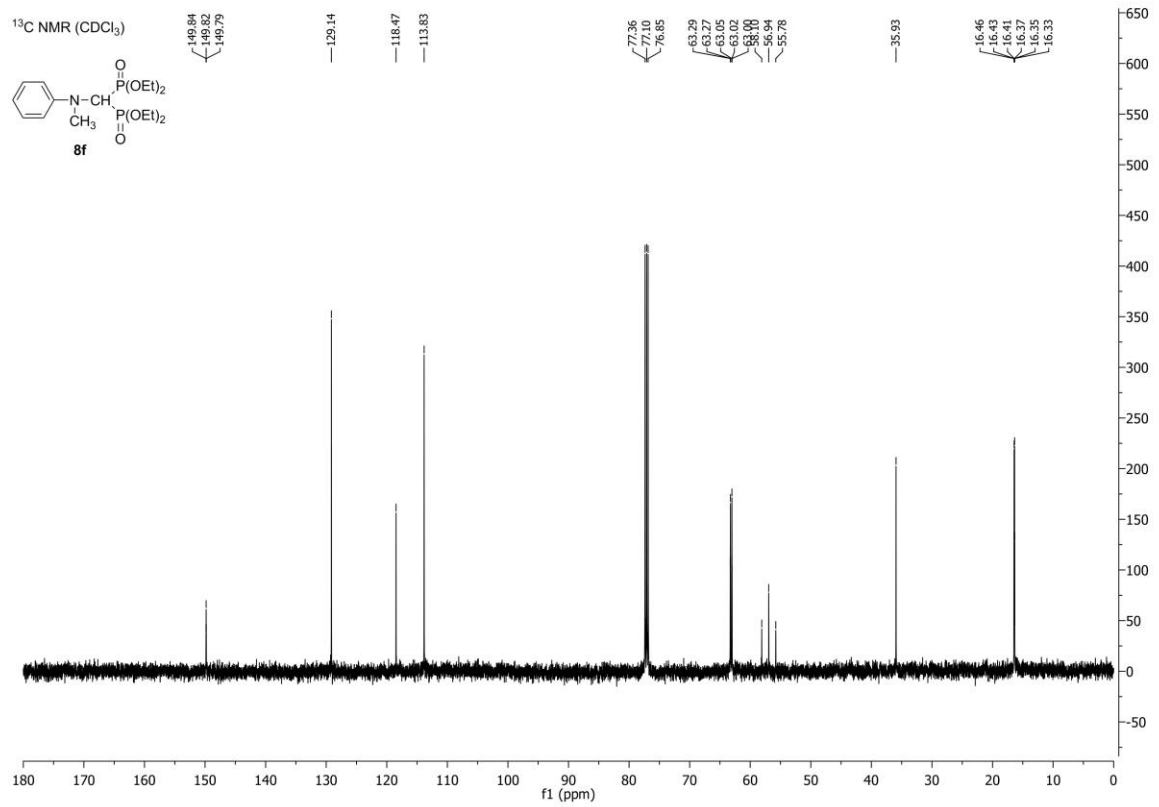

^{13}C NMR (CDCl$_3$)
8f
149.84
149.82
149.79
129.14
118.47
113.83
77.36
77.10
76.85
63.29
63.27
63.05
63.02
63.00
58.10
56.94
55.78
35.93
16.46
16.43
16.41
16.37
16.35
16.33
f1 (ppm)

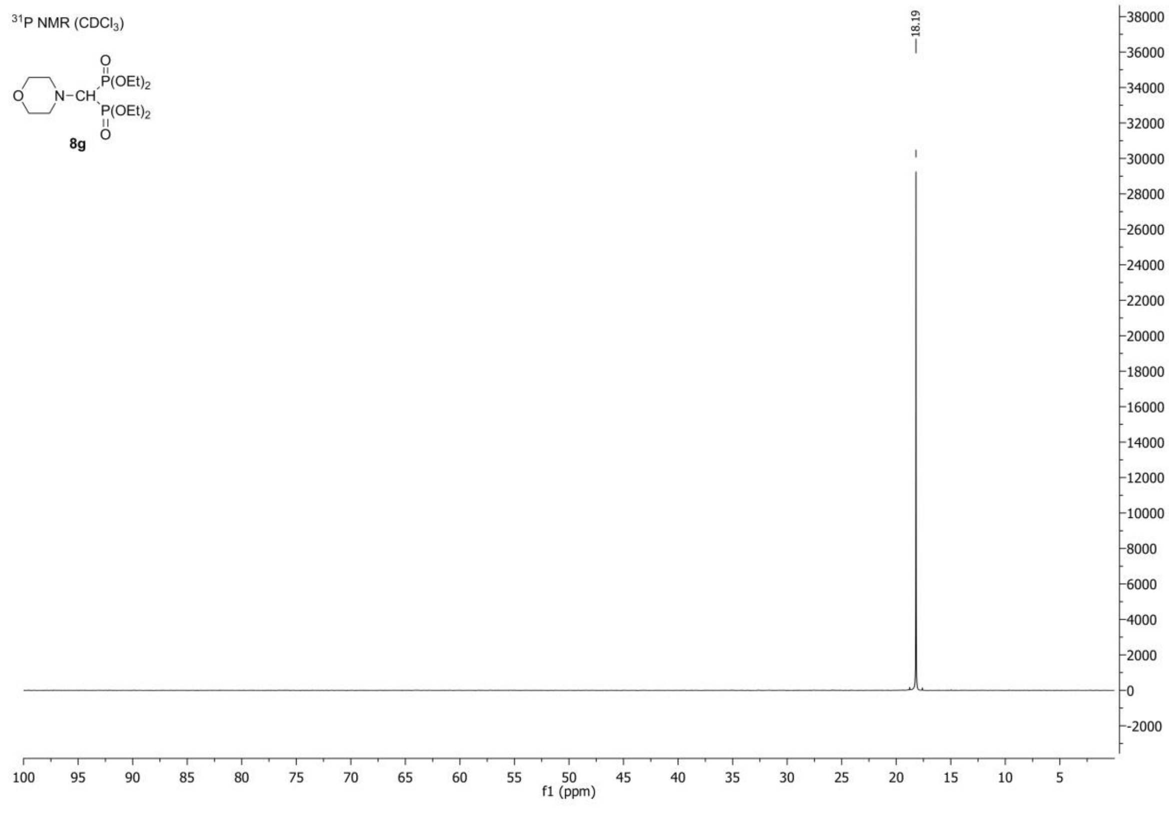

^{31}P NMR (CDCl$_3$)
8g
18.19
f1 (ppm)

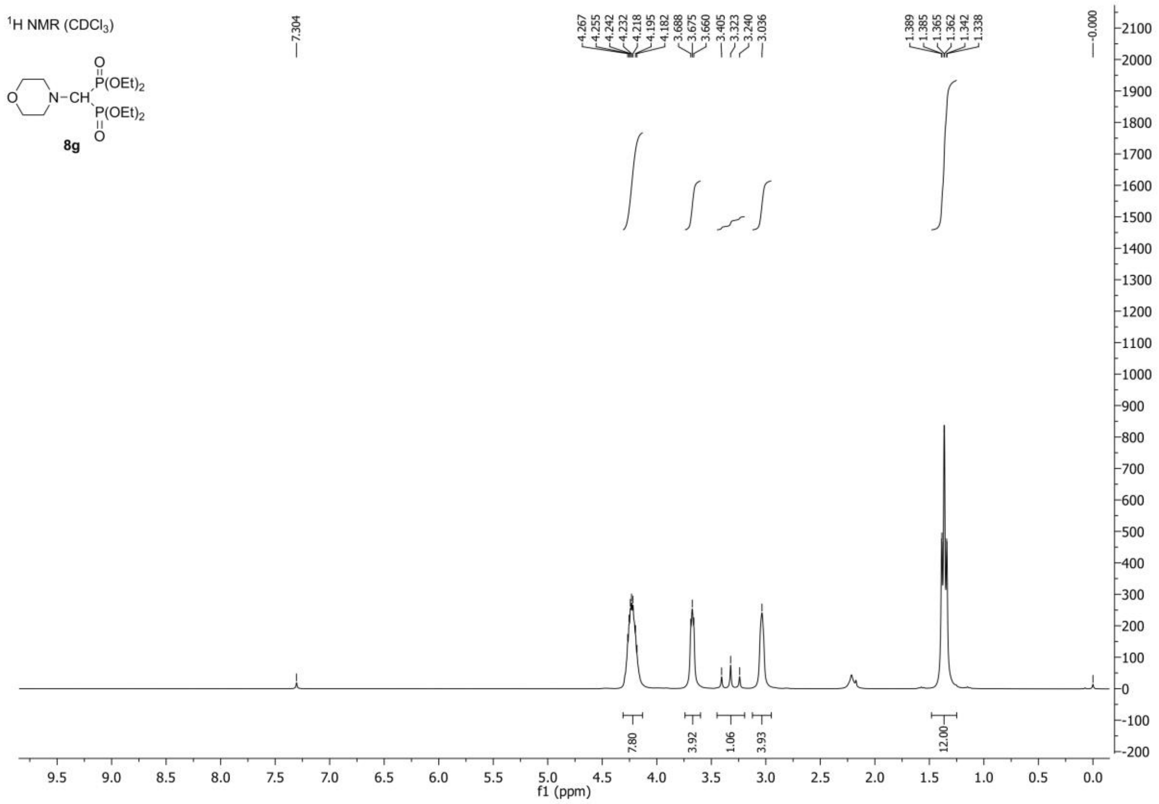

^1H NMR ($CDCl_3$)
8g
7.304
4.267
4.255
4.242
4.232
4.218
4.195
4.182
3.688
3.675
3.660
3.405
3.323
3.240
3.036
1.389
1.385
1.365
1.362
1.342
1.338
0.000
7.80
3.92
1.06
3.93
12.00
f1 (ppm)

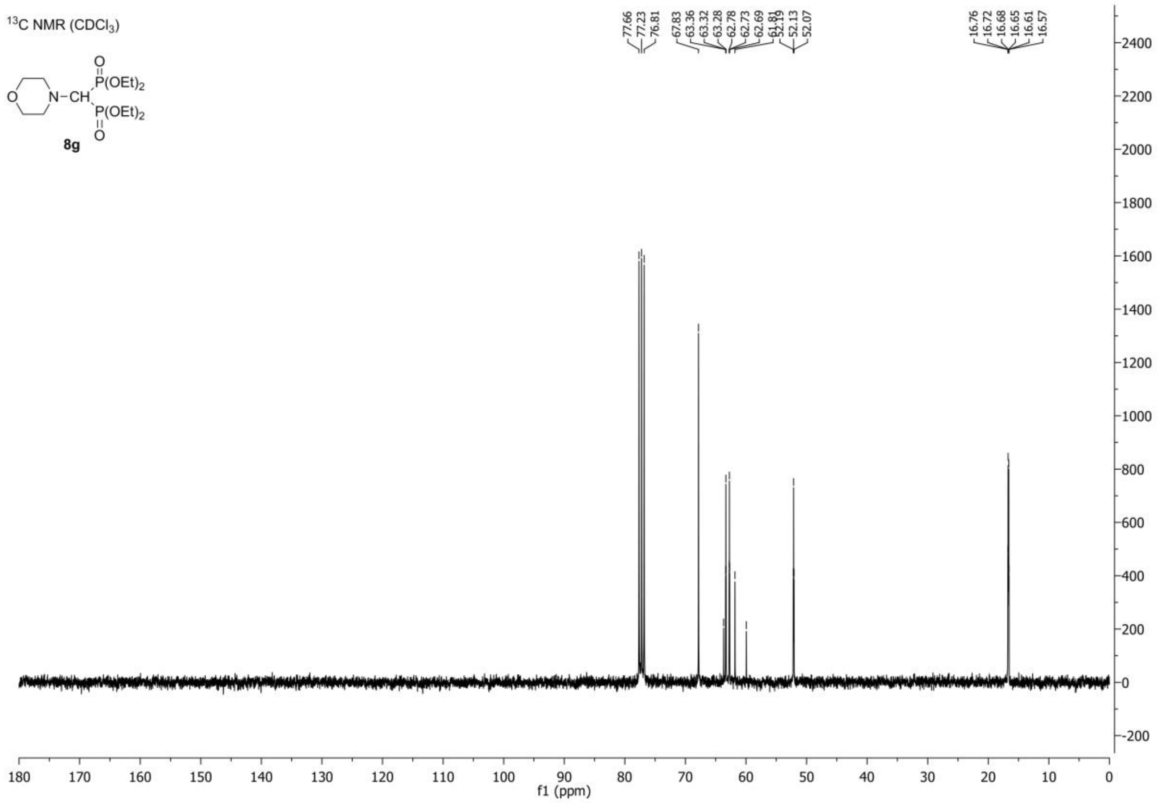

^{13}C NMR ($CDCl_3$)
8g
77.66
77.23
76.81
67.83
63.36
63.32
63.28
62.78
62.73
62.69
61.81
52.19
52.13
52.07
16.76
16.72
16.68
16.65
16.61
16.57
f1 (ppm)

References

S1 Pretsch, E., Badertscher, M., Buhlmann, P. *Structure Determination of Organic Compounds Tables of Spectral Data*, 4th ed.; Springer Verlag Berlin Heidelberg, 2009.

S2 Morgalyuk, V. P.; Strelkova, T. V.; Nifant´ev, E. E. *Russ. Chem. Bull. Int. Ed.* **2012**, *61*, 380–385.

S3 Takeuchi, M.; Sakamoto, S.; Yoshida, M.; Abe, T.; Isomura, Y. *Chem. Pharm. Bull.* **1993**, *41*, 688–693.

S4 Prishchenko, A. A.; Livantsov, M. V.; Novikova, O. P.; Livantsova, L. I. *Russ. J. Gen. Chem.* **2009**, *79*, 1936–1938.

S5 Pudovik, A. N.; Nikitina, V. I.; Zimin, M. G.; Vostretsova, N. L. *J. Gen. Chem. USSR*, **1975**, *45*, 1450–1455.

S6 Ekimoto, H. Metal Complex Compound, Cancer Therapeutic Composition Comprising the Metal Complex Compound as Active Ingredient, and Intermediate for Production of the Metal Complex Compound. European Patent 20080790864, April 21, 2010.
